# Invisible Brain: Knowledge in Research Works and Neuron Activity

**DOI:** 10.1371/journal.pone.0158590

**Published:** 2016-07-20

**Authors:** Aviv Segev, Dorothy Curtis, Sukhwan Jung, Suhyun Chae

**Affiliations:** 1 Graduate School of Knowledge Service Engineering, KAIST, 291 Daehak-ro, Yuseong-gu, 305-701, South Korea; 2 School of Computing, KAIST, 291 Daehak-ro, Yuseong-gu, 305-701, South Korea; 3 CSAIL, MIT, 32 Vassar St, Cambridge, MA, 02139, United States of America; Lanzhou University of Technology, CHINA

## Abstract

If the market has an invisible hand, does knowledge creation and representation have an “invisible brain”? While knowledge is viewed as a product of neuron activity in the brain, can we identify knowledge that is outside the brain but reflects the activity of neurons in the brain? This work suggests that the patterns of neuron activity in the brain can be seen in the representation of knowledge-related activity. Here we show that the neuron activity mechanism seems to represent much of the knowledge learned in the past decades based on published articles, in what can be viewed as an “invisible brain” or collective hidden neural networks. Similar results appear when analyzing knowledge activity in patents. Our work also tries to characterize knowledge increase as neuron network activity growth. The results propose that knowledge-related activity can be seen outside of the neuron activity mechanism. Consequently, knowledge might exist as an independent mechanism.

## Introduction

Neuron activity has been researched extensively in the area of knowledge representation. Previous work suggested that there is another layer between neuron activity and knowledge representation in brain activity [1, 2]. In economics, the invisible hand of the market is a metaphor to describe the self-regulating behavior of the marketplace [3]. Our work shows that the neuron-like behavior appears in knowledge systems outside of the brain, therefore suggesting that knowledge can be represented directly by patterns of neuron activity as an “invisible brain”. Thus we suggest that biological neuron behaviors are a good tool for the representation of knowledge activity and might appear in multiple areas of life.

The biological neuron model describes the mathematical neuron spiking process. Neurons are viewed as cells specialized for communication with other neurons or cell types. Biological neuron communication is performed through synapses, which are electrical or electrochemical signal junctions. We conceptualize knowledge in research as spiking neuron communication and compare the research publication activity to real neuron behavior.

The method is based on identifying similarity between brain neuron behavior and research publication activity over time, which can be considered a representation of knowledge. We analyze 9,799,239 research publications as well as patents in different domains according to topics such as anaphylaxis, jet turbine, and game theory and identify the research areas over 45 years. We extract the communication activity between different research topics according to keyword frequency correlation appearing in published articles. Then we map all research topic activity to keyword frequency correlation and compare the resulting correlation to biological neuron activity simulations. The communication is monitored over time for spiking activity as in collective hidden neural networks. Similar communication in patents is analyzed to check whether the knowledge activity appears in other areas.

### Knowledge and Brain

The topic of knowledge and brain activity has been researched ever since Plato stated that “knowledge is perception” [4] and speculated that knowledge is contained in the brain [5]. The hypothesis that the functional unit of the brain is the neuron was formed based on the development of a staining procedure by Golgi that used silver chromate salt to reveal the intricate structures of a single neuron. Cajal’s use of the technique led to the formation of the neuron doctrine, the hypothesis that the functional unit of the brain is the neuron [6].

Currently the neuron doctrine views the brain as a network [7]. There are many structure-function relationships in a brain. One can view a set of regions as a set of nodes while a relationship between two regions is mapped to a link between corresponding nodes. How we can determine each region is an issue which corresponds to building nodes in a network.

Previous work in graph theory employed a clustering coefficient as a measure of the degree to which nodes in a graph tend to cluster together. Evidence indicated that in most real-world networks, and especially in social networks, nodes tend to form tightly knit groups marked by a relatively high density of ties which tends to be greater than the average probability of a tie randomly established between two nodes [8, 9].

The structure of a dynamic network, or evolving network, can alter as time passes. In some cases, we separate a period of time into several time slots, and we can view a dynamic network as a sequence of networks, where each network represents a snapshot of the dynamic network at each time slot [10, 11].

For a given complex system, there are actuators of the system and there are interactions, interconnections, or relationships among them. Examples include social networks [9], collaboration networks [12], the Internet [13], the World Wide Web [14], and biological networks [15]. Many complex networks for real-world systems have the same properties, such as small world phenomena and power-law distribution; two nodes in a network are likely connected through a short path (a sequence of nodes of a network), and the degree (the number of neighbors of a node) follows a power-law distribution so that there exist some hub nodes which have many more connections than others.

Many methods have been developed for the analysis of homogeneous networks. But the analysis of heterogeneous networks is not simple, for links across entities can have several types. The Internet of Things is an example of a heterogeneous network [16]. The approach uses a variety of things or objects which can be represented as nodes. The nodes can be connected by their relations. Another approach is the network of networks, which can be viewed as a system of coupled networks, where the networks have different nodes or multilayer networks and networks can be layered when each layer contains the same type of edges in the presence of multiple types of edges [17].

Another problem is how to build a network for a given complex system. The problem is closely related to the problem of graph drawing or network visualization [18], where there is a suitable node-link diagram that describes a network. However, the graph drawing problem usually assumes that a set of nodes and a set of edges are given together and tries to find a set of positions of the nodes in a space. Other work addressed the problem of building a complex system by merging one network that contains the link structure and another network that contains the content information [19].

Evolutionary clustering deals with the problem of processing time stamped data to produce a sequence of clustering for each time step of the data arriving to the detecting system [20]. In analyzing dynamic networks such as social networks, node characteristics and behavior are often correlated with influence and homophily [21].

Optical [22, 23, 24] and electrophysiological [25, 26] techniques exist for recording activity from many neurons. The field of recording a large number of neurons is still in its infancy. Current techniques allow recording only a small number of neurons for imaging. The current techniques are limited compared to the number of neurons existing in the brain of most animals analyzed today. The analysis of only a small number of neurons is limited in the isolation of the boundary of neural network behavior [27, 28].

To analyze neuron network connectivity behavior, the electrical activity between the neurons can be sampled. When sampling neurons, both the spatial resolution and the time resolution should be considered. Previous research has sampled neuron activity extending from milliseconds to months. Regarding the neuron network area covered, the area sampled ranges from a small number of microns to centimeters [29, 30].

Although it is known that neurons are connected by a network structure, how this network works and achieves the results attributed to the brain as a whole is far from being clear. The analysis of the change of fluorescence can assist in the understanding of the activity of neural events and the neural network spiking connectivity. Neural reconstruction can facilitate viewing how large networks of neurons spike and how different spiking areas in the network are associated. The idea behind it is that when the neural network spiking is over-excitable one neuron can trigger a large number of neurons which are not directly connected to it [31]. Previous work has also analyzed neuron spiking relative to background activity [32, 33].

The idea that the connectivity of a neuron system can be generalized across systems in different animals based on existing knowledge of small circuits has been previously suggested [34]. Similarly, previous work suggested clustering knowledge according to passing messages by identifying a subset of processing sensory signal examples and detecting patterns in the data [35].

In the past Hopfield [36] suggested to build collective systems having a large number of simple elements similar in their activity to neurons used by biological organisms. The idea was expanded as a conceptual framework to understand the computational processing in the neural circuit model, where circuits consist of neurons organized in networks with effective synaptic connections [37].

The organization of large populations of interacting elements has been researched extensively in areas of physical, biological, chemical, and social systems. The problem of organization synchronization included the approach of modeling each member of the population as a phase oscillator [38]. Spiking neural systems are viewed as a class of distributed parallel computing devices motivated by the way neurons communicate by means of spikes. Asynchronous systems are non-synchronized systems, where the use of spiking rules is not mandatory [39].

One method used for simulating a large number of neurons was based on video viewing of neuron activity [40]. The method was based on taking snapshots of neuron networks through optical imaging. The video acquired from the time series snapshots allowed the measurement of large networks of neurons. Based on the neuron activity in the video, the structure of the neuron network could be reconstructed. The snapshots of the neuron calcium inflow assist in estimating the resulting actions of the individual spiking neurons. The implemented method analyzed the changing fluorescence values, which measured reactions between fluorescent molecules and calcium ions.

The method used here is based on a simulation that aims at inferring the connectivity of neuron networks from calcium fluorescence imaging of their network signals. The simulation method used [41] is based on analyzing cause and effect of neuron behavior over time. The problem is represented as a graph with nodes, neurons, and edges connecting to other nodes, synapses. The graph is directed where all edges are from one node to another. Therefore, edges can be viewed as either excitatory or inhibitory. The neurons are arranged in a two-dimension “square petri dish”. The coordinate location of the neurons is predetermined and used for both data sets including the neurons and the research activity. The simulation also includes light scattering effects due to overlapping signals.

The data simulation [41] used is based on real neuron behavior. The neuron network structure uses topology connectivity models. The simulation is based on neuron distance and estimates neuron clustering based on real biological data. The method incorporates NEST simulator models [42, 43] for leaky integrate-and-fire models. These models provide a more accurate simulation of experimentally observed neuron spiking recordings. The fluorescence is modeled using time averaging of calcium fluorescence spiking and light scattering. The fluorescence emitted from neurons is compared to the “fluorescence” emitted from knowledge based on the analysis of research publications and patents to view the activity of the “invisible brain”.

### Research Publication Activity

Research publication activity is analyzed by the number of publications on each topic (node) and correlation between topics (node connectivity or “synapses”). The activity on a specific topic can be viewed by the number of article or patent publications on a specific topic analyzed by keyword occurrences. The keyword set used to define each publication can be supplied by the author, the publication journal, or patent classifications based on a predefined set of keywords or extracted from the title or the abstract. The basic time frame for evaluating topic publication activity was set at one year. Smaller time frames were analyzed but seemed less significant due to the periodic timeline of research activity.

Evaluating connectivity, or communication, between research topics is based on identifying first the related topics. This is done by classifying multiple topics which appear in the same research article or patent, as identified by the selected keywords. Once two topics are marked as related, we analyze the degree of correlation of the topics’ activity over the whole time period viewed. Then we can select the top *n* highly correlated nodes to be compared to *n* simulated connected brain neurons and observe the “fluorescence” created by both activities.

PubMed and Web of Science provide access to multiple databases of references and abstracts on life sciences and biomedical topics. The United States Patent and Trademark Office provides information about all patents and intellectual property in the US. To analyze research publication activity, articles and patents were extracted from all three data sources according to general topic keywords such as *anaphylaxis*, *Doppler effect*, *yellow fever*, and *diphtheria*.

**Fig 1:**
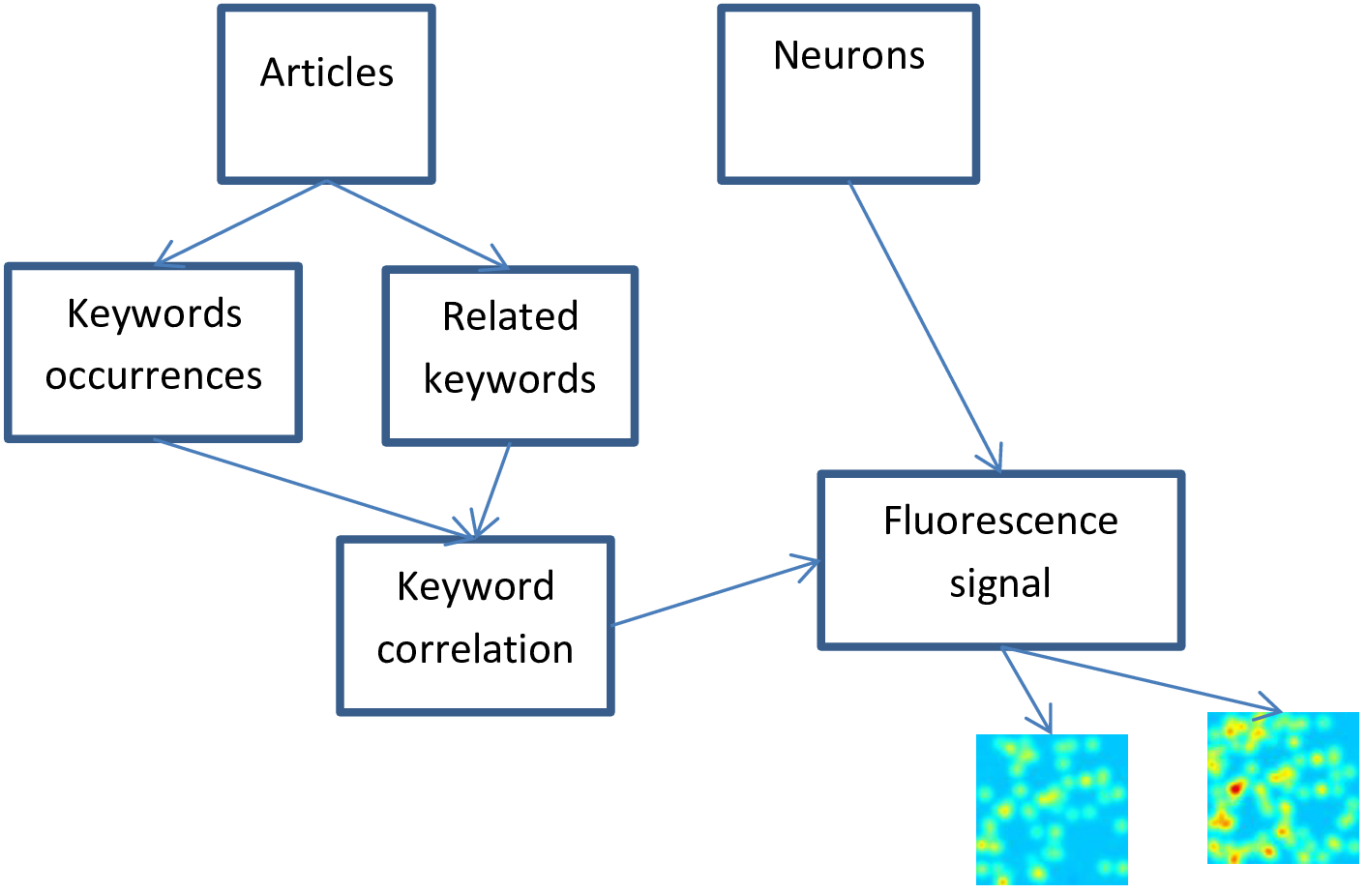
Extracting Florescence from Research Articles and Neurons.

The research topic analysis method includes the following steps outlined in Fig 1:

- Extract the publication year and list of specific topic keywords associated with each article or patent.
- Count the number of appearances per year of each keyword.
- Identify the relation between keywords based on multiple keywords appearing in a single article or patent.
- Calculate correlations changing over time between related keywords.
- Image the changing correlations between research topics as fluorescence signals.
- Compare the fluorescence signals emitted over time between neuron activity and research publication activity.

### Neuron versus Research Activity

Analysis was performed on 9,799,239 research articles and on patents from 24 different topics in medicine and 9 topics related to physics and game theory in over 45 years. The data topic description is displayed in Table 1. Data sets from PubMed, Web of Science (WoS), and United States Patent and Trademark Office (USPTO) were used in the experiments. The number of keywords which can be used ranges from a single term per article or patent to all possible words in the abstract. Two keyword search field tags were used from each data source. Web of Science has WC (Web of Science Category) and SC (Subject Category). PubMed has MH (MeSH—Medical Subject Headings Terms) and OT (Other Term). USPTO has CPC (Cooperative Patent Classifications) and ICL (International Classification). The total number of article or patent records used ranged from 182 (*quantum electro dynamics*) to 471,641 (*diabetes*). Except for topics such as epidemiology, microbiology, oncology, and genetics, which were too big, the input data can be found in the Supporting Information [S1–S9 Files]. Preprocessing was performed to remove duplications. The results were organized by descending correlation.

**Table 1:** Data of 24 medicine and 9 physics topics analyzed.

**Topic (Medicine)**	**Records**	**Data Source**
Irritable Bowel Syndrome	12,768	WoS
	8,893	PubMed
In Vitro Fertilization	29,005	WoS
	36,939	PubMed
Angina	57,216	Pubmed
Nephrology	80,920	Pubmed
Hematology	124,883	PubMed
Ophthalmology	142,924	Pubmed
Hepatitis	189,561	Pubmed
Orthopedic	191,934	Pubmed
Infectious disease	163,816	Pubmed
Diphtheria	8,680	WoS
	12,099	PubMed
	12,717	USPTO
Gastritis	16,081	WoS
	25,045	PubMed
Epidemiology	1,743,446	Pubmed
Hypoglycemia	34,033	Pubmed
Typhus	1,932	WoS
	2,836	PubMed
Poliomyelitis	3,999	WoS
	9,705	PubMed
Anaphylaxis	12,353	WoS
	19,299	PubMed
Microbiology	1,044,080	Pubmed
Oncology	2,037,722	Pubmed
Diabetes	471,641	Pubmed
Genetics	2,821,631	Pubmed
Obesity	206,555	Pubmed
Obstetrics	203,450	Pubmed
Yellow Fever	4,122	WoS
	3,824	PubMed
**Topic (Physics)**	**Records**	**Data Source**
Quantum Electro Dynamics	182	WoS
Antimatter	1,107	WoS
Jet Turbine	1,471	WoS
Bubble Chamber	1,996	WoS
	587	USPTO
Photoelectric Effect	2,332	WoS
Uncertainty Principle	8,676	WoS
Atomic Collision	10,356	WoS
Game Theory	13,902	WoS
	702	USPTO
Doppler Effect	23,819	WoS

### Simulated Neuron Activity

The simulated neuron data is based on a realistic simulator of real neurons and a model of calcium fluorescence recording. The data used in the simulation was based on the ChaLearn Connectomics Challenges (http://connectomics.chalearn.org/). The data used was generated by a simulator described in [41] and detailed in the Methods Section. The motivation behind the simulation is to be able to isolate a predetermined number of neurons and allow them to interact in a “square petri dish”. While creating the option of isolating neurons and supplying the correct input is difficult due their connectivity to other neurons, the simulation allows tools to provide a controlled environment closely resembling real neurons. The simulation is based on a network of neurons interacting with one another. Each neuron spike is based on input received from nearby connected neurons. In the case of real neuron simulation, the spike or firing of a neuron depends on the chemical / electrical signal received from nearby connected neurons. In the case of research activity, the signal is based on information transfer between topics of research representing a neuron node. The visual aspects of the simulation are based on imaging the calcium florescence emitted by the interaction between neurons. The simulation is built on spiking based on interaction between neurons instead of separate neurons spiking at random times independently. The simulation also considers light effects based on multiple nearby neurons or light simulation based on multiple emitting sources.

The data used for the neuron activity simulation included files from the ChaLearn Connectomics Challenges (https://www.kaggle.com/c/connectomics/data). Two types of files were used. The first type of files describes the neural fluorescence activity. These files represent a time series of each neuron activity every 20ms. The neurons are ordered by rows and columns. The second type of files includes a list of X and Y position of each neuron in the “square petri dish”. The simulation represents neurons in an area of 1mm^2^. The files used for simulation included small networks of 100 neurons and normal networks of 1,000 neurons. In addition, a high rate of spiking neurons and a normal rate of spiking neurons with low signal noise were also analyzed.

### Research Topic Activity

For the analysis of research records, the time series activity used was every year. The comparison was made using the same number of top 100 or 1,000 keywords in each of the topics. The location of each of the keywords was based on the neuron positions in the “square petri dish” and was randomly picked from the existing set of neuron positions appearing in the file.

To extract publications in medicine and physics, each of the topics appearing in Table 1 was used in the Web of Science, PubMed, or USPTO. All of the records were used for each topic. For each topic all possible keywords describing the publications were counted each year. For every pair of keywords that appeared at least once in the same publication, the correlation was analyzed over the whole time period. The keywords were organized in descending order of correlation followed by number of appearances. To compare research topic activity to neuron simulation, the top 100 or 1000 ranking keyword correlations were used. The simulated neuron activity data and the research topic activity data can be found in the Supporting Information [S1–S9 Files].

### Image Comparison

Earth Mover's Distance (EMD) is used for image similarity comparison. EMD is a true metric if the ground distance is metric and if the total weights of two signatures are equal. This allows use of image spaces with a metric structure. EMD matches perceptual similarity better than other measures, when the ground distance is perceptually meaningful. This was shown by [44] for color- and texture-based image retrieval.

## Results

**Fig 2:**
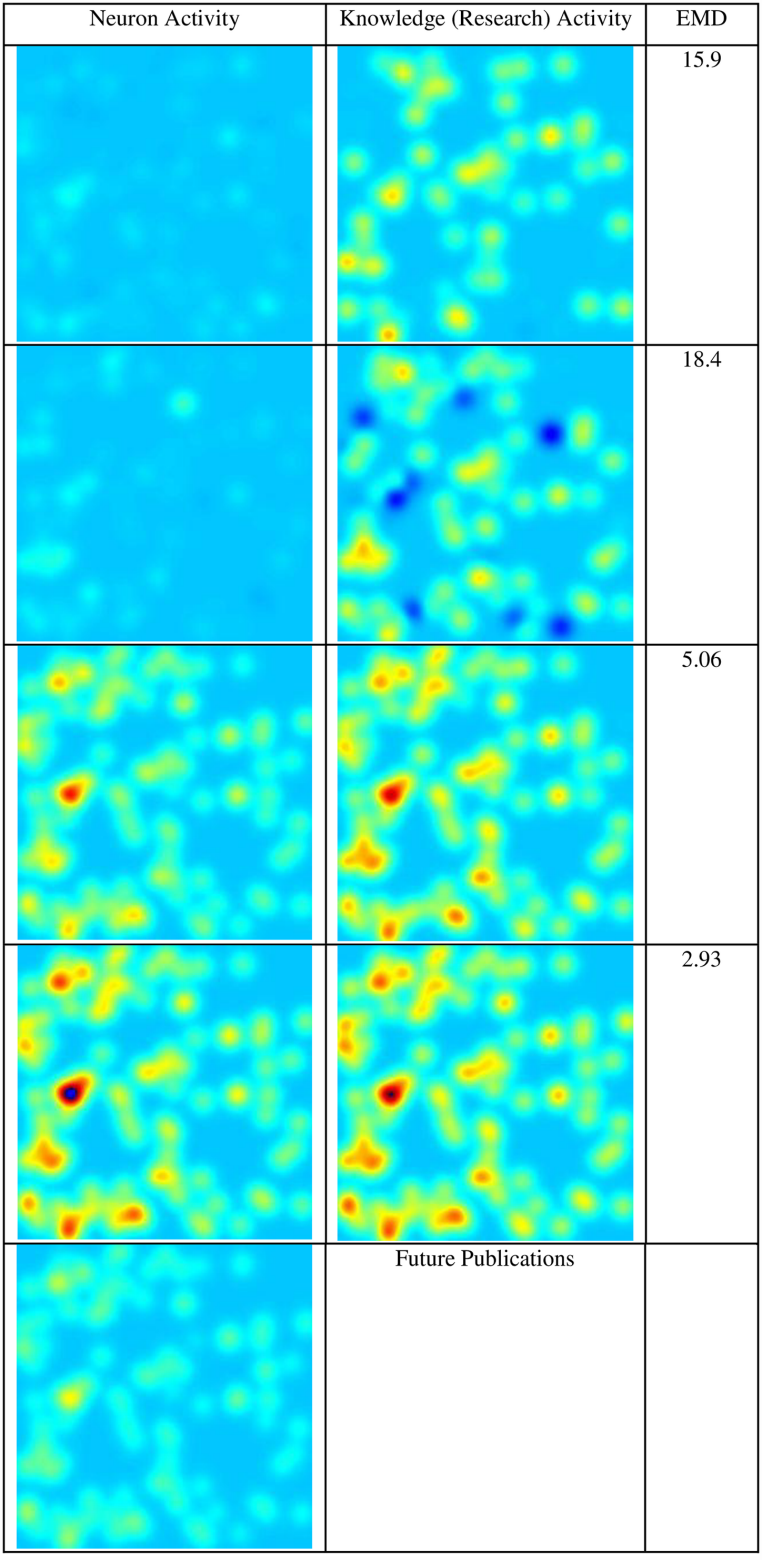
Neuron Activity vs. Research Activity on Anaphylaxis (100 nodes).

Fig 2 displays neuron activity change versus research activity on the topic of *anaphylaxis*. In this case, 100 neuron activity nodes were selected and compared to 100 high ranking correlated activity topics. The locations of the nodes of the research activity topics were randomly selected from the set of existing locations of the neuron nodes. The time line compares peaks in activity and the time scale is obviously not identical since the neuron activity is sampled at 20ms intervals and the publication activity in years. The neurons span a 1mm^2^ square area. The activity was calibrated to show similar color shades for maximum and minimum activity. The EMD indicates the size of difference between each of the two images.

When the activity peaks, the similarity between the neuron activity and the research activity becomes visible. The similarity extends to both location and intensity of a specific node area. Prior to the peak, the activity seems considerably different and the neurons are not very active. The peak appears and disappears suddenly within a few seconds of neuron brain activity and similarly within a few years of publication. The assumption of continued similarity of activity in the future makes future extrapolations possible. Analysis of the peak activity and view of the next “frame” of the movie in neuron activity might provide some information on the future decrease in activity in a research topic such as *anaphylaxis*.

**Fig 3:**
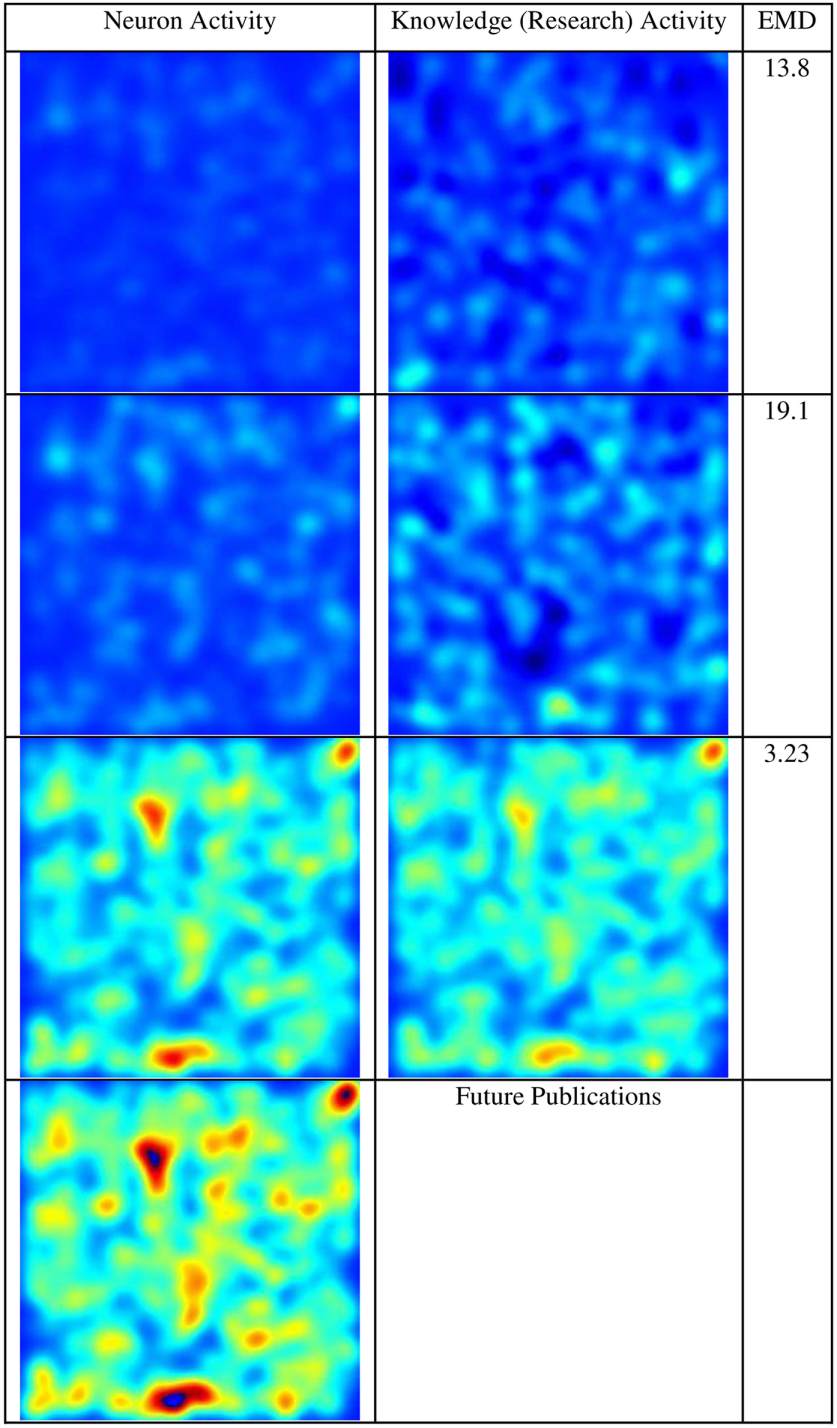
Neuron Activity vs. Research Activity on In Vitro Fertilization (1000 nodes).

Fig 3 presents a more detailed perspective using 1000 nodes of neuron activity versus research activity on the topic of *in vitro fertilization*. Comparison of the results shows again that, as the activity increases, the similarity between the communication of the nodes becomes more visible. However, assuming the activity similarity will continue, the peak of research in the area of *in vitro fertilization* has not yet been achieved, as seen in the last neuron activity frame.

**Fig 4:**
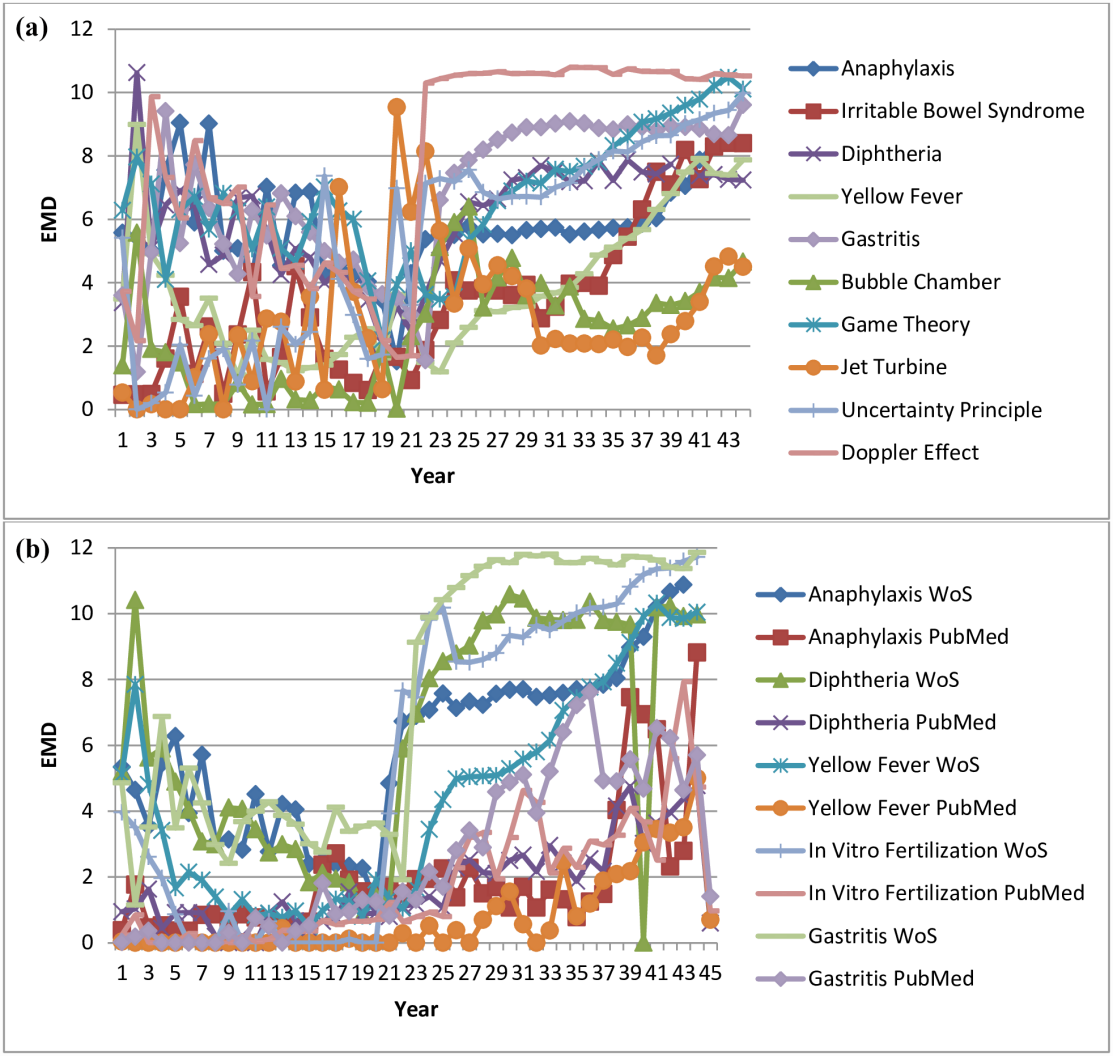
(a) Earth Moving Distance in the Domains of Medicine and Physics (b) Earth Moving Distance in Different Data Sets.

Fig 4(a) compares medical-based research activity with physics research activity over time. The medical domain includes five randomly selected topics: *anaphylaxis*, *irritable bowel syndrome*, *diphtheria*, *yellow fever*, and *gastritis*. Similarly the physics domain includes four topics: *bubble chamber*, *jet turbine*, *uncertainty principle*, and *Doppler effect*. An additional topic of *game theory* was added to represent a topic that spreads over multiple domains, including economics, political science, biology, and computer science. The Y-axis displays the EMD value based on comparing each image activity to a baseline image representing no activity, where higher values represent more research activity, while the X-axis represents the change in years from 1970.

The results show similarity between the activity of the different domains. Some topics represent a slow decrease and then fast growing research activity in areas such as *Doppler effect*, *yellow fever*, and *diphtheria*. Other topics show increased interest over time, such as *irritable bowel syndrome* and *uncertainty principle*. *Game theory*, representing a collection of research disciplines, is portrayed as a more stable research activity. Overall, no difference between the research domain activities and research domain areas is visible.

Fig 4(b) compares using EMD on two data sources, Web of Science and PubMed, to no activity baseline image based on the previous five medical topics. The sudden peaks in topics such as *anaphylaxis* and *diphtheria* can be attributed to the response to concerns about vaccine safety at the end of the 1980s. The Web of Science data source shows much more research activity than PubMed although topics are related to medical terms. This could be explained by the number of terms, or keywords, associated with each article. Web of Science has considerably fewer terms associated with each article, making each term more unique and making it easier to identify increase in specific research activity. This shows that different data sets of “similar knowledge” can be interpreted differently.

**Fig 5:**
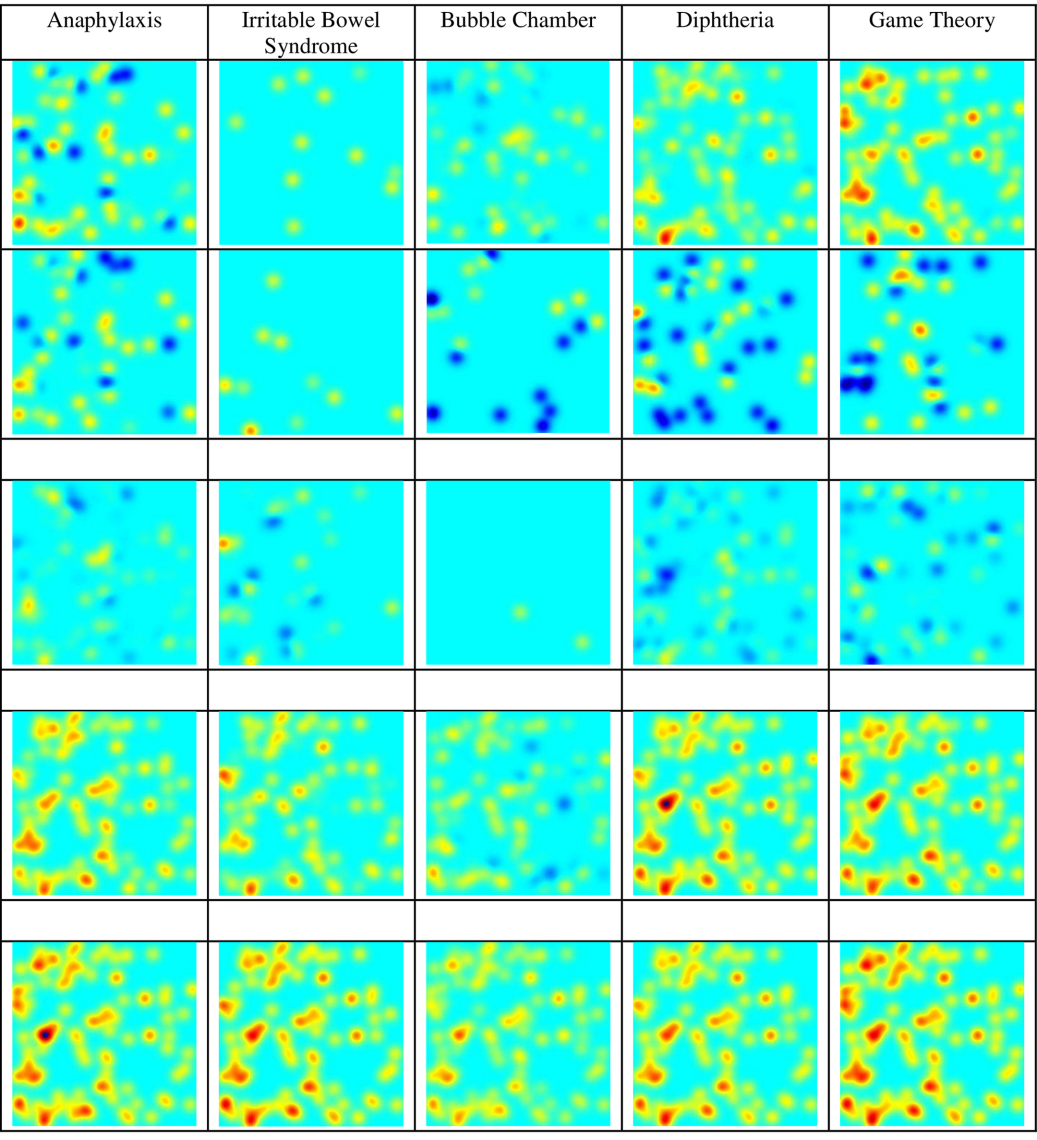
Activity of Different Topics on a Similar Timeline.

Fig 5 presents activity of *anaphylaxis*, *irritable bowel syndrome*, *bubble chamber*, *diphtheria*, and *game theory* research topics on a similar timeline. The small empty frames represent the time gap between the research activities analyzed over a period of 45 years. Although all research topics seem to be currently active, *diphtheria* displays decreased researched activity compared to previous years. *Game theory* seems to display a comeback from the early 70s, when in the 80s and 90s it seems to have “departed” from research activity. Many of the topics display a cycle of recurring peaking sudden research activity which also resembles the neuron activity.

**Fig 6:**
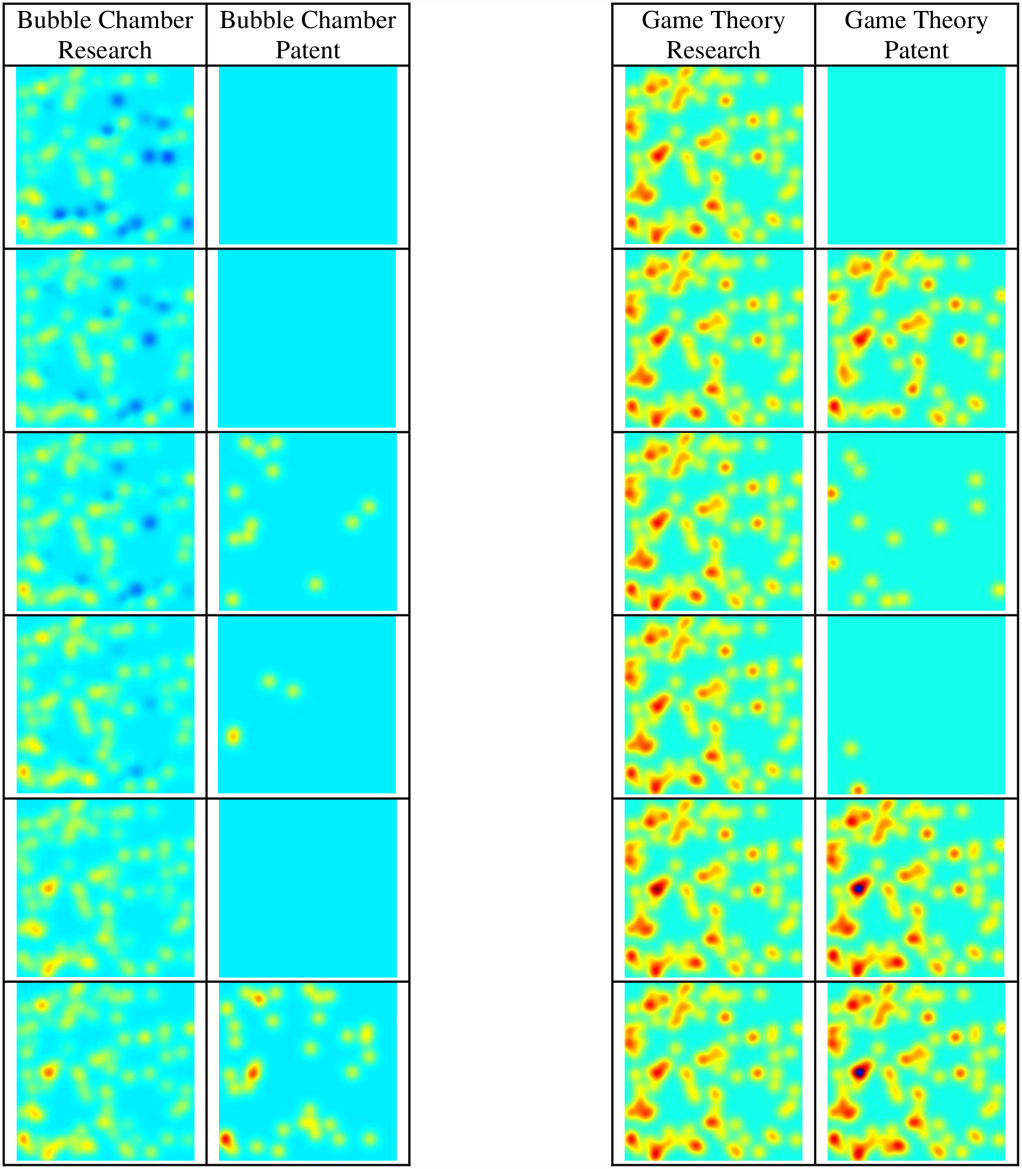
Research versus Patent Activity on a Similar Timeline.

Patents were analyzed to examine whether knowledge activity similar to neuron behavior also appears in other areas. The results show that knowledge processing activity similar to neuron behavior can be seen in patents as well as in research publications. Fig 6 displays similarity between article research activity and patent activity on the topics of *bubble chamber* and *game theory*. The activity of the two topics is presented in two timelines. The results show that for a long duration there is no activity in patents while the article research publication is active. In both topics there appears a small fluctuation in the patents, shortly followed by a sharp peak of activity in the patents, which becomes more active than the article research activity in that time duration.

The results show that neuron-like knowledge activity is not limited only to the area of article research and can be viewed also in patents. The research activity based on articles can be viewed as representing expanding knowledge, while the patents activity can be viewed as representing business activity or possibility of expanding economic activity in the topic field. One perspective is viewing the activity peak as knowledge transfer from research to patents for a short duration. Another perspective is that in comparison to neuron activity the patents also display a sudden short peak or excitation of activity due to an external stimulator, the article research activity. In both perspectives the result is similar to neuron activity—long durations of very little to no activity followed by short durations of extreme activity.

## Discussion

The goal of the paper is to show similarity between the knowledge activities. Based on this similarity it might be possible to view knowledge as independent of neurons. The minimum short term similarity presented is a year. Longer similarity, and possible knowledge explosion, can be analyzed based on rising activities detected in previous cases of similarity between knowledge publication topic activity and neuron activity. Pairs of similarities can be generated and stored based on a large number of cases. When a new topic is analyzed, it can be compared to previously known pairs and the case with highest similarity can be identified. Furthermore, groups of topics can be analyzed together to check if a combination of them yields a higher chance of knowledge explosion.

The method is used based on past knowledge activities. However, full cycles of neuron activities can be analyzed and compared showing peaks and lower points of the topic activity. New keywords are hard to simulate since the content of the possible label of keywords is harder to extract. However, previous work suggested that analyzing papers by clusters of topics can assist in identifying possible labels of new clusters [45].

Words are currently the most common method of storing knowledge. The keywords analyzed in this work can be viewed as concepts representing the main issues researched in each of the topics. Other tools of storing knowledge can be considered such as voice, music, or movies. In these cases the relations between each of the instances would have to be defined.

We show knowledge-related activity by observing similarity in behavior of brain neurons and research publication activity. This similarity is viewed as peaks in communication between neuron cells or alternatively communication between research topic areas. The results may allow us to examine knowledge according to neuron behavior patterns and to infer future knowledge behavior according to these patterns in different fields such as research, social behavior, or natural biological systems. In addition, the behavior of knowledge systems may allow us to infer neuron behavior.

## Methods

### Publication Correlation Analysis

To analyze correlation between publication topics over time, we employed the Pearson product-moment correlation coefficient. The Pearson correlation analyzes dependence between topic nodes, when dependence can be viewed as activity between neurons. The correlation is obtained by dividing the covariance of the two variables by the product of their standard deviation.

The correlation coefficient ρ_X,Y_ between two random variables X and Y with expected value μ_X_ and μ_Y_ and standard deviation σ_X_ and σ_Y_ is defined as:

$$\rho_{\text{X},\text{Y}}=\text{corr}\left(\text{X},\text{Y}\right)=\frac{\text{cov}\left(\text{X},\text{Y}\right)}{\sigma_{\text{X}}\sigma_{\text{Y}}}=\frac{\text{E}\left[\left(\text{X}-\mu_{\text{X}}\right)\left(\text{Y}-\mu_{\text{Y}}\right)\right]}{\sigma_{\text{X}}\sigma_{\text{Y}}}$$

where E is the expected value operator, cov means covariance, and corr for the correlation coefficient. The correlation coefficient is symmetric.

The Pearson correlation reaches a maximum of 1 in the case of a perfect direct (increasing) linear relationship (correlation) and a minimum of −1 in the case of a perfect decreasing (inverse) linear relationship (anticorrelation) [46]. All other values between −1 and 1 indicate the linear dependence degree between the variables. The closer the values are to 0, the less correlated the variables are, which can be viewed as having less relationship between them. A Pearson correlation coefficient of 0 means the variables are totally independent.

For a given series of n measured variables of X and Y, x_i_ and y_i_ where i = 1, 2, …, n, the sample correlation coefficient can represent the population Pearson correlation r between X and Y.

The sample correlation coefficient is defined as:

$${\text{r}}_{\text{xy}}=\frac{\sum_{\text{i}=1}^{\text{n}}\left({\text{x}}_{\text{i}}-\bar{\text{x}}\right)\left({\text{y}}_{\text{i}}-\bar{\text{y}}\right)}{\left(\text{n}-1\right){\text{S}}_{\text{x}}{\text{S}}_{\text{y}}}=\frac{\sum_{\text{i}=1}^{\text{n}}\left({\text{x}}_{\text{i}}-\bar{\text{x}}\right)\left({\text{y}}_{\text{i}}-\bar{\text{y}}\right)}{\sqrt{\sum_{\text{i}=1}^{\text{n}}{\left({\text{x}}_{\text{i}}-\bar{\text{x}}\right)}^{2}{\left({\text{y}}_{\text{i}}-\bar{\text{y}}\right)}^{2}}}$$

where x and y are the sample means of X and Y, and S_x_ and S_y_ are the standard deviations of X and Y.

### Simulation of Neural Network Activity

The changing activity of the neuron networks was generated using the NEST simulator [42, 43].

The neurons simulation was modeled as leaky integrate-and-fire neurons. The membrane potential *V*_*i*_(*t*) of a neuron *i* defined by:

$$\tau_{m}\frac{dV_{i}\left(t\right)}{dt}=-V_{i}+\frac{I_{syn}\left(t\right)}{g_{l}}$$

where g_1_ = 50pS is the leak conductance and *τ*_*m*_ = 20ms is the membrane time-constant. The time-dependent input current that derives from recurrent synaptic connections is defined by *I*_*syn*_.

The membrane potential was set to decrease exponentially and remain at zero when there are no synaptic inputs. Inputs from other neurons increase the membrane potential stimulation. When the membrane potential reaches a threshold of *V*_*thr*_ = 20mV an action potential is obtained, defined as neuron firing or neuron spiking. The spiking is followed by resetting the membrane voltage to zero for a time period of *t*_*ref*_ = 2ms.

The result of the membrane potential activates post-synaptic neurons connected to the spiking neuron. The total synaptic currents are defined by

$$\frac{dI_{syn}\left(t\right)}{dt}=-\frac{I_{syn}}{\tau_{s}}+\alpha_{int}\sum_{j=1}^{N}\sum_{k}A_{ji}E_{ji}\left(t\right)\delta \left(t-t_{j}^{k}-t_{d}\right)+\alpha_{ext}\sum_{l}\delta \left(t-t_{ext,i}^{l}-t_{d}\right)$$

where *A* is the adjacency matrix, and *τ*_*s*_ = 2ms represents a synaptic time constant. The outcome is described as excitatory post-synaptic potentials (EPSPs) and has a standard difference-of-exponentials time-course [47].

There is a limited amount of synaptic resources, and therefore neuron connection with synapses includes short term depression [48]. To simulate the conditions where the inhibitory transmission is fully blocked, fully excitatory networks were created. For each reoccurring input of given neuron *i*, the set $\left\{t_{j}^{k}\right\}$ represents times of spikes created by the presynaptic neuron *j*, where *t*_*d*_ represents a delay of *t*_*d*_ = 2*ms*. The synaptic weights of the recurrent connections are defined as homogeneous by *α*_*int*_. The recurrent time dependent strength *α*_*int*_*E*_*ji*_(*t*) is defined by using the network firing history as:

$$\frac{dE_{ji}\left(t\right)}{dt}=-\frac{E_{ji}}{\tau_{inact}}+UR_{ji}\sum_{k}\delta \left(t-t_{j}^{k}\right)$$

$$\frac{dR_{ji}\left(t\right)}{dt}=-\frac{1}{\tau_{rec}}\left(1-R_{ji}-E_{ji}\right)$$

where *E*_*ji*_(*t*) is the portion of neuron transmitting in what is viewed as the “effective state”, similarly *R*_*ji*_(*t*) represents the “recovered state”, while *I*_*ji*_(*t*) = 1 − *R*_*ji*_ − *E*_*ji*_ represents the “inactive state” [48, 49]. Similar to the synaptic current, the recovered state is set to *U* = 0.3 of neuron transmitters after the presynaptic action potential is reached. The portion decreases exponentially to the inactive state with values of time scale *τ*_*inact*_ = 3*ms*. The recovery time scale is set to *τ*_*rec*_ = 500*ms*.

Random neuron networks with depressing synapses generate synchronous activity of integrating and spiking neuron behavior [49]. On the other hand, all or none activity behavior is observed in cultured networks [50, 51]. The weight of the synaptic connections was set in all networks to represent a network bursting of 0.1 ± 0.01*Hz* which is viewed as a realistic bursting rate [51]. Each neuron network was created and simulated for 200 seconds of network activity to evaluate the bursting rate average with an initial value of *α*_*int*_ = 5.0*pA*. Each time it was bigger than the target bursting rate the synaptic weight *α*_*int*_ was decreased by 10% and vice versa for smaller values. Linear extrapolation was used to iteratively adjust until the result was smaller than 0.01 Hz to the target value.

### Simulating Neural Network Calcium Fluorescence Spiking Signals

Neuron spiking activity was used to model the calcium fluorescence signals and simulate the experiments of fluorescence signal measured. Based on [52], a simulation model was used which performs rapid increase of fluorescence after activation. The rapid increase is followed by a gradual decay of $\;\tau_{C_{a}}=1s$. The model includes the concentration of calcium located between the neurons that match to the fluorescence experimental values. The concentration is changed for each action potential that the neuron evokes for a time step *t* by a fixed amount of $\;A_{C_{a}}=50\mu$, which causes fast changes of the concentration defined by

$${\left[Ca^{2+}\right]}_{t}-{\left[Ca^{2+}\right]}_{t-1}=-\frac{\Delta t}{\;\tau_{C_{a}}}{\left[Ca^{2+}\right]}_{t-1}+A_{C_{a}}n_{t}$$

where *n*_*t*_ is the total number of action potentials.

For a given neuron *i* the fluorescence level *F* is reached by performing saturating static non-linearity on the calcium concentration, followed by adding noise η_*t*_ using Gaussian distribution with zero mean represented as:

$$F_{i,t}=\frac{{\left[Ca^{2+}\right]}_{t}}{{\left[Ca^{2+}\right]}_{t}+K_{d}}+\eta_{t}$$

The noise in the simulations was with a standard deviation of 0.03 and a set simulation saturation concentration of *K*_*d*_ = 300*μM*.

### Simulation of Light Scattering

The simulation included light scattering around the neuron cells based on a predefined area between them. The distance between each two neurons *i* and *j* is defined by d_*ij*_. The scattering length scale is based on the normal light deviation for such optical devices and instruments and set to λ_sc_ = 0.15mm. For a given neuron $F_{i,t}^{sc}$ the fluorescence extent is defined by:

$$F_{i,t}^{sc}=F_{i,t}+A_{sc}\sum_{j=1,j\neq i}^{N}F_{j,t}exp\left\{-{\left(d_{ij}/\lambda_{sc}\right)}^{2}\right\}$$

Where the scattering effect overall capacity in the simulation is determined by *A*_*sc*_. The value of the scaling factor is small, *A*_*sc*_ = 0.15 to represent the continuing effect on the simulation.

### Simulation of Generalized Entropy for Neuron Signal

The Transfer Entropy (*TE*) from two discrete Markov processes X and Y of order k is defined by [53]:

$$TE_{Y\to X}=\sum P\left(x_{n+1},x_{n}^{\left(k\right)},y_{n}^{\left(k\right)}\right)log\frac{P\left(x_{n+1}|x_{n}^{\left(k\right)},y_{n}^{\left(k\right)}\right)}{P\left(x_{n+1}|x_{n}^{\left(k\right)}\right)}$$

where *n* represents each time period measured, $x_{n}^{\left(k\right)}$ is a vector of size *k* with measurements of *X* in time periods *n*, *n*−1, …, *n*−*k*. For all values of $x_{n+1},x_{n}^{\left(k\right)}$ and $y_{n}^{\left(k\right)}$ the total value is calculated.

According to Kullback-Leibler divergence [54], *TE* represents the distance based on probability of the space between a neuron node transition matrix $P\left(x_{n+1}|x_{n}^{\left(k\right)}\right)$ and two neuron node transition matrices $P\left(x_{n+1}|x_{n}^{\left(k\right)},y_{n}^{\left(k\right)}\right)$. If the two transition matrices are identical, then the distance measure, *TE*, is defined as zero and vice versa. Signaling dependence of the transition dynamics of *X* on *Y* occurs only if transitions of *X* are not dependent on existing values of *Y* and are greater than zero.

To analyze directed functional connectivity between different neuron network nodes, the Transfer Entropy was applied. To isolate potential spike events, a discrete differentiation operator was used on the calcium fluorescence time series $\;F_{x,t}^{sc}$. For a neuron network node *x*,$\;x_{n}=\;F_{x,n+1}^{sc}-F_{x,n}^{sc}$. This is performed as pre-processing in order to improve the signal-to-noise ratio. The preprocessing creates for a limited number of data points more accurate probability distributions.

### Evaluating Similarity with Earth Mover's Distance

The Earth Mover’s Distance (EMD) is a method for image comparison based on measuring two signatures in color space. Each image is represented by color histograms, and the distance between the two distributions can be viewed as a given *ground distance*.

The problem can be viewed as two given distributions, the mass of earth spread in space and collection of holes in space. The EMD calculates the least amount of work needed to move the earth to cover the holes with earth. The unit of work relates to transporting a unit of earth by a unit of ground distance.

A *signature* is defined as a set of clusters or modes of a distribution. Each cluster is represented by a single point representing the cluster center and a weight representing the cluster size.

EMD can be formalized as a linear programming problem: Let $P=\{\left(p_{1},w_{p_{1}}\right),\ldots ,\left(p_{m},w_{p_{m}}\right)\}$ be the first signature with *m* clusters, where *p*_*i*_ is the cluster representative and *w*_*pi*_ is the weight of the cluster; $Q=\;\{\left(q_{1},w_{q_{1}}\right),\ldots ,\left(q_{n},w_{q_{n}}\right)\}$ the second signature with *n* clusters; and *D* = [*d*_*ij*_] the ground distance matrix where *d*_*ij*_ is the ground distance between clusters *p*_*i*_ and *q*_*j*_.

The goal is to find a flow = [*f*_*ij*_], with *f*_*ij*_ the flow between *p*_*i*_ and *q*_*j*_, that minimizes the overall cost

$$WORK\left(P,Q,F\right)=\sum_{i=1}^{m}\sum_{j=1}^{n}d_{ij}f_{ij}$$

subject to the following constraints:

$$f_{ij}\geq 0\;\;\;1\leq i\leq m,1\leq j\leq n$$

$$\sum_{j=1}^{n}f_{ij}\leq w_{p_{i}}\;\;\;1\leq i\leq m$$

$$\sum_{i=1}^{m}f_{ij}\leq w_{q_{j}}\;\;\;1\leq j\leq n$$

$$\sum_{i=1}^{m}\sum_{j=1}^{n}f_{ij}=\text{min}\left(\sum_{i=1}^{m}w_{p_{i}},\sum_{j=1}^{n}w_{q_{j}}\right)$$

The first constraint allows moving “earth” from *P* to *Q* and not the opposite way. The following two constraints restrict the amount of earth that can be moved by the clusters in *P* to their weights and the clusters in *Q* to receive no more earth than their weights. The last constraint forces the movement of the maximum amount of earth possible. This amount is defined as the *total flow*. After the transportation problem is solved, and the optimal flow *F* has been found, the earth mover's distance is defined as the work normalized by the total flow:

$$EMD\left(P,Q\right)=\frac{\sum_{i=1}^{m}\sum_{j=1}^{n}d_{ij}f_{ij}}{\sum_{i=1}^{m}\sum_{j=1}^{n}f_{ij}}$$

EMD is used to analyze the differences between the neuron brain activity and the research-related “invisible brain” activity images. The EMD is also used to compare a set of images, which can be viewed as a movie, each set representing a research topic over time.

The activities of the research topics are compared between the domain of medicine and the domain of physics according to the subject category identified by Web of Science field tags. In addition, an analysis is performed to compare research activity based on data extracted from different sources, including Web of Science subject category, PubMed registry number representing concept records, and USPTO representing patent classifications.

## Supporting Information

S1 File Input files1. (ZIP)

S2 File Input files2. (ZIP)

S3 File Input files3. (ZIP)

S4 File Input files4. (ZIP)

S5 File Movies. (ZIP)

S6 File Frames1. (ZIP)

S7 File Frames2. (ZIP)

S8 File Frames3. (ZIP)

S9 File Patents_Bubble_Chamber. (TXT)
