# Supplementary figures and images for: Invisible Brain: Knowledge in Research Works and Neuron Activity (part 2 of 6)
Source: PLoS One. 2016 Jul 20;11(7):e0158590. doi: 10.1371/journal.pone.0158590 (PMC4954711; doi:10.1371/journal.pone.0158590)

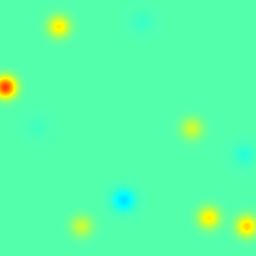

Supplement: S6 File — (ZIP) [file pone.0158590.s006.zip › anaphylaxis_/movie_WoSPubmed_anaphylaxis_Diphtheria_YellowFever_InVitroFertilization_Gastritis114.jpg]

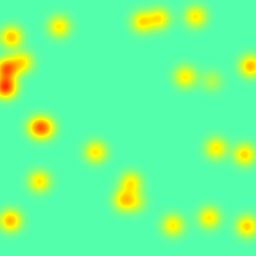

Supplement: S6 File — (ZIP) [file pone.0158590.s006.zip › anaphylaxis_/movie_WoSPubmed_anaphylaxis_Diphtheria_YellowFever_InVitroFertilization_Gastritis115.jpg]

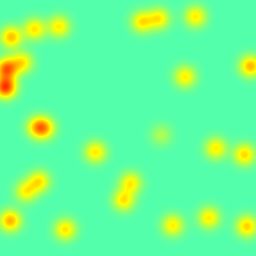

Supplement: S6 File — (ZIP) [file pone.0158590.s006.zip › anaphylaxis_/movie_WoSPubmed_anaphylaxis_Diphtheria_YellowFever_InVitroFertilization_Gastritis116.jpg]

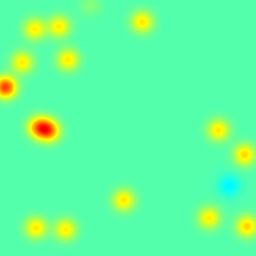

Supplement: S6 File — (ZIP) [file pone.0158590.s006.zip › anaphylaxis_/movie_WoSPubmed_anaphylaxis_Diphtheria_YellowFever_InVitroFertilization_Gastritis117.jpg]

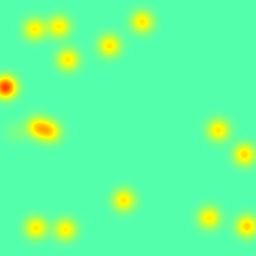

Supplement: S6 File — (ZIP) [file pone.0158590.s006.zip › anaphylaxis_/movie_WoSPubmed_anaphylaxis_Diphtheria_YellowFever_InVitroFertilization_Gastritis118.jpg]

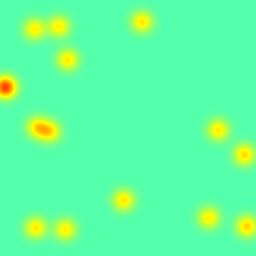

Supplement: S6 File — (ZIP) [file pone.0158590.s006.zip › anaphylaxis_/movie_WoSPubmed_anaphylaxis_Diphtheria_YellowFever_InVitroFertilization_Gastritis119.jpg]

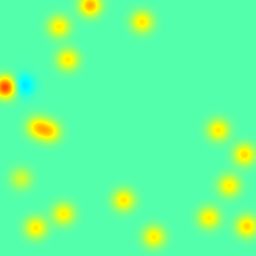

Supplement: S6 File — (ZIP) [file pone.0158590.s006.zip › anaphylaxis_/movie_WoSPubmed_anaphylaxis_Diphtheria_YellowFever_InVitroFertilization_Gastritis120.jpg]

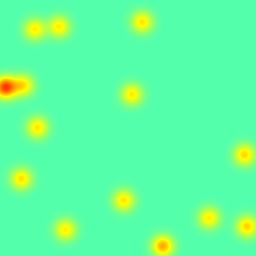

Supplement: S6 File — (ZIP) [file pone.0158590.s006.zip › anaphylaxis_/movie_WoSPubmed_anaphylaxis_Diphtheria_YellowFever_InVitroFertilization_Gastritis121.jpg]

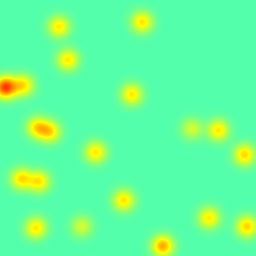

Supplement: S6 File — (ZIP) [file pone.0158590.s006.zip › anaphylaxis_/movie_WoSPubmed_anaphylaxis_Diphtheria_YellowFever_InVitroFertilization_Gastritis122.jpg]

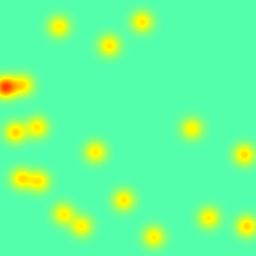

Supplement: S6 File — (ZIP) [file pone.0158590.s006.zip › anaphylaxis_/movie_WoSPubmed_anaphylaxis_Diphtheria_YellowFever_InVitroFertilization_Gastritis123.jpg]

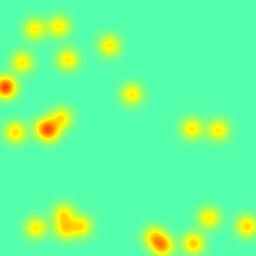

Supplement: S6 File — (ZIP) [file pone.0158590.s006.zip › anaphylaxis_/movie_WoSPubmed_anaphylaxis_Diphtheria_YellowFever_InVitroFertilization_Gastritis124.jpg]

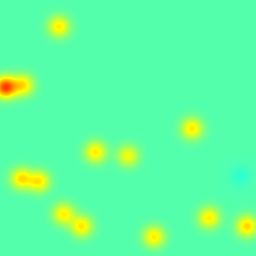

Supplement: S6 File — (ZIP) [file pone.0158590.s006.zip › anaphylaxis_/movie_WoSPubmed_anaphylaxis_Diphtheria_YellowFever_InVitroFertilization_Gastritis125.jpg]

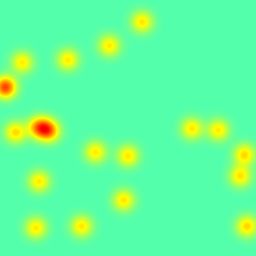

Supplement: S6 File — (ZIP) [file pone.0158590.s006.zip › anaphylaxis_/movie_WoSPubmed_anaphylaxis_Diphtheria_YellowFever_InVitroFertilization_Gastritis126.jpg]

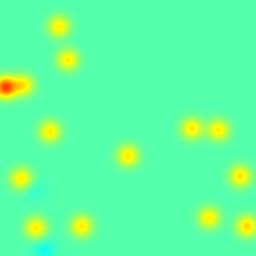

Supplement: S6 File — (ZIP) [file pone.0158590.s006.zip › anaphylaxis_/movie_WoSPubmed_anaphylaxis_Diphtheria_YellowFever_InVitroFertilization_Gastritis127.jpg]

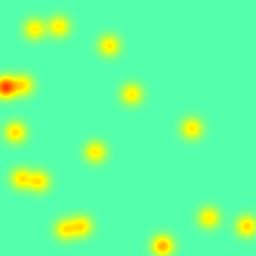

Supplement: S6 File — (ZIP) [file pone.0158590.s006.zip › anaphylaxis_/movie_WoSPubmed_anaphylaxis_Diphtheria_YellowFever_InVitroFertilization_Gastritis128.jpg]

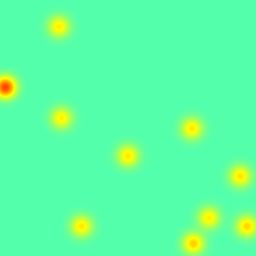

Supplement: S6 File — (ZIP) [file pone.0158590.s006.zip › anaphylaxis_/movie_WoSPubmed_anaphylaxis_Diphtheria_YellowFever_InVitroFertilization_Gastritis129.jpg]

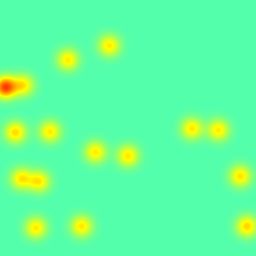

Supplement: S6 File — (ZIP) [file pone.0158590.s006.zip › anaphylaxis_/movie_WoSPubmed_anaphylaxis_Diphtheria_YellowFever_InVitroFertilization_Gastritis130.jpg]

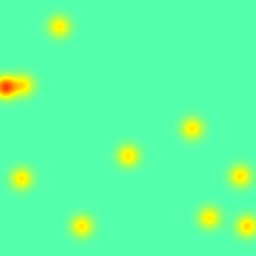

Supplement: S6 File — (ZIP) [file pone.0158590.s006.zip › anaphylaxis_/movie_WoSPubmed_anaphylaxis_Diphtheria_YellowFever_InVitroFertilization_Gastritis131.jpg]

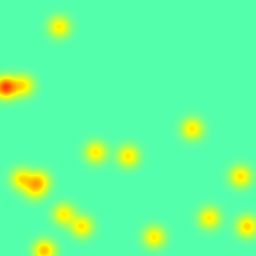

Supplement: S6 File — (ZIP) [file pone.0158590.s006.zip › anaphylaxis_/movie_WoSPubmed_anaphylaxis_Diphtheria_YellowFever_InVitroFertilization_Gastritis132.jpg]

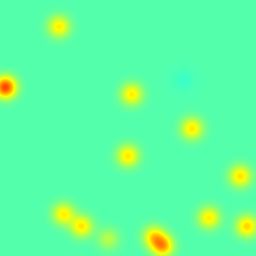

Supplement: S6 File — (ZIP) [file pone.0158590.s006.zip › anaphylaxis_/movie_WoSPubmed_anaphylaxis_Diphtheria_YellowFever_InVitroFertilization_Gastritis133.jpg]

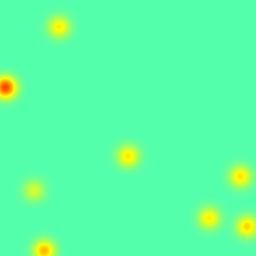

Supplement: S6 File — (ZIP) [file pone.0158590.s006.zip › anaphylaxis_/movie_WoSPubmed_anaphylaxis_Diphtheria_YellowFever_InVitroFertilization_Gastritis134.jpg]

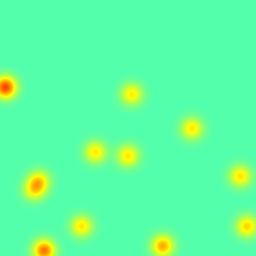

Supplement: S6 File — (ZIP) [file pone.0158590.s006.zip › anaphylaxis_/movie_WoSPubmed_anaphylaxis_Diphtheria_YellowFever_InVitroFertilization_Gastritis135.jpg]

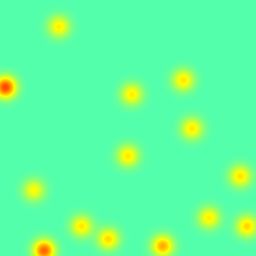

Supplement: S6 File — (ZIP) [file pone.0158590.s006.zip › anaphylaxis_/movie_WoSPubmed_anaphylaxis_Diphtheria_YellowFever_InVitroFertilization_Gastritis136.jpg]

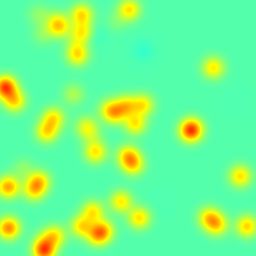

Supplement: S6 File — (ZIP) [file pone.0158590.s006.zip › anaphylaxis_/movie_WoSPubmed_anaphylaxis_Diphtheria_YellowFever_InVitroFertilization_Gastritis137.jpg]

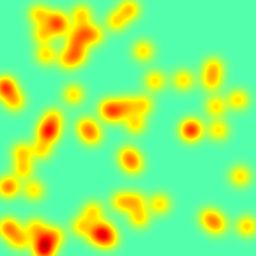

Supplement: S6 File — (ZIP) [file pone.0158590.s006.zip › anaphylaxis_/movie_WoSPubmed_anaphylaxis_Diphtheria_YellowFever_InVitroFertilization_Gastritis138.jpg]

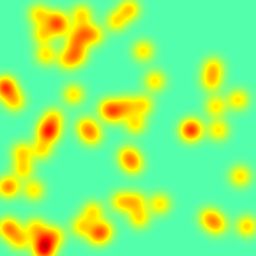

Supplement: S6 File — (ZIP) [file pone.0158590.s006.zip › anaphylaxis_/movie_WoSPubmed_anaphylaxis_Diphtheria_YellowFever_InVitroFertilization_Gastritis139.jpg]

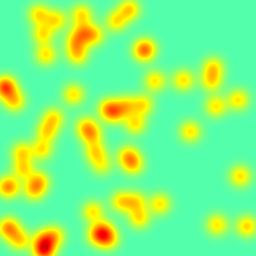

Supplement: S6 File — (ZIP) [file pone.0158590.s006.zip › anaphylaxis_/movie_WoSPubmed_anaphylaxis_Diphtheria_YellowFever_InVitroFertilization_Gastritis140.jpg]

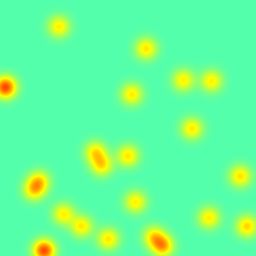

Supplement: S6 File — (ZIP) [file pone.0158590.s006.zip › anaphylaxis_/movie_WoSPubmed_anaphylaxis_Diphtheria_YellowFever_InVitroFertilization_Gastritis141.jpg]

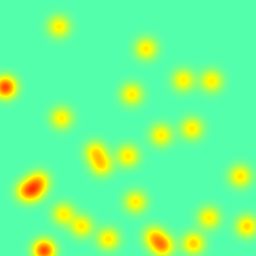

Supplement: S6 File — (ZIP) [file pone.0158590.s006.zip › anaphylaxis_/movie_WoSPubmed_anaphylaxis_Diphtheria_YellowFever_InVitroFertilization_Gastritis142.jpg]

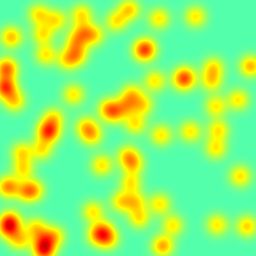

Supplement: S6 File — (ZIP) [file pone.0158590.s006.zip › anaphylaxis_/movie_WoSPubmed_anaphylaxis_Diphtheria_YellowFever_InVitroFertilization_Gastritis143.jpg]

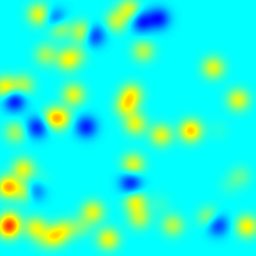

Supplement: S6 File — (ZIP) [file pone.0158590.s006.zip › anaphylaxis_765/anaphylaxis_765_01.jpg]

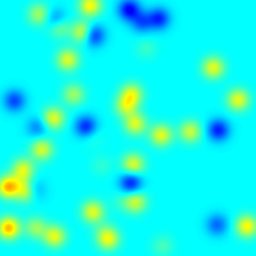

Supplement: S6 File — (ZIP) [file pone.0158590.s006.zip › anaphylaxis_765/anaphylaxis_765_02.jpg]

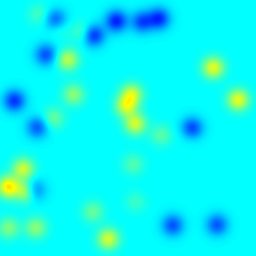

Supplement: S6 File — (ZIP) [file pone.0158590.s006.zip › anaphylaxis_765/anaphylaxis_765_03.jpg]

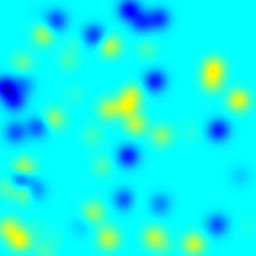

Supplement: S6 File — (ZIP) [file pone.0158590.s006.zip › anaphylaxis_765/anaphylaxis_765_04.jpg]

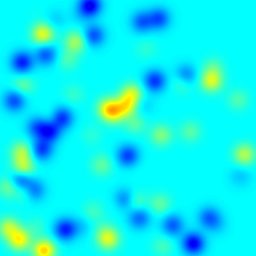

Supplement: S6 File — (ZIP) [file pone.0158590.s006.zip › anaphylaxis_765/anaphylaxis_765_05.jpg]

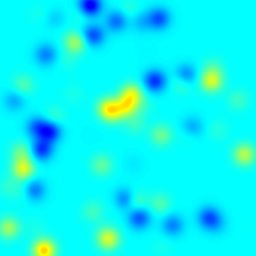

Supplement: S6 File — (ZIP) [file pone.0158590.s006.zip › anaphylaxis_765/anaphylaxis_765_06.jpg]

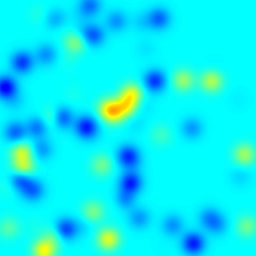

Supplement: S6 File — (ZIP) [file pone.0158590.s006.zip › anaphylaxis_765/anaphylaxis_765_07.jpg]

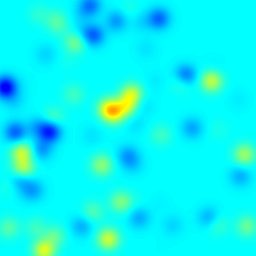

Supplement: S6 File — (ZIP) [file pone.0158590.s006.zip › anaphylaxis_765/anaphylaxis_765_08.jpg]

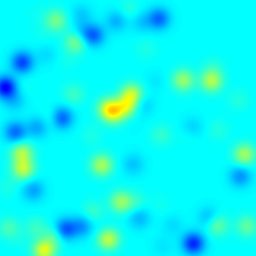

Supplement: S6 File — (ZIP) [file pone.0158590.s006.zip › anaphylaxis_765/anaphylaxis_765_09.jpg]

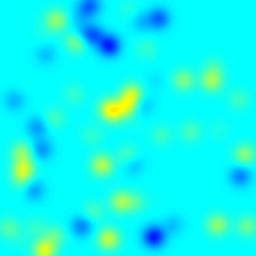

Supplement: S6 File — (ZIP) [file pone.0158590.s006.zip › anaphylaxis_765/anaphylaxis_765_10.jpg]

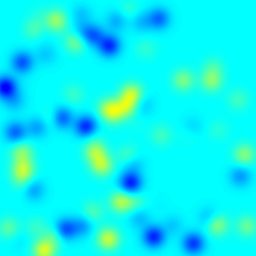

Supplement: S6 File — (ZIP) [file pone.0158590.s006.zip › anaphylaxis_765/anaphylaxis_765_11.jpg]

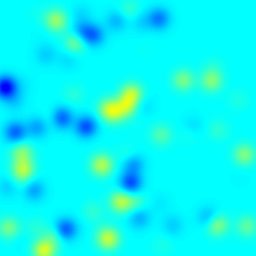

Supplement: S6 File — (ZIP) [file pone.0158590.s006.zip › anaphylaxis_765/anaphylaxis_765_12.jpg]

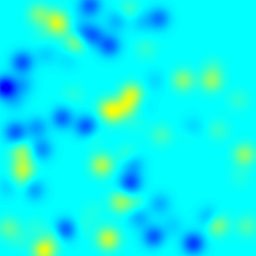

Supplement: S6 File — (ZIP) [file pone.0158590.s006.zip › anaphylaxis_765/anaphylaxis_765_13.jpg]

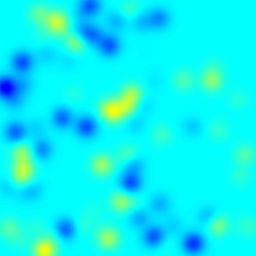

Supplement: S6 File — (ZIP) [file pone.0158590.s006.zip › anaphylaxis_765/anaphylaxis_765_14.jpg]

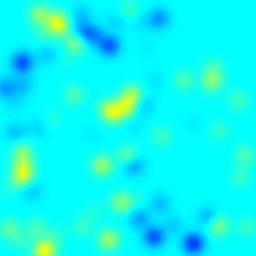

Supplement: S6 File — (ZIP) [file pone.0158590.s006.zip › anaphylaxis_765/anaphylaxis_765_15.jpg]

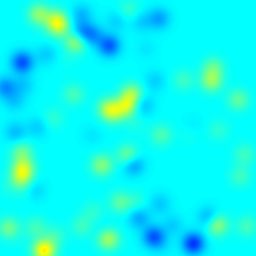

Supplement: S6 File — (ZIP) [file pone.0158590.s006.zip › anaphylaxis_765/anaphylaxis_765_16.jpg]

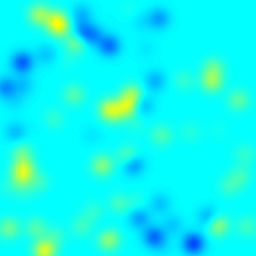

Supplement: S6 File — (ZIP) [file pone.0158590.s006.zip › anaphylaxis_765/anaphylaxis_765_17.jpg]

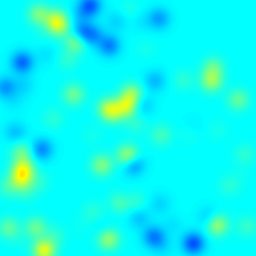

Supplement: S6 File — (ZIP) [file pone.0158590.s006.zip › anaphylaxis_765/anaphylaxis_765_18.jpg]

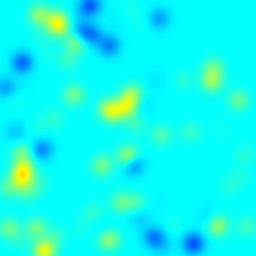

Supplement: S6 File — (ZIP) [file pone.0158590.s006.zip › anaphylaxis_765/anaphylaxis_765_19.jpg]

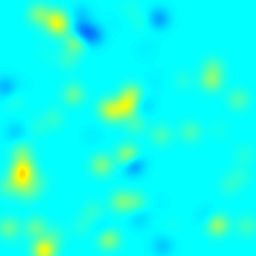

Supplement: S6 File — (ZIP) [file pone.0158590.s006.zip › anaphylaxis_765/anaphylaxis_765_20.jpg]

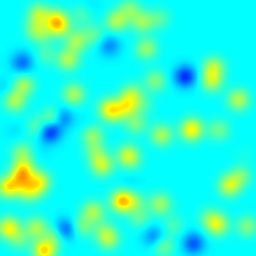

Supplement: S6 File — (ZIP) [file pone.0158590.s006.zip › anaphylaxis_765/anaphylaxis_765_21.jpg]

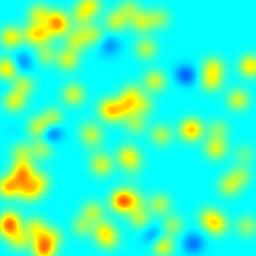

Supplement: S6 File — (ZIP) [file pone.0158590.s006.zip › anaphylaxis_765/anaphylaxis_765_22.jpg]

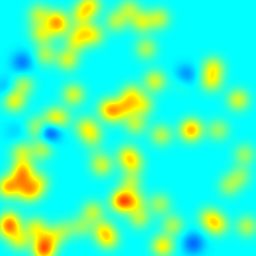

Supplement: S6 File — (ZIP) [file pone.0158590.s006.zip › anaphylaxis_765/anaphylaxis_765_23.jpg]

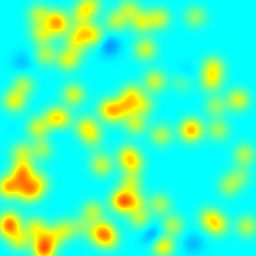

Supplement: S6 File — (ZIP) [file pone.0158590.s006.zip › anaphylaxis_765/anaphylaxis_765_24.jpg]

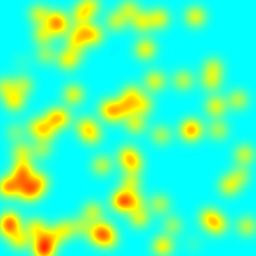

Supplement: S6 File — (ZIP) [file pone.0158590.s006.zip › anaphylaxis_765/anaphylaxis_765_25.jpg]

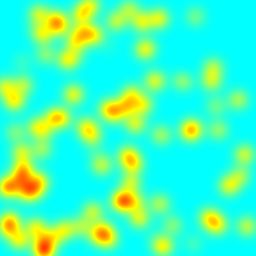

Supplement: S6 File — (ZIP) [file pone.0158590.s006.zip › anaphylaxis_765/anaphylaxis_765_26.jpg]

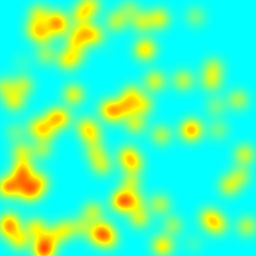

Supplement: S6 File — (ZIP) [file pone.0158590.s006.zip › anaphylaxis_765/anaphylaxis_765_27.jpg]

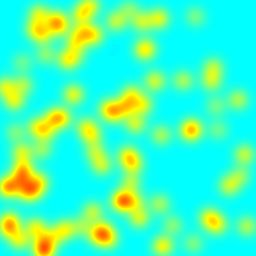

Supplement: S6 File — (ZIP) [file pone.0158590.s006.zip › anaphylaxis_765/anaphylaxis_765_28.jpg]

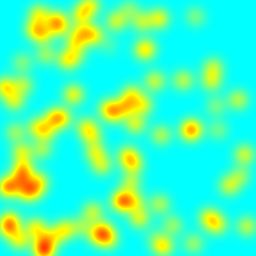

Supplement: S6 File — (ZIP) [file pone.0158590.s006.zip › anaphylaxis_765/anaphylaxis_765_29.jpg]

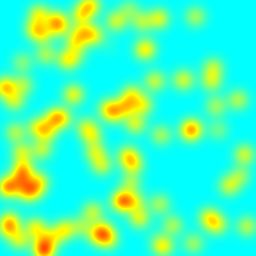

Supplement: S6 File — (ZIP) [file pone.0158590.s006.zip › anaphylaxis_765/anaphylaxis_765_30.jpg]

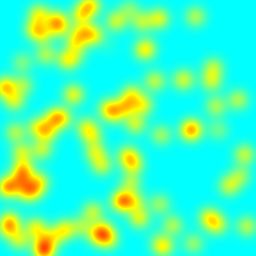

Supplement: S6 File — (ZIP) [file pone.0158590.s006.zip › anaphylaxis_765/anaphylaxis_765_31.jpg]

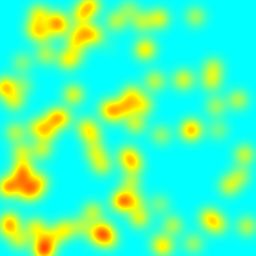

Supplement: S6 File — (ZIP) [file pone.0158590.s006.zip › anaphylaxis_765/anaphylaxis_765_32.jpg]

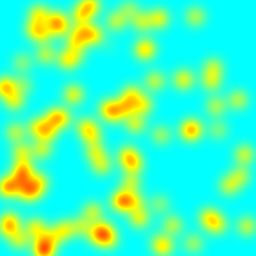

Supplement: S6 File — (ZIP) [file pone.0158590.s006.zip › anaphylaxis_765/anaphylaxis_765_33.jpg]

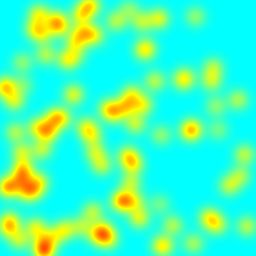

Supplement: S6 File — (ZIP) [file pone.0158590.s006.zip › anaphylaxis_765/anaphylaxis_765_34.jpg]

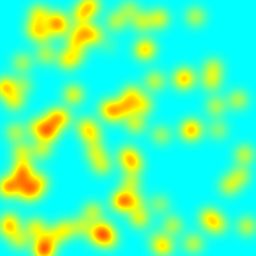

Supplement: S6 File — (ZIP) [file pone.0158590.s006.zip › anaphylaxis_765/anaphylaxis_765_35.jpg]

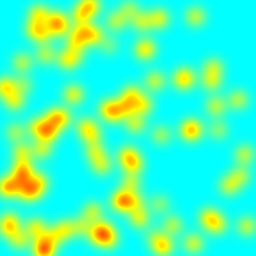

Supplement: S6 File — (ZIP) [file pone.0158590.s006.zip › anaphylaxis_765/anaphylaxis_765_36.jpg]

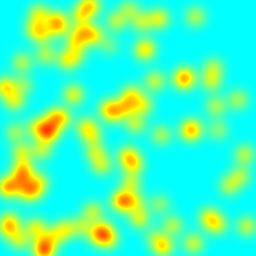

Supplement: S6 File — (ZIP) [file pone.0158590.s006.zip › anaphylaxis_765/anaphylaxis_765_37.jpg]

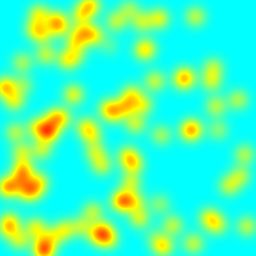

Supplement: S6 File — (ZIP) [file pone.0158590.s006.zip › anaphylaxis_765/anaphylaxis_765_38.jpg]

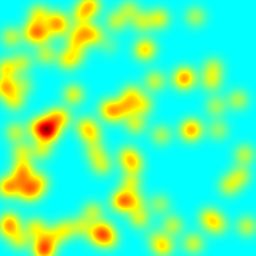

Supplement: S6 File — (ZIP) [file pone.0158590.s006.zip › anaphylaxis_765/anaphylaxis_765_39.jpg]

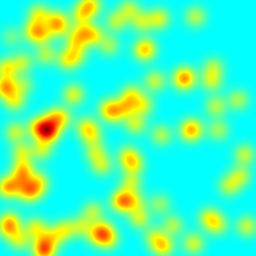

Supplement: S6 File — (ZIP) [file pone.0158590.s006.zip › anaphylaxis_765/anaphylaxis_765_40.jpg]

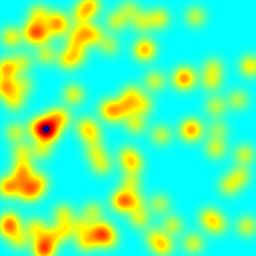

Supplement: S6 File — (ZIP) [file pone.0158590.s006.zip › anaphylaxis_765/anaphylaxis_765_41.jpg]

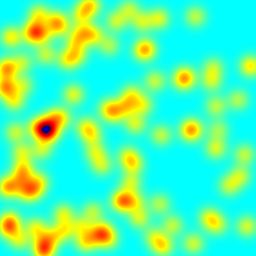

Supplement: S6 File — (ZIP) [file pone.0158590.s006.zip › anaphylaxis_765/anaphylaxis_765_42.jpg]

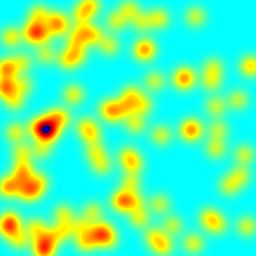

Supplement: S6 File — (ZIP) [file pone.0158590.s006.zip › anaphylaxis_765/anaphylaxis_765_43.jpg]

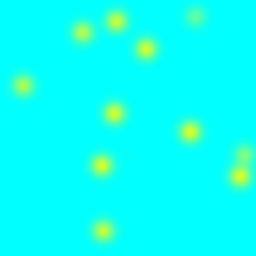

Supplement: S6 File — (ZIP) [file pone.0158590.s006.zip › anaphylaxis_765_IrritableBowelSyndrome_866_BubbleChamber_435_Diphtheria_735_GameTheory_9285/anaphylaxis_765_IrritableBowelSyndrome_866_BubbleChamber_435_Diphtheria_735_GameTheory_9285100.jpg]

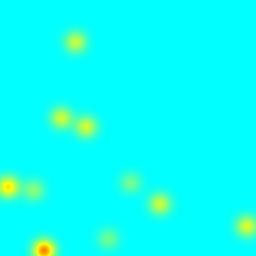

Supplement: S6 File — (ZIP) [file pone.0158590.s006.zip › anaphylaxis_765_IrritableBowelSyndrome_866_BubbleChamber_435_Diphtheria_735_GameTheory_9285/anaphylaxis_765_IrritableBowelSyndrome_866_BubbleChamber_435_Diphtheria_735_GameTheory_9285101.jpg]

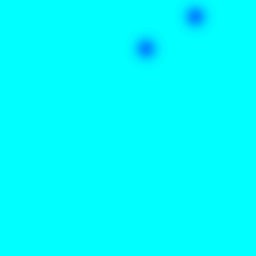

Supplement: S6 File — (ZIP) [file pone.0158590.s006.zip › anaphylaxis_765_IrritableBowelSyndrome_866_BubbleChamber_435_Diphtheria_735_GameTheory_9285/anaphylaxis_765_IrritableBowelSyndrome_866_BubbleChamber_435_Diphtheria_735_GameTheory_9285102.jpg]

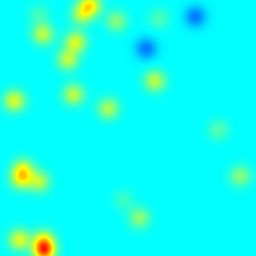

Supplement: S6 File — (ZIP) [file pone.0158590.s006.zip › anaphylaxis_765_IrritableBowelSyndrome_866_BubbleChamber_435_Diphtheria_735_GameTheory_9285/anaphylaxis_765_IrritableBowelSyndrome_866_BubbleChamber_435_Diphtheria_735_GameTheory_9285103.jpg]

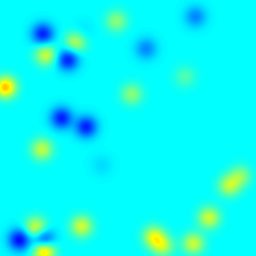

Supplement: S6 File — (ZIP) [file pone.0158590.s006.zip › anaphylaxis_765_IrritableBowelSyndrome_866_BubbleChamber_435_Diphtheria_735_GameTheory_9285/anaphylaxis_765_IrritableBowelSyndrome_866_BubbleChamber_435_Diphtheria_735_GameTheory_9285104.jpg]

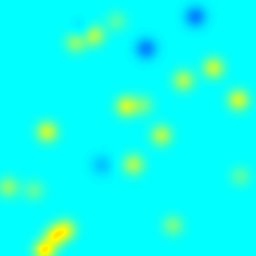

Supplement: S6 File — (ZIP) [file pone.0158590.s006.zip › anaphylaxis_765_IrritableBowelSyndrome_866_BubbleChamber_435_Diphtheria_735_GameTheory_9285/anaphylaxis_765_IrritableBowelSyndrome_866_BubbleChamber_435_Diphtheria_735_GameTheory_9285105.jpg]

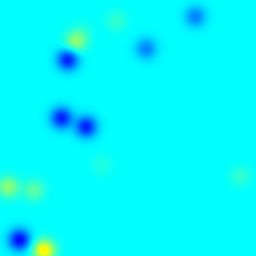

Supplement: S6 File — (ZIP) [file pone.0158590.s006.zip › anaphylaxis_765_IrritableBowelSyndrome_866_BubbleChamber_435_Diphtheria_735_GameTheory_9285/anaphylaxis_765_IrritableBowelSyndrome_866_BubbleChamber_435_Diphtheria_735_GameTheory_9285106.jpg]

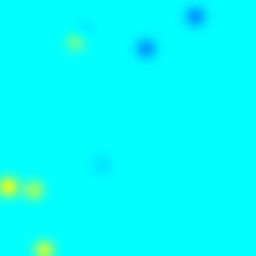

Supplement: S6 File — (ZIP) [file pone.0158590.s006.zip › anaphylaxis_765_IrritableBowelSyndrome_866_BubbleChamber_435_Diphtheria_735_GameTheory_9285/anaphylaxis_765_IrritableBowelSyndrome_866_BubbleChamber_435_Diphtheria_735_GameTheory_9285107.jpg]

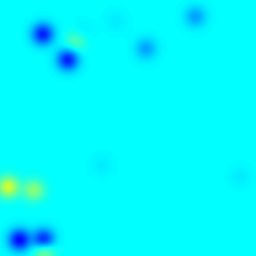

Supplement: S6 File — (ZIP) [file pone.0158590.s006.zip › anaphylaxis_765_IrritableBowelSyndrome_866_BubbleChamber_435_Diphtheria_735_GameTheory_9285/anaphylaxis_765_IrritableBowelSyndrome_866_BubbleChamber_435_Diphtheria_735_GameTheory_9285108.jpg]

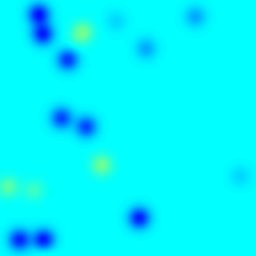

Supplement: S6 File — (ZIP) [file pone.0158590.s006.zip › anaphylaxis_765_IrritableBowelSyndrome_866_BubbleChamber_435_Diphtheria_735_GameTheory_9285/anaphylaxis_765_IrritableBowelSyndrome_866_BubbleChamber_435_Diphtheria_735_GameTheory_9285109.jpg]

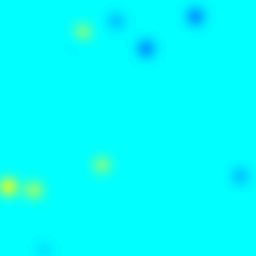

Supplement: S6 File — (ZIP) [file pone.0158590.s006.zip › anaphylaxis_765_IrritableBowelSyndrome_866_BubbleChamber_435_Diphtheria_735_GameTheory_9285/anaphylaxis_765_IrritableBowelSyndrome_866_BubbleChamber_435_Diphtheria_735_GameTheory_9285110.jpg]

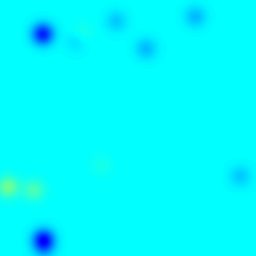

Supplement: S6 File — (ZIP) [file pone.0158590.s006.zip › anaphylaxis_765_IrritableBowelSyndrome_866_BubbleChamber_435_Diphtheria_735_GameTheory_9285/anaphylaxis_765_IrritableBowelSyndrome_866_BubbleChamber_435_Diphtheria_735_GameTheory_9285111.jpg]

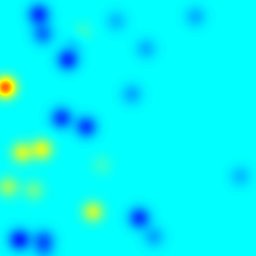

Supplement: S6 File — (ZIP) [file pone.0158590.s006.zip › anaphylaxis_765_IrritableBowelSyndrome_866_BubbleChamber_435_Diphtheria_735_GameTheory_9285/anaphylaxis_765_IrritableBowelSyndrome_866_BubbleChamber_435_Diphtheria_735_GameTheory_9285112.jpg]

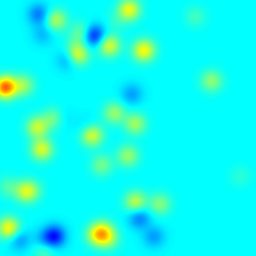

Supplement: S6 File — (ZIP) [file pone.0158590.s006.zip › anaphylaxis_765_IrritableBowelSyndrome_866_BubbleChamber_435_Diphtheria_735_GameTheory_9285/anaphylaxis_765_IrritableBowelSyndrome_866_BubbleChamber_435_Diphtheria_735_GameTheory_9285113.jpg]

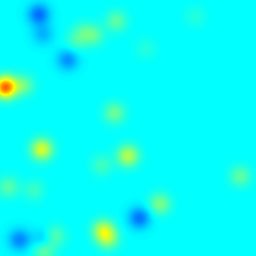

Supplement: S6 File — (ZIP) [file pone.0158590.s006.zip › anaphylaxis_765_IrritableBowelSyndrome_866_BubbleChamber_435_Diphtheria_735_GameTheory_9285/anaphylaxis_765_IrritableBowelSyndrome_866_BubbleChamber_435_Diphtheria_735_GameTheory_9285114.jpg]

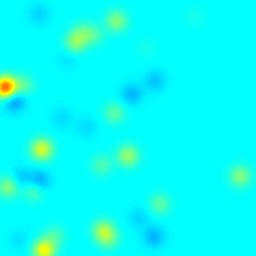

Supplement: S6 File — (ZIP) [file pone.0158590.s006.zip › anaphylaxis_765_IrritableBowelSyndrome_866_BubbleChamber_435_Diphtheria_735_GameTheory_9285/anaphylaxis_765_IrritableBowelSyndrome_866_BubbleChamber_435_Diphtheria_735_GameTheory_9285115.jpg]

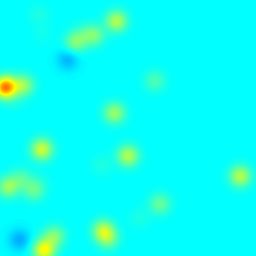

Supplement: S6 File — (ZIP) [file pone.0158590.s006.zip › anaphylaxis_765_IrritableBowelSyndrome_866_BubbleChamber_435_Diphtheria_735_GameTheory_9285/anaphylaxis_765_IrritableBowelSyndrome_866_BubbleChamber_435_Diphtheria_735_GameTheory_9285116.jpg]

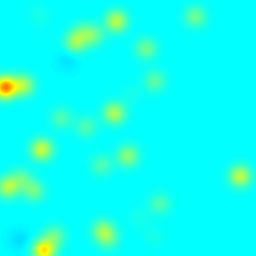

Supplement: S6 File — (ZIP) [file pone.0158590.s006.zip › anaphylaxis_765_IrritableBowelSyndrome_866_BubbleChamber_435_Diphtheria_735_GameTheory_9285/anaphylaxis_765_IrritableBowelSyndrome_866_BubbleChamber_435_Diphtheria_735_GameTheory_9285117.jpg]

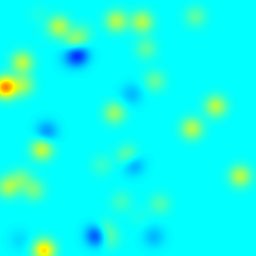

Supplement: S6 File — (ZIP) [file pone.0158590.s006.zip › anaphylaxis_765_IrritableBowelSyndrome_866_BubbleChamber_435_Diphtheria_735_GameTheory_9285/anaphylaxis_765_IrritableBowelSyndrome_866_BubbleChamber_435_Diphtheria_735_GameTheory_9285118.jpg]

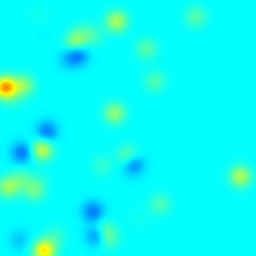

Supplement: S6 File — (ZIP) [file pone.0158590.s006.zip › anaphylaxis_765_IrritableBowelSyndrome_866_BubbleChamber_435_Diphtheria_735_GameTheory_9285/anaphylaxis_765_IrritableBowelSyndrome_866_BubbleChamber_435_Diphtheria_735_GameTheory_9285119.jpg]

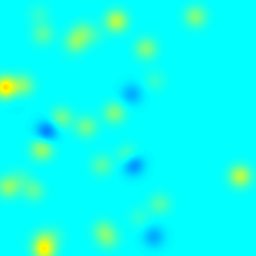

Supplement: S6 File — (ZIP) [file pone.0158590.s006.zip › anaphylaxis_765_IrritableBowelSyndrome_866_BubbleChamber_435_Diphtheria_735_GameTheory_9285/anaphylaxis_765_IrritableBowelSyndrome_866_BubbleChamber_435_Diphtheria_735_GameTheory_9285120.jpg]

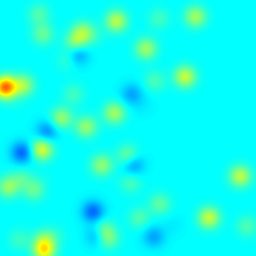

Supplement: S6 File — (ZIP) [file pone.0158590.s006.zip › anaphylaxis_765_IrritableBowelSyndrome_866_BubbleChamber_435_Diphtheria_735_GameTheory_9285/anaphylaxis_765_IrritableBowelSyndrome_866_BubbleChamber_435_Diphtheria_735_GameTheory_9285121.jpg]

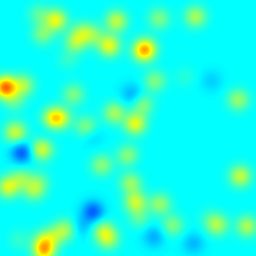

Supplement: S6 File — (ZIP) [file pone.0158590.s006.zip › anaphylaxis_765_IrritableBowelSyndrome_866_BubbleChamber_435_Diphtheria_735_GameTheory_9285/anaphylaxis_765_IrritableBowelSyndrome_866_BubbleChamber_435_Diphtheria_735_GameTheory_9285122.jpg]

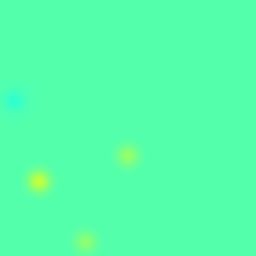

Supplement: S7 File — (ZIP) [file pone.0158590.s007.zip › Gastritis/movie_WoSPubmed_anaphylaxis_Diphtheria_YellowFever_InVitroFertilization_Gastritis900.jpg]

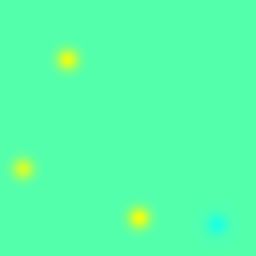

Supplement: S7 File — (ZIP) [file pone.0158590.s007.zip › Gastritis/movie_WoSPubmed_anaphylaxis_Diphtheria_YellowFever_InVitroFertilization_Gastritis901.jpg]

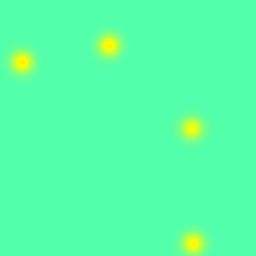

Supplement: S7 File — (ZIP) [file pone.0158590.s007.zip › Gastritis/movie_WoSPubmed_anaphylaxis_Diphtheria_YellowFever_InVitroFertilization_Gastritis902.jpg]

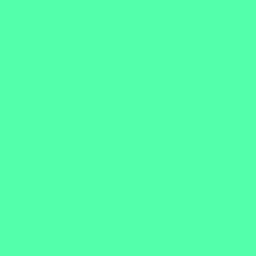

Supplement: S7 File — (ZIP) [file pone.0158590.s007.zip › Gastritis/movie_WoSPubmed_anaphylaxis_Diphtheria_YellowFever_InVitroFertilization_Gastritis903.jpg]
