# Supplementary figures and images for: Invisible Brain: Knowledge in Research Works and Neuron Activity (part 1 of 6)
Source: PLoS One. 2016 Jul 20;11(7):e0158590. doi: 10.1371/journal.pone.0158590 (PMC4954711; doi:10.1371/journal.pone.0158590)

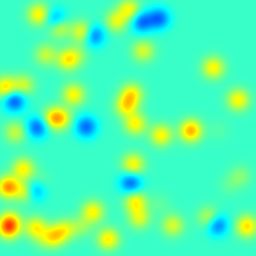

Supplement: S6 File — (ZIP) [file pone.0158590.s006.zip › anaph/movie_anaph_IrrBowSyne_BubCham_Diphth_GamThe_JetTur_UncPri_DopEff_YelFev_Gas01.jpg]

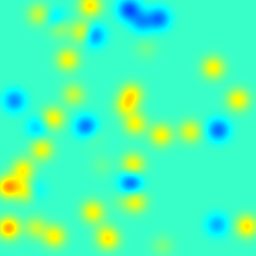

Supplement: S6 File — (ZIP) [file pone.0158590.s006.zip › anaph/movie_anaph_IrrBowSyne_BubCham_Diphth_GamThe_JetTur_UncPri_DopEff_YelFev_Gas02.jpg]

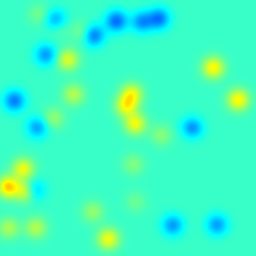

Supplement: S6 File — (ZIP) [file pone.0158590.s006.zip › anaph/movie_anaph_IrrBowSyne_BubCham_Diphth_GamThe_JetTur_UncPri_DopEff_YelFev_Gas03.jpg]

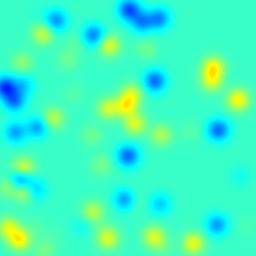

Supplement: S6 File — (ZIP) [file pone.0158590.s006.zip › anaph/movie_anaph_IrrBowSyne_BubCham_Diphth_GamThe_JetTur_UncPri_DopEff_YelFev_Gas04.jpg]

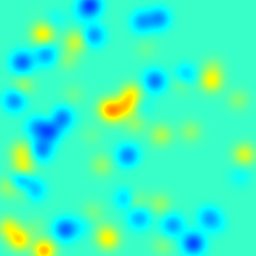

Supplement: S6 File — (ZIP) [file pone.0158590.s006.zip › anaph/movie_anaph_IrrBowSyne_BubCham_Diphth_GamThe_JetTur_UncPri_DopEff_YelFev_Gas05.jpg]

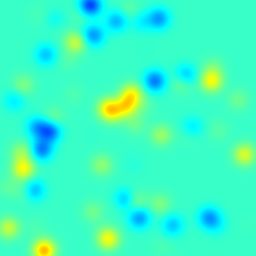

Supplement: S6 File — (ZIP) [file pone.0158590.s006.zip › anaph/movie_anaph_IrrBowSyne_BubCham_Diphth_GamThe_JetTur_UncPri_DopEff_YelFev_Gas06.jpg]

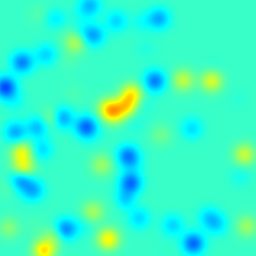

Supplement: S6 File — (ZIP) [file pone.0158590.s006.zip › anaph/movie_anaph_IrrBowSyne_BubCham_Diphth_GamThe_JetTur_UncPri_DopEff_YelFev_Gas07.jpg]

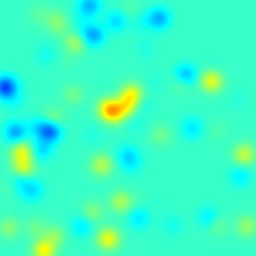

Supplement: S6 File — (ZIP) [file pone.0158590.s006.zip › anaph/movie_anaph_IrrBowSyne_BubCham_Diphth_GamThe_JetTur_UncPri_DopEff_YelFev_Gas08.jpg]

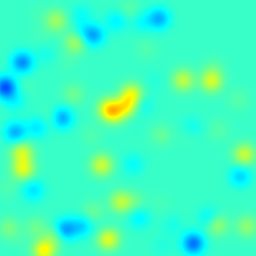

Supplement: S6 File — (ZIP) [file pone.0158590.s006.zip › anaph/movie_anaph_IrrBowSyne_BubCham_Diphth_GamThe_JetTur_UncPri_DopEff_YelFev_Gas09.jpg]

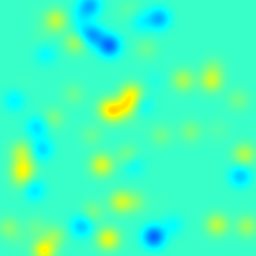

Supplement: S6 File — (ZIP) [file pone.0158590.s006.zip › anaph/movie_anaph_IrrBowSyne_BubCham_Diphth_GamThe_JetTur_UncPri_DopEff_YelFev_Gas10.jpg]

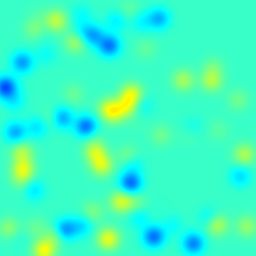

Supplement: S6 File — (ZIP) [file pone.0158590.s006.zip › anaph/movie_anaph_IrrBowSyne_BubCham_Diphth_GamThe_JetTur_UncPri_DopEff_YelFev_Gas11.jpg]

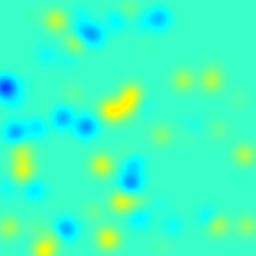

Supplement: S6 File — (ZIP) [file pone.0158590.s006.zip › anaph/movie_anaph_IrrBowSyne_BubCham_Diphth_GamThe_JetTur_UncPri_DopEff_YelFev_Gas12.jpg]

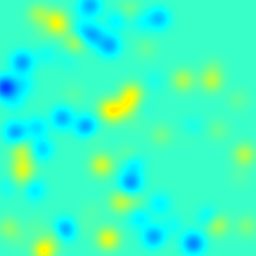

Supplement: S6 File — (ZIP) [file pone.0158590.s006.zip › anaph/movie_anaph_IrrBowSyne_BubCham_Diphth_GamThe_JetTur_UncPri_DopEff_YelFev_Gas13.jpg]

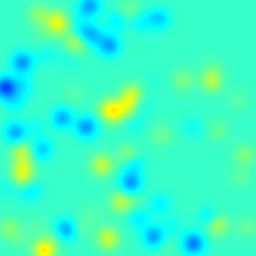

Supplement: S6 File — (ZIP) [file pone.0158590.s006.zip › anaph/movie_anaph_IrrBowSyne_BubCham_Diphth_GamThe_JetTur_UncPri_DopEff_YelFev_Gas14.jpg]

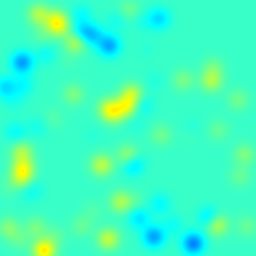

Supplement: S6 File — (ZIP) [file pone.0158590.s006.zip › anaph/movie_anaph_IrrBowSyne_BubCham_Diphth_GamThe_JetTur_UncPri_DopEff_YelFev_Gas15.jpg]

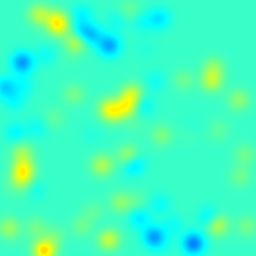

Supplement: S6 File — (ZIP) [file pone.0158590.s006.zip › anaph/movie_anaph_IrrBowSyne_BubCham_Diphth_GamThe_JetTur_UncPri_DopEff_YelFev_Gas16.jpg]

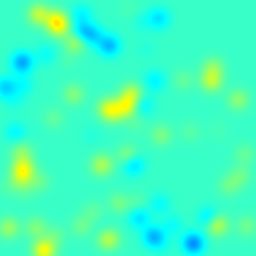

Supplement: S6 File — (ZIP) [file pone.0158590.s006.zip › anaph/movie_anaph_IrrBowSyne_BubCham_Diphth_GamThe_JetTur_UncPri_DopEff_YelFev_Gas17.jpg]

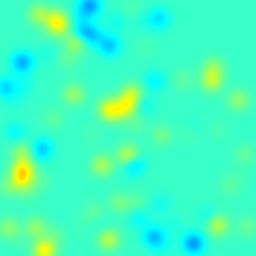

Supplement: S6 File — (ZIP) [file pone.0158590.s006.zip › anaph/movie_anaph_IrrBowSyne_BubCham_Diphth_GamThe_JetTur_UncPri_DopEff_YelFev_Gas18.jpg]

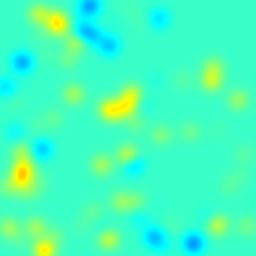

Supplement: S6 File — (ZIP) [file pone.0158590.s006.zip › anaph/movie_anaph_IrrBowSyne_BubCham_Diphth_GamThe_JetTur_UncPri_DopEff_YelFev_Gas19.jpg]

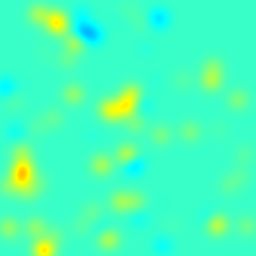

Supplement: S6 File — (ZIP) [file pone.0158590.s006.zip › anaph/movie_anaph_IrrBowSyne_BubCham_Diphth_GamThe_JetTur_UncPri_DopEff_YelFev_Gas20.jpg]

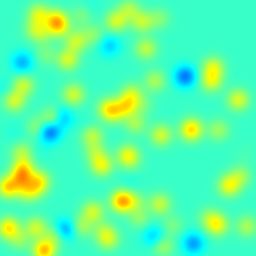

Supplement: S6 File — (ZIP) [file pone.0158590.s006.zip › anaph/movie_anaph_IrrBowSyne_BubCham_Diphth_GamThe_JetTur_UncPri_DopEff_YelFev_Gas21.jpg]

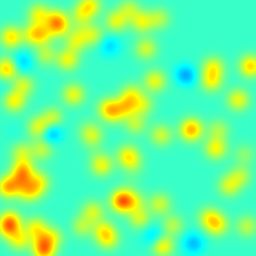

Supplement: S6 File — (ZIP) [file pone.0158590.s006.zip › anaph/movie_anaph_IrrBowSyne_BubCham_Diphth_GamThe_JetTur_UncPri_DopEff_YelFev_Gas22.jpg]

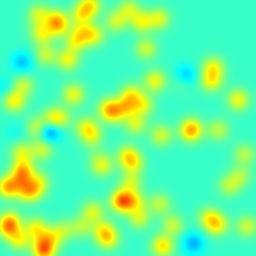

Supplement: S6 File — (ZIP) [file pone.0158590.s006.zip › anaph/movie_anaph_IrrBowSyne_BubCham_Diphth_GamThe_JetTur_UncPri_DopEff_YelFev_Gas23.jpg]

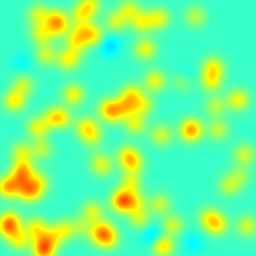

Supplement: S6 File — (ZIP) [file pone.0158590.s006.zip › anaph/movie_anaph_IrrBowSyne_BubCham_Diphth_GamThe_JetTur_UncPri_DopEff_YelFev_Gas24.jpg]

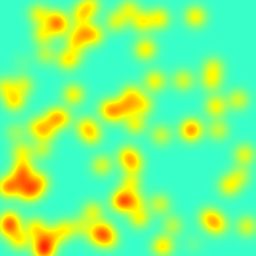

Supplement: S6 File — (ZIP) [file pone.0158590.s006.zip › anaph/movie_anaph_IrrBowSyne_BubCham_Diphth_GamThe_JetTur_UncPri_DopEff_YelFev_Gas25.jpg]

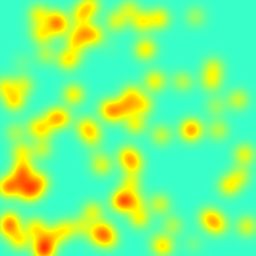

Supplement: S6 File — (ZIP) [file pone.0158590.s006.zip › anaph/movie_anaph_IrrBowSyne_BubCham_Diphth_GamThe_JetTur_UncPri_DopEff_YelFev_Gas26.jpg]

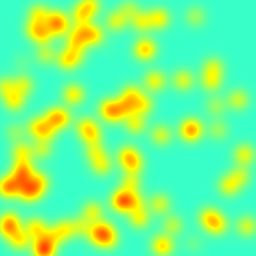

Supplement: S6 File — (ZIP) [file pone.0158590.s006.zip › anaph/movie_anaph_IrrBowSyne_BubCham_Diphth_GamThe_JetTur_UncPri_DopEff_YelFev_Gas27.jpg]

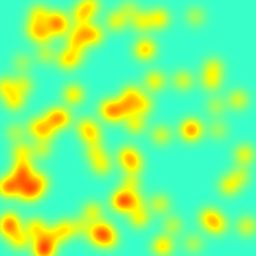

Supplement: S6 File — (ZIP) [file pone.0158590.s006.zip › anaph/movie_anaph_IrrBowSyne_BubCham_Diphth_GamThe_JetTur_UncPri_DopEff_YelFev_Gas28.jpg]

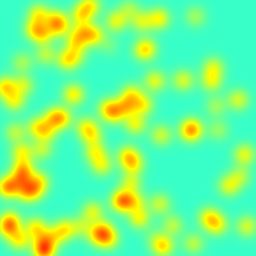

Supplement: S6 File — (ZIP) [file pone.0158590.s006.zip › anaph/movie_anaph_IrrBowSyne_BubCham_Diphth_GamThe_JetTur_UncPri_DopEff_YelFev_Gas29.jpg]

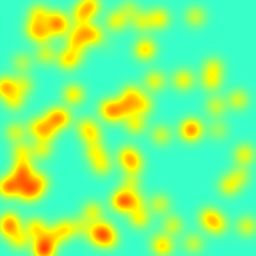

Supplement: S6 File — (ZIP) [file pone.0158590.s006.zip › anaph/movie_anaph_IrrBowSyne_BubCham_Diphth_GamThe_JetTur_UncPri_DopEff_YelFev_Gas30.jpg]

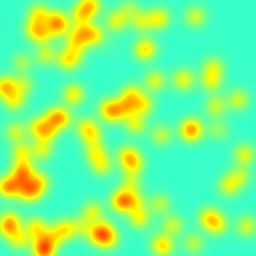

Supplement: S6 File — (ZIP) [file pone.0158590.s006.zip › anaph/movie_anaph_IrrBowSyne_BubCham_Diphth_GamThe_JetTur_UncPri_DopEff_YelFev_Gas31.jpg]

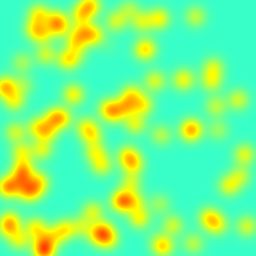

Supplement: S6 File — (ZIP) [file pone.0158590.s006.zip › anaph/movie_anaph_IrrBowSyne_BubCham_Diphth_GamThe_JetTur_UncPri_DopEff_YelFev_Gas32.jpg]

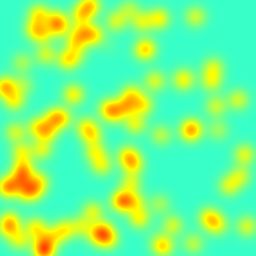

Supplement: S6 File — (ZIP) [file pone.0158590.s006.zip › anaph/movie_anaph_IrrBowSyne_BubCham_Diphth_GamThe_JetTur_UncPri_DopEff_YelFev_Gas33.jpg]

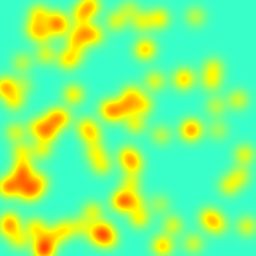

Supplement: S6 File — (ZIP) [file pone.0158590.s006.zip › anaph/movie_anaph_IrrBowSyne_BubCham_Diphth_GamThe_JetTur_UncPri_DopEff_YelFev_Gas34.jpg]

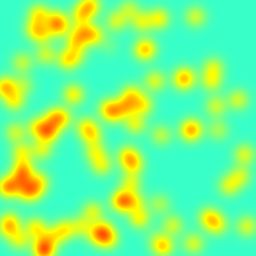

Supplement: S6 File — (ZIP) [file pone.0158590.s006.zip › anaph/movie_anaph_IrrBowSyne_BubCham_Diphth_GamThe_JetTur_UncPri_DopEff_YelFev_Gas35.jpg]

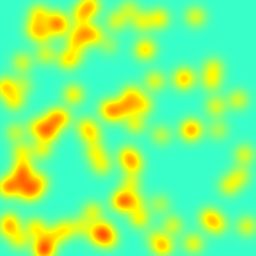

Supplement: S6 File — (ZIP) [file pone.0158590.s006.zip › anaph/movie_anaph_IrrBowSyne_BubCham_Diphth_GamThe_JetTur_UncPri_DopEff_YelFev_Gas36.jpg]

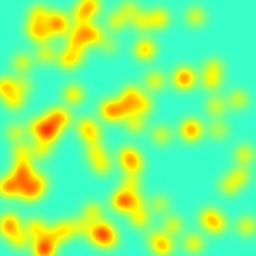

Supplement: S6 File — (ZIP) [file pone.0158590.s006.zip › anaph/movie_anaph_IrrBowSyne_BubCham_Diphth_GamThe_JetTur_UncPri_DopEff_YelFev_Gas37.jpg]

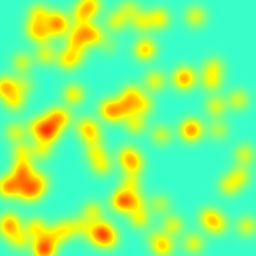

Supplement: S6 File — (ZIP) [file pone.0158590.s006.zip › anaph/movie_anaph_IrrBowSyne_BubCham_Diphth_GamThe_JetTur_UncPri_DopEff_YelFev_Gas38.jpg]

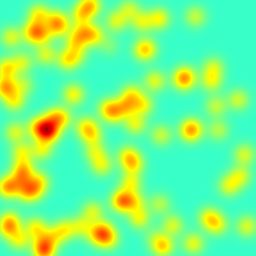

Supplement: S6 File — (ZIP) [file pone.0158590.s006.zip › anaph/movie_anaph_IrrBowSyne_BubCham_Diphth_GamThe_JetTur_UncPri_DopEff_YelFev_Gas39.jpg]

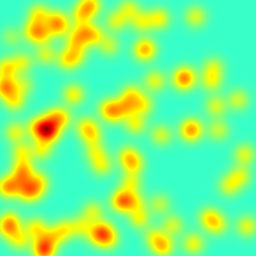

Supplement: S6 File — (ZIP) [file pone.0158590.s006.zip › anaph/movie_anaph_IrrBowSyne_BubCham_Diphth_GamThe_JetTur_UncPri_DopEff_YelFev_Gas40.jpg]

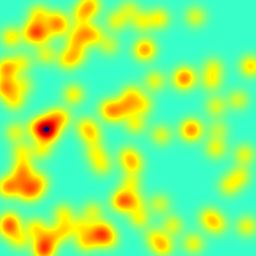

Supplement: S6 File — (ZIP) [file pone.0158590.s006.zip › anaph/movie_anaph_IrrBowSyne_BubCham_Diphth_GamThe_JetTur_UncPri_DopEff_YelFev_Gas41.jpg]

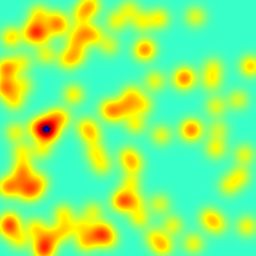

Supplement: S6 File — (ZIP) [file pone.0158590.s006.zip › anaph/movie_anaph_IrrBowSyne_BubCham_Diphth_GamThe_JetTur_UncPri_DopEff_YelFev_Gas42.jpg]

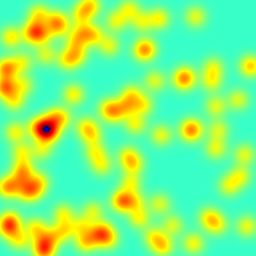

Supplement: S6 File — (ZIP) [file pone.0158590.s006.zip › anaph/movie_anaph_IrrBowSyne_BubCham_Diphth_GamThe_JetTur_UncPri_DopEff_YelFev_Gas43.jpg]

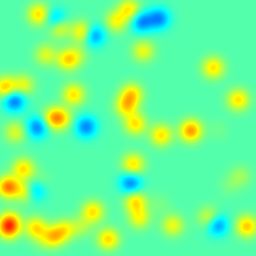

Supplement: S6 File — (ZIP) [file pone.0158590.s006.zip › anaphylaxis/movie_WoSPubmed_anaphylaxis_Diphtheria_YellowFever_InVitroFertilization_Gastritis01.jpg]

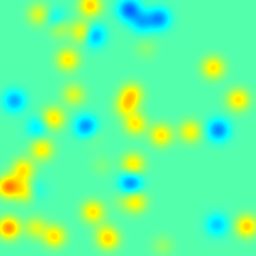

Supplement: S6 File — (ZIP) [file pone.0158590.s006.zip › anaphylaxis/movie_WoSPubmed_anaphylaxis_Diphtheria_YellowFever_InVitroFertilization_Gastritis02.jpg]

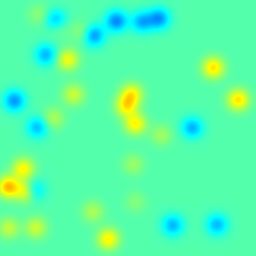

Supplement: S6 File — (ZIP) [file pone.0158590.s006.zip › anaphylaxis/movie_WoSPubmed_anaphylaxis_Diphtheria_YellowFever_InVitroFertilization_Gastritis03.jpg]

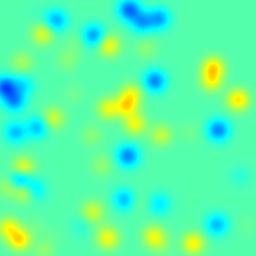

Supplement: S6 File — (ZIP) [file pone.0158590.s006.zip › anaphylaxis/movie_WoSPubmed_anaphylaxis_Diphtheria_YellowFever_InVitroFertilization_Gastritis04.jpg]

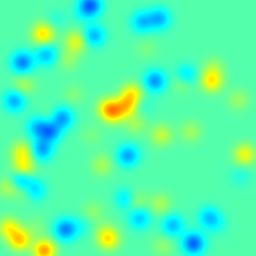

Supplement: S6 File — (ZIP) [file pone.0158590.s006.zip › anaphylaxis/movie_WoSPubmed_anaphylaxis_Diphtheria_YellowFever_InVitroFertilization_Gastritis05.jpg]

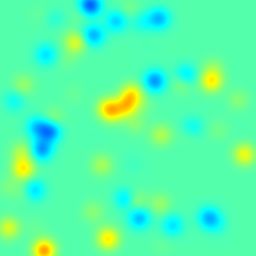

Supplement: S6 File — (ZIP) [file pone.0158590.s006.zip › anaphylaxis/movie_WoSPubmed_anaphylaxis_Diphtheria_YellowFever_InVitroFertilization_Gastritis06.jpg]

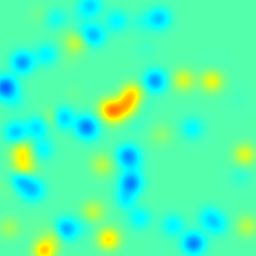

Supplement: S6 File — (ZIP) [file pone.0158590.s006.zip › anaphylaxis/movie_WoSPubmed_anaphylaxis_Diphtheria_YellowFever_InVitroFertilization_Gastritis07.jpg]

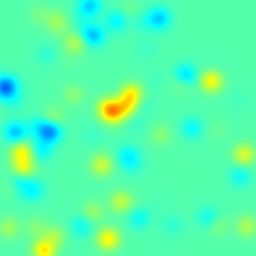

Supplement: S6 File — (ZIP) [file pone.0158590.s006.zip › anaphylaxis/movie_WoSPubmed_anaphylaxis_Diphtheria_YellowFever_InVitroFertilization_Gastritis08.jpg]

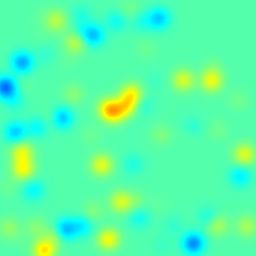

Supplement: S6 File — (ZIP) [file pone.0158590.s006.zip › anaphylaxis/movie_WoSPubmed_anaphylaxis_Diphtheria_YellowFever_InVitroFertilization_Gastritis09.jpg]

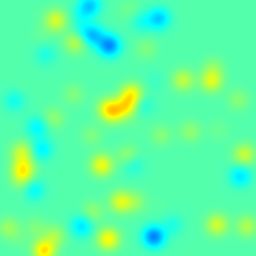

Supplement: S6 File — (ZIP) [file pone.0158590.s006.zip › anaphylaxis/movie_WoSPubmed_anaphylaxis_Diphtheria_YellowFever_InVitroFertilization_Gastritis10.jpg]

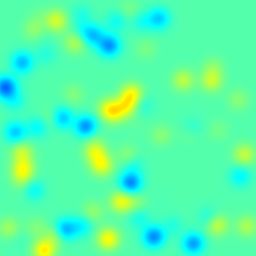

Supplement: S6 File — (ZIP) [file pone.0158590.s006.zip › anaphylaxis/movie_WoSPubmed_anaphylaxis_Diphtheria_YellowFever_InVitroFertilization_Gastritis11.jpg]

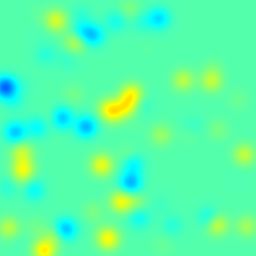

Supplement: S6 File — (ZIP) [file pone.0158590.s006.zip › anaphylaxis/movie_WoSPubmed_anaphylaxis_Diphtheria_YellowFever_InVitroFertilization_Gastritis12.jpg]

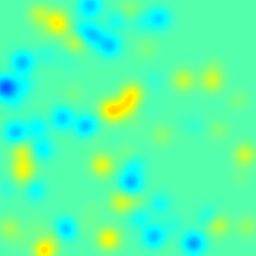

Supplement: S6 File — (ZIP) [file pone.0158590.s006.zip › anaphylaxis/movie_WoSPubmed_anaphylaxis_Diphtheria_YellowFever_InVitroFertilization_Gastritis13.jpg]

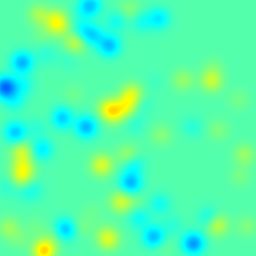

Supplement: S6 File — (ZIP) [file pone.0158590.s006.zip › anaphylaxis/movie_WoSPubmed_anaphylaxis_Diphtheria_YellowFever_InVitroFertilization_Gastritis14.jpg]

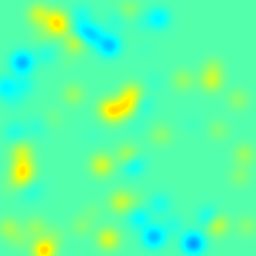

Supplement: S6 File — (ZIP) [file pone.0158590.s006.zip › anaphylaxis/movie_WoSPubmed_anaphylaxis_Diphtheria_YellowFever_InVitroFertilization_Gastritis15.jpg]

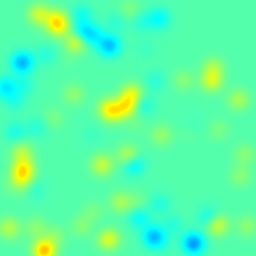

Supplement: S6 File — (ZIP) [file pone.0158590.s006.zip › anaphylaxis/movie_WoSPubmed_anaphylaxis_Diphtheria_YellowFever_InVitroFertilization_Gastritis16.jpg]

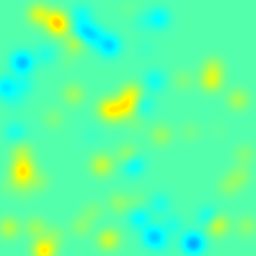

Supplement: S6 File — (ZIP) [file pone.0158590.s006.zip › anaphylaxis/movie_WoSPubmed_anaphylaxis_Diphtheria_YellowFever_InVitroFertilization_Gastritis17.jpg]

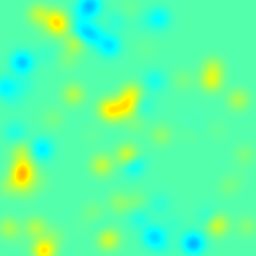

Supplement: S6 File — (ZIP) [file pone.0158590.s006.zip › anaphylaxis/movie_WoSPubmed_anaphylaxis_Diphtheria_YellowFever_InVitroFertilization_Gastritis18.jpg]

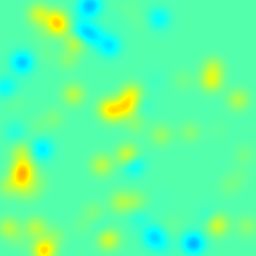

Supplement: S6 File — (ZIP) [file pone.0158590.s006.zip › anaphylaxis/movie_WoSPubmed_anaphylaxis_Diphtheria_YellowFever_InVitroFertilization_Gastritis19.jpg]

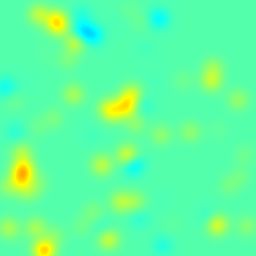

Supplement: S6 File — (ZIP) [file pone.0158590.s006.zip › anaphylaxis/movie_WoSPubmed_anaphylaxis_Diphtheria_YellowFever_InVitroFertilization_Gastritis20.jpg]

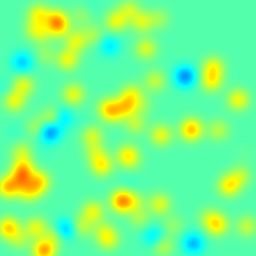

Supplement: S6 File — (ZIP) [file pone.0158590.s006.zip › anaphylaxis/movie_WoSPubmed_anaphylaxis_Diphtheria_YellowFever_InVitroFertilization_Gastritis21.jpg]

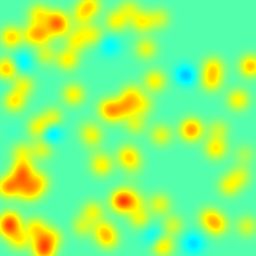

Supplement: S6 File — (ZIP) [file pone.0158590.s006.zip › anaphylaxis/movie_WoSPubmed_anaphylaxis_Diphtheria_YellowFever_InVitroFertilization_Gastritis22.jpg]

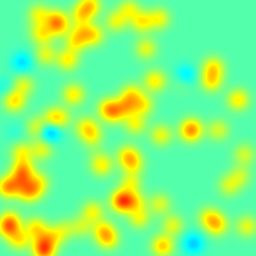

Supplement: S6 File — (ZIP) [file pone.0158590.s006.zip › anaphylaxis/movie_WoSPubmed_anaphylaxis_Diphtheria_YellowFever_InVitroFertilization_Gastritis23.jpg]

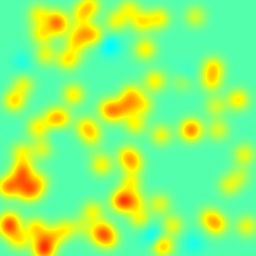

Supplement: S6 File — (ZIP) [file pone.0158590.s006.zip › anaphylaxis/movie_WoSPubmed_anaphylaxis_Diphtheria_YellowFever_InVitroFertilization_Gastritis24.jpg]

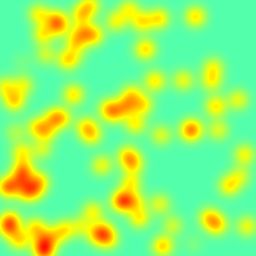

Supplement: S6 File — (ZIP) [file pone.0158590.s006.zip › anaphylaxis/movie_WoSPubmed_anaphylaxis_Diphtheria_YellowFever_InVitroFertilization_Gastritis25.jpg]

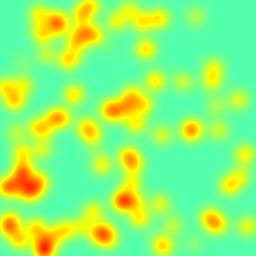

Supplement: S6 File — (ZIP) [file pone.0158590.s006.zip › anaphylaxis/movie_WoSPubmed_anaphylaxis_Diphtheria_YellowFever_InVitroFertilization_Gastritis26.jpg]

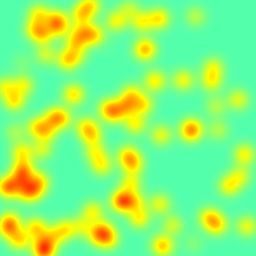

Supplement: S6 File — (ZIP) [file pone.0158590.s006.zip › anaphylaxis/movie_WoSPubmed_anaphylaxis_Diphtheria_YellowFever_InVitroFertilization_Gastritis27.jpg]

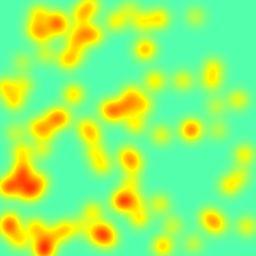

Supplement: S6 File — (ZIP) [file pone.0158590.s006.zip › anaphylaxis/movie_WoSPubmed_anaphylaxis_Diphtheria_YellowFever_InVitroFertilization_Gastritis28.jpg]

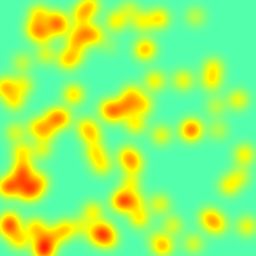

Supplement: S6 File — (ZIP) [file pone.0158590.s006.zip › anaphylaxis/movie_WoSPubmed_anaphylaxis_Diphtheria_YellowFever_InVitroFertilization_Gastritis29.jpg]

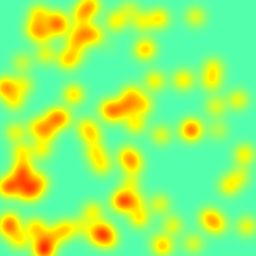

Supplement: S6 File — (ZIP) [file pone.0158590.s006.zip › anaphylaxis/movie_WoSPubmed_anaphylaxis_Diphtheria_YellowFever_InVitroFertilization_Gastritis30.jpg]

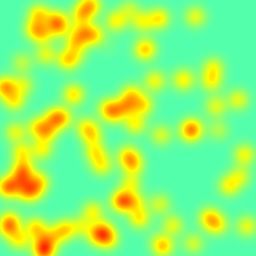

Supplement: S6 File — (ZIP) [file pone.0158590.s006.zip › anaphylaxis/movie_WoSPubmed_anaphylaxis_Diphtheria_YellowFever_InVitroFertilization_Gastritis31.jpg]

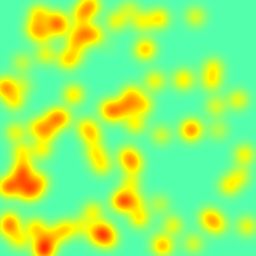

Supplement: S6 File — (ZIP) [file pone.0158590.s006.zip › anaphylaxis/movie_WoSPubmed_anaphylaxis_Diphtheria_YellowFever_InVitroFertilization_Gastritis32.jpg]

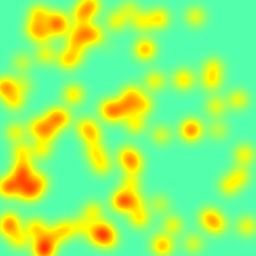

Supplement: S6 File — (ZIP) [file pone.0158590.s006.zip › anaphylaxis/movie_WoSPubmed_anaphylaxis_Diphtheria_YellowFever_InVitroFertilization_Gastritis33.jpg]

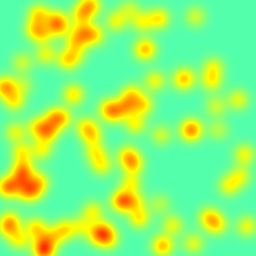

Supplement: S6 File — (ZIP) [file pone.0158590.s006.zip › anaphylaxis/movie_WoSPubmed_anaphylaxis_Diphtheria_YellowFever_InVitroFertilization_Gastritis34.jpg]

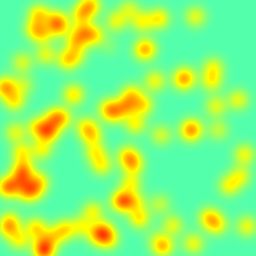

Supplement: S6 File — (ZIP) [file pone.0158590.s006.zip › anaphylaxis/movie_WoSPubmed_anaphylaxis_Diphtheria_YellowFever_InVitroFertilization_Gastritis35.jpg]

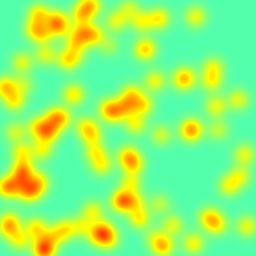

Supplement: S6 File — (ZIP) [file pone.0158590.s006.zip › anaphylaxis/movie_WoSPubmed_anaphylaxis_Diphtheria_YellowFever_InVitroFertilization_Gastritis36.jpg]

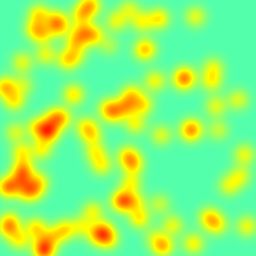

Supplement: S6 File — (ZIP) [file pone.0158590.s006.zip › anaphylaxis/movie_WoSPubmed_anaphylaxis_Diphtheria_YellowFever_InVitroFertilization_Gastritis37.jpg]

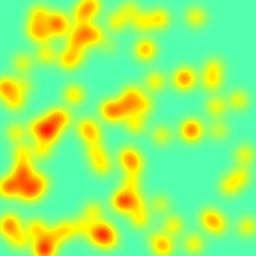

Supplement: S6 File — (ZIP) [file pone.0158590.s006.zip › anaphylaxis/movie_WoSPubmed_anaphylaxis_Diphtheria_YellowFever_InVitroFertilization_Gastritis38.jpg]

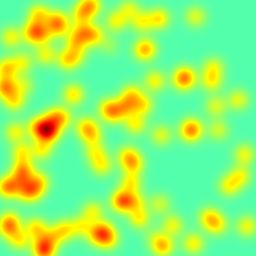

Supplement: S6 File — (ZIP) [file pone.0158590.s006.zip › anaphylaxis/movie_WoSPubmed_anaphylaxis_Diphtheria_YellowFever_InVitroFertilization_Gastritis39.jpg]

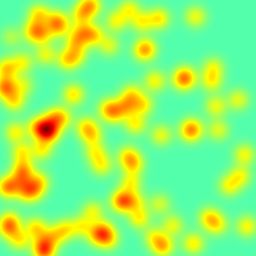

Supplement: S6 File — (ZIP) [file pone.0158590.s006.zip › anaphylaxis/movie_WoSPubmed_anaphylaxis_Diphtheria_YellowFever_InVitroFertilization_Gastritis40.jpg]

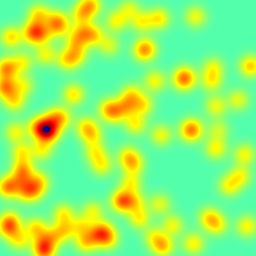

Supplement: S6 File — (ZIP) [file pone.0158590.s006.zip › anaphylaxis/movie_WoSPubmed_anaphylaxis_Diphtheria_YellowFever_InVitroFertilization_Gastritis41.jpg]

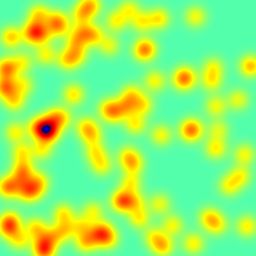

Supplement: S6 File — (ZIP) [file pone.0158590.s006.zip › anaphylaxis/movie_WoSPubmed_anaphylaxis_Diphtheria_YellowFever_InVitroFertilization_Gastritis42.jpg]

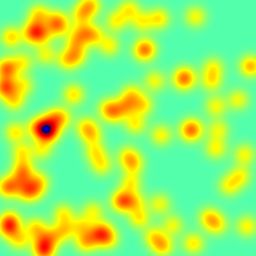

Supplement: S6 File — (ZIP) [file pone.0158590.s006.zip › anaphylaxis/movie_WoSPubmed_anaphylaxis_Diphtheria_YellowFever_InVitroFertilization_Gastritis43.jpg]

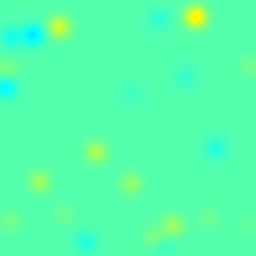

Supplement: S6 File — (ZIP) [file pone.0158590.s006.zip › anaphylaxis_/movie_WoSPubmed_anaphylaxis_Diphtheria_YellowFever_InVitroFertilization_Gastritis100.jpg]

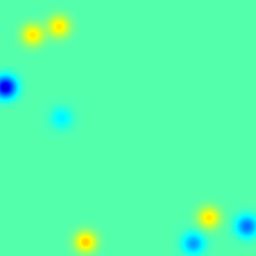

Supplement: S6 File — (ZIP) [file pone.0158590.s006.zip › anaphylaxis_/movie_WoSPubmed_anaphylaxis_Diphtheria_YellowFever_InVitroFertilization_Gastritis101.jpg]

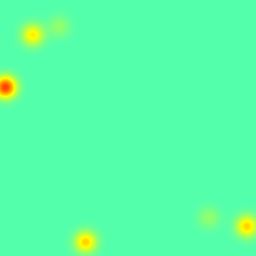

Supplement: S6 File — (ZIP) [file pone.0158590.s006.zip › anaphylaxis_/movie_WoSPubmed_anaphylaxis_Diphtheria_YellowFever_InVitroFertilization_Gastritis102.jpg]

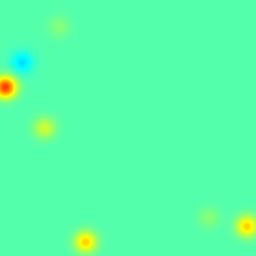

Supplement: S6 File — (ZIP) [file pone.0158590.s006.zip › anaphylaxis_/movie_WoSPubmed_anaphylaxis_Diphtheria_YellowFever_InVitroFertilization_Gastritis103.jpg]

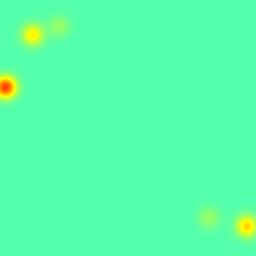

Supplement: S6 File — (ZIP) [file pone.0158590.s006.zip › anaphylaxis_/movie_WoSPubmed_anaphylaxis_Diphtheria_YellowFever_InVitroFertilization_Gastritis104.jpg]

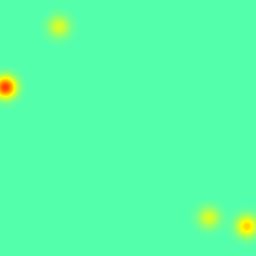

Supplement: S6 File — (ZIP) [file pone.0158590.s006.zip › anaphylaxis_/movie_WoSPubmed_anaphylaxis_Diphtheria_YellowFever_InVitroFertilization_Gastritis105.jpg]

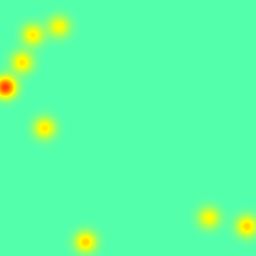

Supplement: S6 File — (ZIP) [file pone.0158590.s006.zip › anaphylaxis_/movie_WoSPubmed_anaphylaxis_Diphtheria_YellowFever_InVitroFertilization_Gastritis106.jpg]

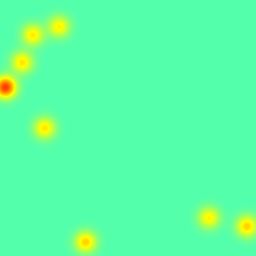

Supplement: S6 File — (ZIP) [file pone.0158590.s006.zip › anaphylaxis_/movie_WoSPubmed_anaphylaxis_Diphtheria_YellowFever_InVitroFertilization_Gastritis107.jpg]

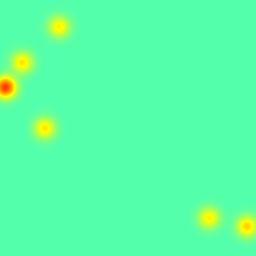

Supplement: S6 File — (ZIP) [file pone.0158590.s006.zip › anaphylaxis_/movie_WoSPubmed_anaphylaxis_Diphtheria_YellowFever_InVitroFertilization_Gastritis108.jpg]

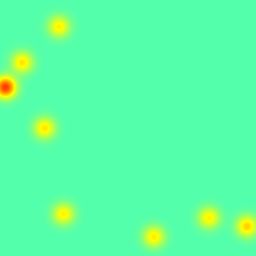

Supplement: S6 File — (ZIP) [file pone.0158590.s006.zip › anaphylaxis_/movie_WoSPubmed_anaphylaxis_Diphtheria_YellowFever_InVitroFertilization_Gastritis109.jpg]

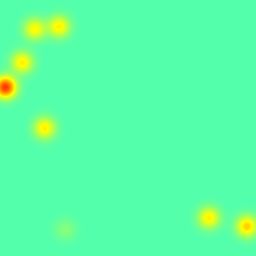

Supplement: S6 File — (ZIP) [file pone.0158590.s006.zip › anaphylaxis_/movie_WoSPubmed_anaphylaxis_Diphtheria_YellowFever_InVitroFertilization_Gastritis110.jpg]

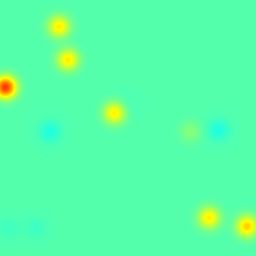

Supplement: S6 File — (ZIP) [file pone.0158590.s006.zip › anaphylaxis_/movie_WoSPubmed_anaphylaxis_Diphtheria_YellowFever_InVitroFertilization_Gastritis111.jpg]

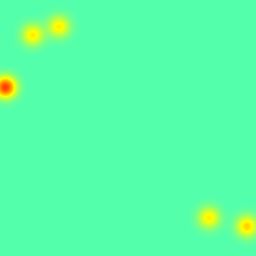

Supplement: S6 File — (ZIP) [file pone.0158590.s006.zip › anaphylaxis_/movie_WoSPubmed_anaphylaxis_Diphtheria_YellowFever_InVitroFertilization_Gastritis112.jpg]

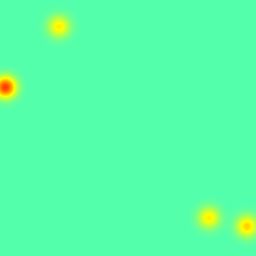

Supplement: S6 File — (ZIP) [file pone.0158590.s006.zip › anaphylaxis_/movie_WoSPubmed_anaphylaxis_Diphtheria_YellowFever_InVitroFertilization_Gastritis113.jpg]
