# Supplementary figures and images for: Invisible Brain: Knowledge in Research Works and Neuron Activity (part 4 of 6)
Source: PLoS One. 2016 Jul 20;11(7):e0158590. doi: 10.1371/journal.pone.0158590 (PMC4954711; doi:10.1371/journal.pone.0158590)

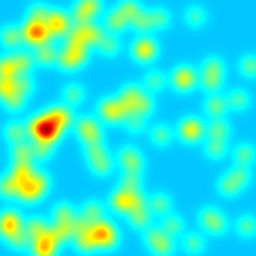

Supplement: S7 File — (ZIP) [file pone.0158590.s007.zip › iNet1_Size100_CC01inh/movie_iNet1_Size100_CC01inh_anaphylaxis_765_top100838.jpg]

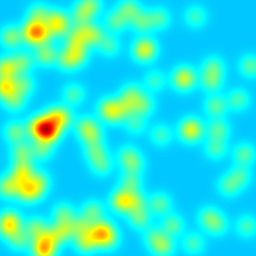

Supplement: S7 File — (ZIP) [file pone.0158590.s007.zip › iNet1_Size100_CC01inh/movie_iNet1_Size100_CC01inh_anaphylaxis_765_top100839.jpg]

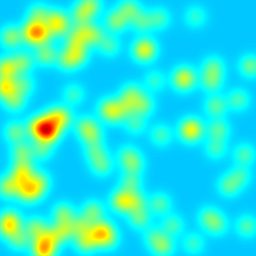

Supplement: S7 File — (ZIP) [file pone.0158590.s007.zip › iNet1_Size100_CC01inh/movie_iNet1_Size100_CC01inh_anaphylaxis_765_top100840.jpg]

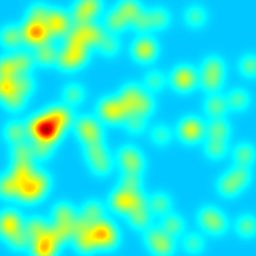

Supplement: S7 File — (ZIP) [file pone.0158590.s007.zip › iNet1_Size100_CC01inh/movie_iNet1_Size100_CC01inh_anaphylaxis_765_top100841.jpg]

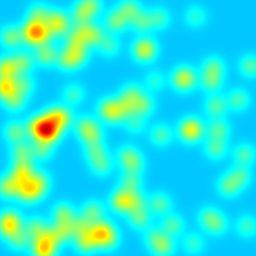

Supplement: S7 File — (ZIP) [file pone.0158590.s007.zip › iNet1_Size100_CC01inh/movie_iNet1_Size100_CC01inh_anaphylaxis_765_top100842.jpg]

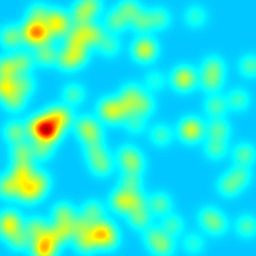

Supplement: S7 File — (ZIP) [file pone.0158590.s007.zip › iNet1_Size100_CC01inh/movie_iNet1_Size100_CC01inh_anaphylaxis_765_top100843.jpg]

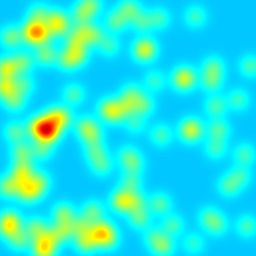

Supplement: S7 File — (ZIP) [file pone.0158590.s007.zip › iNet1_Size100_CC01inh/movie_iNet1_Size100_CC01inh_anaphylaxis_765_top100844.jpg]

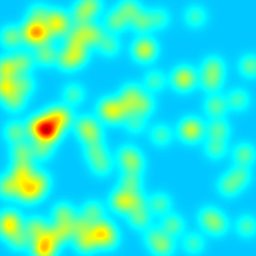

Supplement: S7 File — (ZIP) [file pone.0158590.s007.zip › iNet1_Size100_CC01inh/movie_iNet1_Size100_CC01inh_anaphylaxis_765_top100845.jpg]

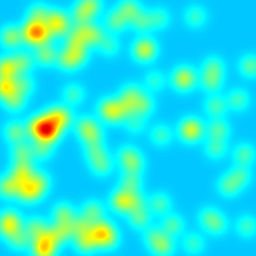

Supplement: S7 File — (ZIP) [file pone.0158590.s007.zip › iNet1_Size100_CC01inh/movie_iNet1_Size100_CC01inh_anaphylaxis_765_top100846.jpg]

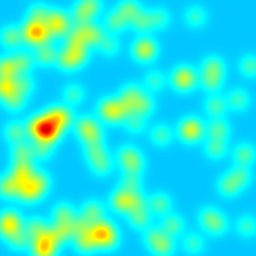

Supplement: S7 File — (ZIP) [file pone.0158590.s007.zip › iNet1_Size100_CC01inh/movie_iNet1_Size100_CC01inh_anaphylaxis_765_top100847.jpg]

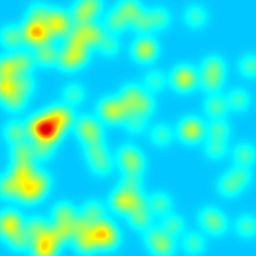

Supplement: S7 File — (ZIP) [file pone.0158590.s007.zip › iNet1_Size100_CC01inh/movie_iNet1_Size100_CC01inh_anaphylaxis_765_top100848.jpg]

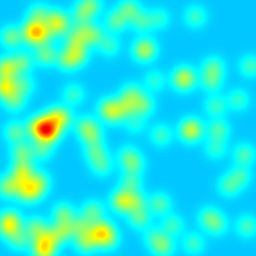

Supplement: S7 File — (ZIP) [file pone.0158590.s007.zip › iNet1_Size100_CC01inh/movie_iNet1_Size100_CC01inh_anaphylaxis_765_top100849.jpg]

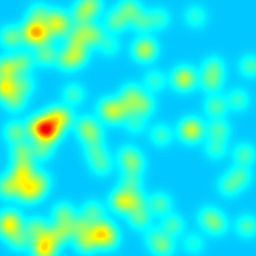

Supplement: S7 File — (ZIP) [file pone.0158590.s007.zip › iNet1_Size100_CC01inh/movie_iNet1_Size100_CC01inh_anaphylaxis_765_top100850.jpg]

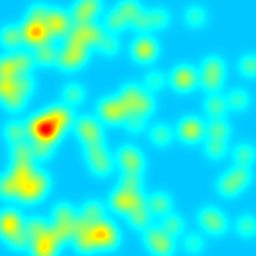

Supplement: S7 File — (ZIP) [file pone.0158590.s007.zip › iNet1_Size100_CC01inh/movie_iNet1_Size100_CC01inh_anaphylaxis_765_top100851.jpg]

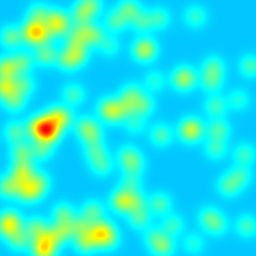

Supplement: S7 File — (ZIP) [file pone.0158590.s007.zip › iNet1_Size100_CC01inh/movie_iNet1_Size100_CC01inh_anaphylaxis_765_top100852.jpg]

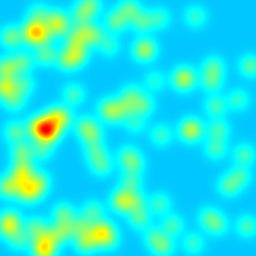

Supplement: S7 File — (ZIP) [file pone.0158590.s007.zip › iNet1_Size100_CC01inh/movie_iNet1_Size100_CC01inh_anaphylaxis_765_top100853.jpg]

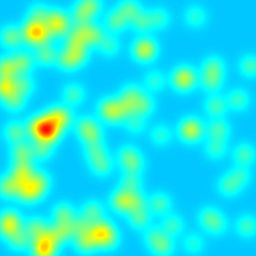

Supplement: S7 File — (ZIP) [file pone.0158590.s007.zip › iNet1_Size100_CC01inh/movie_iNet1_Size100_CC01inh_anaphylaxis_765_top100854.jpg]

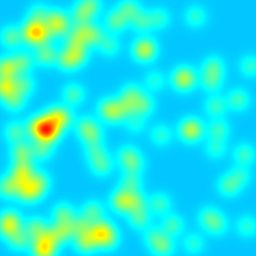

Supplement: S7 File — (ZIP) [file pone.0158590.s007.zip › iNet1_Size100_CC01inh/movie_iNet1_Size100_CC01inh_anaphylaxis_765_top100855.jpg]

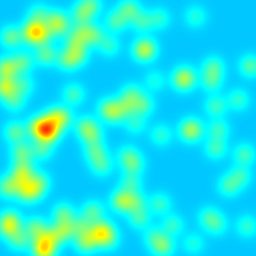

Supplement: S7 File — (ZIP) [file pone.0158590.s007.zip › iNet1_Size100_CC01inh/movie_iNet1_Size100_CC01inh_anaphylaxis_765_top100856.jpg]

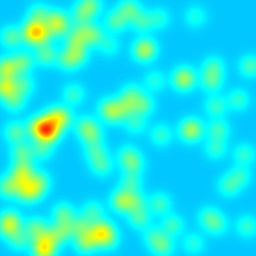

Supplement: S7 File — (ZIP) [file pone.0158590.s007.zip › iNet1_Size100_CC01inh/movie_iNet1_Size100_CC01inh_anaphylaxis_765_top100857.jpg]

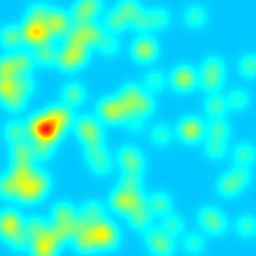

Supplement: S7 File — (ZIP) [file pone.0158590.s007.zip › iNet1_Size100_CC01inh/movie_iNet1_Size100_CC01inh_anaphylaxis_765_top100858.jpg]

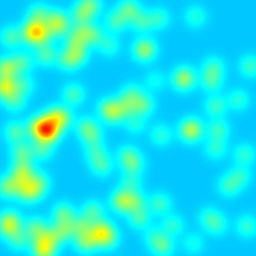

Supplement: S7 File — (ZIP) [file pone.0158590.s007.zip › iNet1_Size100_CC01inh/movie_iNet1_Size100_CC01inh_anaphylaxis_765_top100859.jpg]

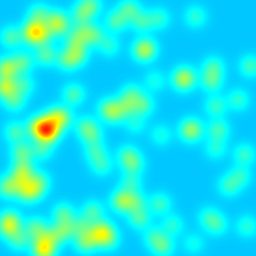

Supplement: S7 File — (ZIP) [file pone.0158590.s007.zip › iNet1_Size100_CC01inh/movie_iNet1_Size100_CC01inh_anaphylaxis_765_top100860.jpg]

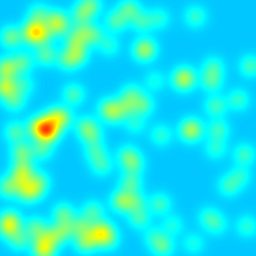

Supplement: S7 File — (ZIP) [file pone.0158590.s007.zip › iNet1_Size100_CC01inh/movie_iNet1_Size100_CC01inh_anaphylaxis_765_top100861.jpg]

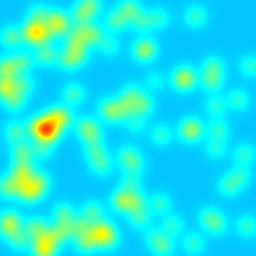

Supplement: S7 File — (ZIP) [file pone.0158590.s007.zip › iNet1_Size100_CC01inh/movie_iNet1_Size100_CC01inh_anaphylaxis_765_top100862.jpg]

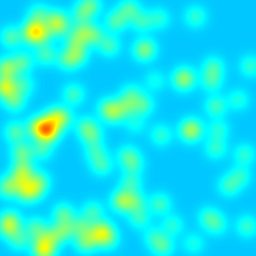

Supplement: S7 File — (ZIP) [file pone.0158590.s007.zip › iNet1_Size100_CC01inh/movie_iNet1_Size100_CC01inh_anaphylaxis_765_top100863.jpg]

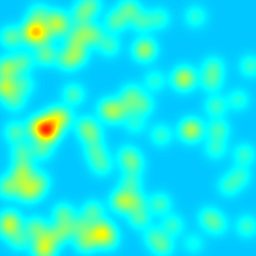

Supplement: S7 File — (ZIP) [file pone.0158590.s007.zip › iNet1_Size100_CC01inh/movie_iNet1_Size100_CC01inh_anaphylaxis_765_top100864.jpg]

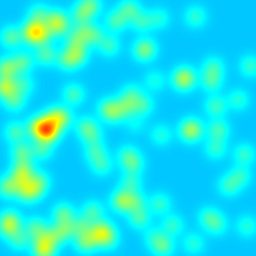

Supplement: S7 File — (ZIP) [file pone.0158590.s007.zip › iNet1_Size100_CC01inh/movie_iNet1_Size100_CC01inh_anaphylaxis_765_top100865.jpg]

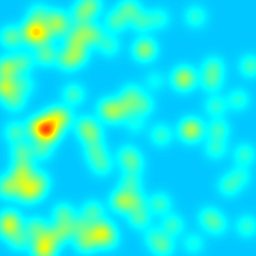

Supplement: S7 File — (ZIP) [file pone.0158590.s007.zip › iNet1_Size100_CC01inh/movie_iNet1_Size100_CC01inh_anaphylaxis_765_top100866.jpg]

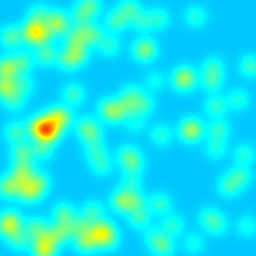

Supplement: S7 File — (ZIP) [file pone.0158590.s007.zip › iNet1_Size100_CC01inh/movie_iNet1_Size100_CC01inh_anaphylaxis_765_top100867.jpg]

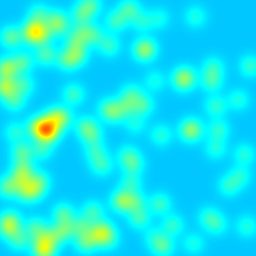

Supplement: S7 File — (ZIP) [file pone.0158590.s007.zip › iNet1_Size100_CC01inh/movie_iNet1_Size100_CC01inh_anaphylaxis_765_top100868.jpg]

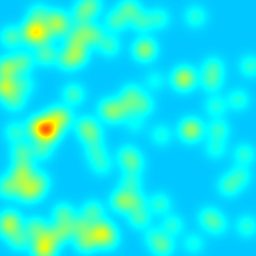

Supplement: S7 File — (ZIP) [file pone.0158590.s007.zip › iNet1_Size100_CC01inh/movie_iNet1_Size100_CC01inh_anaphylaxis_765_top100869.jpg]

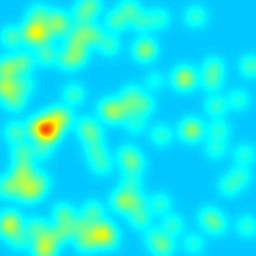

Supplement: S7 File — (ZIP) [file pone.0158590.s007.zip › iNet1_Size100_CC01inh/movie_iNet1_Size100_CC01inh_anaphylaxis_765_top100870.jpg]

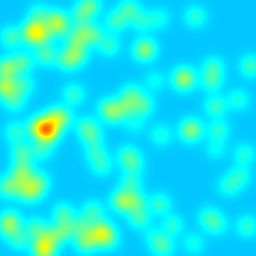

Supplement: S7 File — (ZIP) [file pone.0158590.s007.zip › iNet1_Size100_CC01inh/movie_iNet1_Size100_CC01inh_anaphylaxis_765_top100871.jpg]

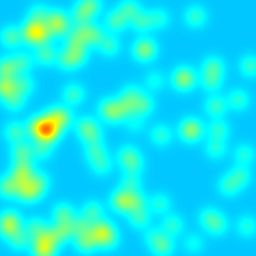

Supplement: S7 File — (ZIP) [file pone.0158590.s007.zip › iNet1_Size100_CC01inh/movie_iNet1_Size100_CC01inh_anaphylaxis_765_top100872.jpg]

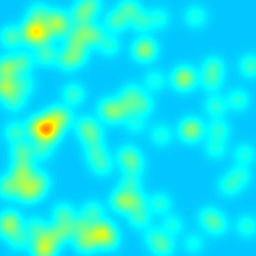

Supplement: S7 File — (ZIP) [file pone.0158590.s007.zip › iNet1_Size100_CC01inh/movie_iNet1_Size100_CC01inh_anaphylaxis_765_top100873.jpg]

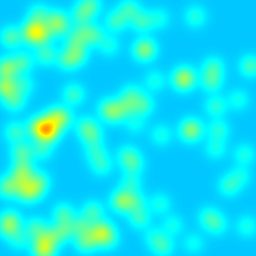

Supplement: S7 File — (ZIP) [file pone.0158590.s007.zip › iNet1_Size100_CC01inh/movie_iNet1_Size100_CC01inh_anaphylaxis_765_top100874.jpg]

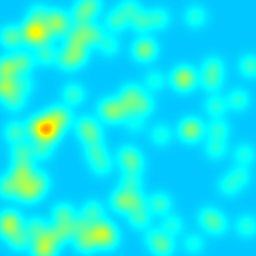

Supplement: S7 File — (ZIP) [file pone.0158590.s007.zip › iNet1_Size100_CC01inh/movie_iNet1_Size100_CC01inh_anaphylaxis_765_top100875.jpg]

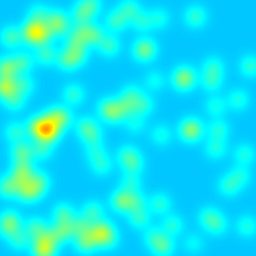

Supplement: S7 File — (ZIP) [file pone.0158590.s007.zip › iNet1_Size100_CC01inh/movie_iNet1_Size100_CC01inh_anaphylaxis_765_top100876.jpg]

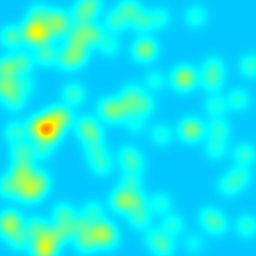

Supplement: S7 File — (ZIP) [file pone.0158590.s007.zip › iNet1_Size100_CC01inh/movie_iNet1_Size100_CC01inh_anaphylaxis_765_top100877.jpg]

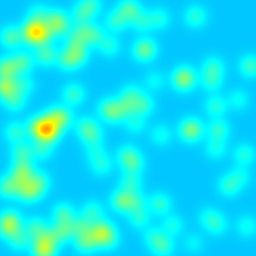

Supplement: S7 File — (ZIP) [file pone.0158590.s007.zip › iNet1_Size100_CC01inh/movie_iNet1_Size100_CC01inh_anaphylaxis_765_top100878.jpg]

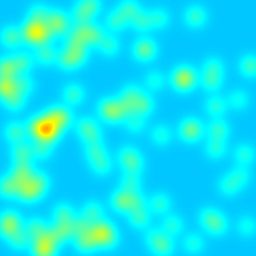

Supplement: S7 File — (ZIP) [file pone.0158590.s007.zip › iNet1_Size100_CC01inh/movie_iNet1_Size100_CC01inh_anaphylaxis_765_top100879.jpg]

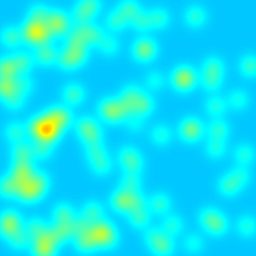

Supplement: S7 File — (ZIP) [file pone.0158590.s007.zip › iNet1_Size100_CC01inh/movie_iNet1_Size100_CC01inh_anaphylaxis_765_top100880.jpg]

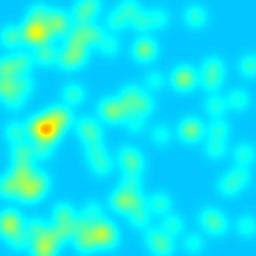

Supplement: S7 File — (ZIP) [file pone.0158590.s007.zip › iNet1_Size100_CC01inh/movie_iNet1_Size100_CC01inh_anaphylaxis_765_top100881.jpg]

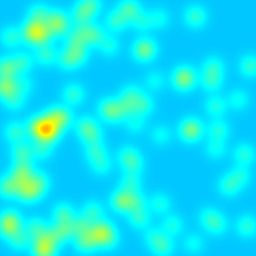

Supplement: S7 File — (ZIP) [file pone.0158590.s007.zip › iNet1_Size100_CC01inh/movie_iNet1_Size100_CC01inh_anaphylaxis_765_top100882.jpg]

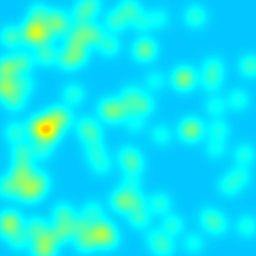

Supplement: S7 File — (ZIP) [file pone.0158590.s007.zip › iNet1_Size100_CC01inh/movie_iNet1_Size100_CC01inh_anaphylaxis_765_top100883.jpg]

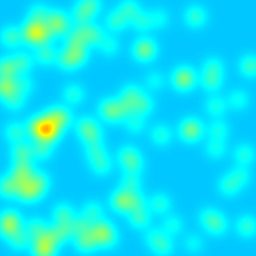

Supplement: S7 File — (ZIP) [file pone.0158590.s007.zip › iNet1_Size100_CC01inh/movie_iNet1_Size100_CC01inh_anaphylaxis_765_top100884.jpg]

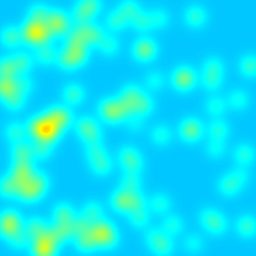

Supplement: S7 File — (ZIP) [file pone.0158590.s007.zip › iNet1_Size100_CC01inh/movie_iNet1_Size100_CC01inh_anaphylaxis_765_top100885.jpg]

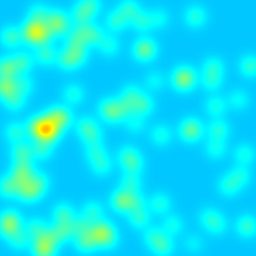

Supplement: S7 File — (ZIP) [file pone.0158590.s007.zip › iNet1_Size100_CC01inh/movie_iNet1_Size100_CC01inh_anaphylaxis_765_top100886.jpg]

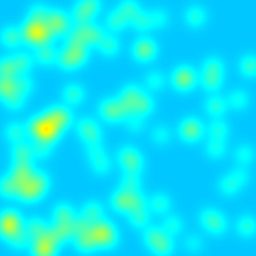

Supplement: S7 File — (ZIP) [file pone.0158590.s007.zip › iNet1_Size100_CC01inh/movie_iNet1_Size100_CC01inh_anaphylaxis_765_top100887.jpg]

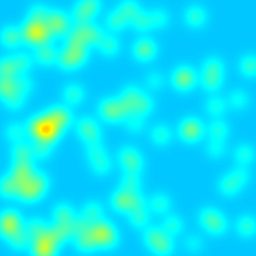

Supplement: S7 File — (ZIP) [file pone.0158590.s007.zip › iNet1_Size100_CC01inh/movie_iNet1_Size100_CC01inh_anaphylaxis_765_top100888.jpg]

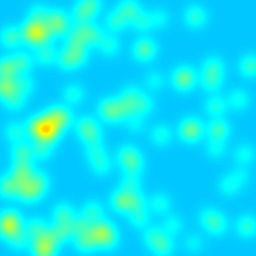

Supplement: S7 File — (ZIP) [file pone.0158590.s007.zip › iNet1_Size100_CC01inh/movie_iNet1_Size100_CC01inh_anaphylaxis_765_top100889.jpg]

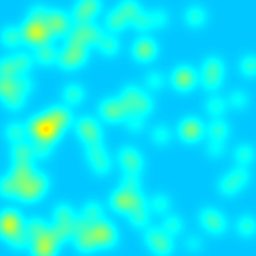

Supplement: S7 File — (ZIP) [file pone.0158590.s007.zip › iNet1_Size100_CC01inh/movie_iNet1_Size100_CC01inh_anaphylaxis_765_top100890.jpg]

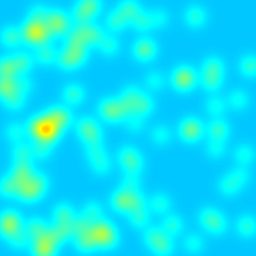

Supplement: S7 File — (ZIP) [file pone.0158590.s007.zip › iNet1_Size100_CC01inh/movie_iNet1_Size100_CC01inh_anaphylaxis_765_top100891.jpg]

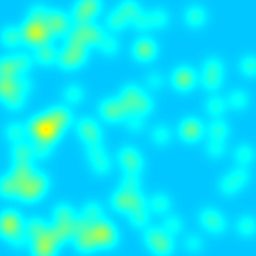

Supplement: S7 File — (ZIP) [file pone.0158590.s007.zip › iNet1_Size100_CC01inh/movie_iNet1_Size100_CC01inh_anaphylaxis_765_top100892.jpg]

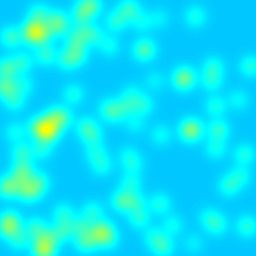

Supplement: S7 File — (ZIP) [file pone.0158590.s007.zip › iNet1_Size100_CC01inh/movie_iNet1_Size100_CC01inh_anaphylaxis_765_top100893.jpg]

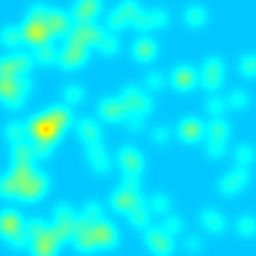

Supplement: S7 File — (ZIP) [file pone.0158590.s007.zip › iNet1_Size100_CC01inh/movie_iNet1_Size100_CC01inh_anaphylaxis_765_top100894.jpg]

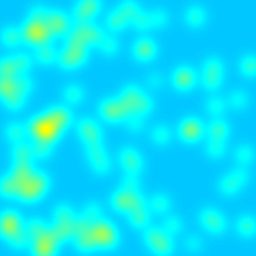

Supplement: S7 File — (ZIP) [file pone.0158590.s007.zip › iNet1_Size100_CC01inh/movie_iNet1_Size100_CC01inh_anaphylaxis_765_top100895.jpg]

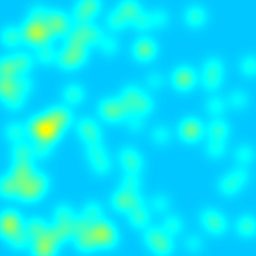

Supplement: S7 File — (ZIP) [file pone.0158590.s007.zip › iNet1_Size100_CC01inh/movie_iNet1_Size100_CC01inh_anaphylaxis_765_top100896.jpg]

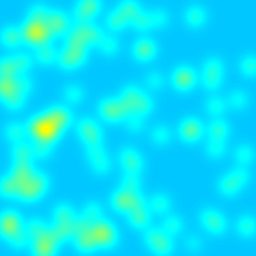

Supplement: S7 File — (ZIP) [file pone.0158590.s007.zip › iNet1_Size100_CC01inh/movie_iNet1_Size100_CC01inh_anaphylaxis_765_top100897.jpg]

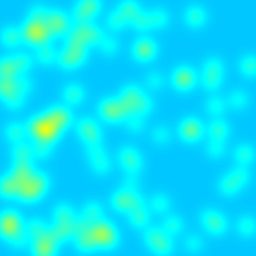

Supplement: S7 File — (ZIP) [file pone.0158590.s007.zip › iNet1_Size100_CC01inh/movie_iNet1_Size100_CC01inh_anaphylaxis_765_top100898.jpg]

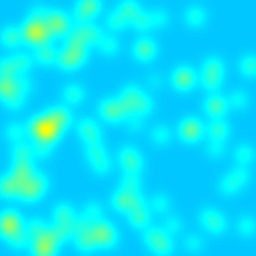

Supplement: S7 File — (ZIP) [file pone.0158590.s007.zip › iNet1_Size100_CC01inh/movie_iNet1_Size100_CC01inh_anaphylaxis_765_top100899.jpg]

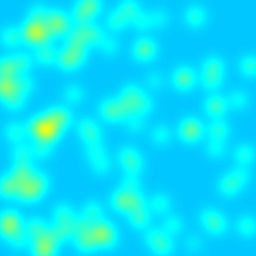

Supplement: S7 File — (ZIP) [file pone.0158590.s007.zip › iNet1_Size100_CC01inh/movie_iNet1_Size100_CC01inh_anaphylaxis_765_top100900.jpg]

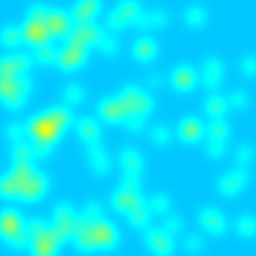

Supplement: S7 File — (ZIP) [file pone.0158590.s007.zip › iNet1_Size100_CC01inh/movie_iNet1_Size100_CC01inh_anaphylaxis_765_top100901.jpg]

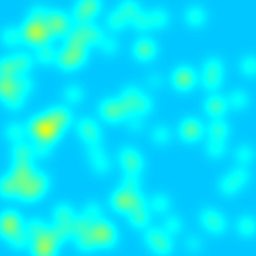

Supplement: S7 File — (ZIP) [file pone.0158590.s007.zip › iNet1_Size100_CC01inh/movie_iNet1_Size100_CC01inh_anaphylaxis_765_top100902.jpg]

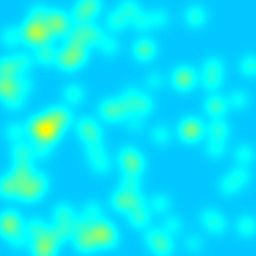

Supplement: S7 File — (ZIP) [file pone.0158590.s007.zip › iNet1_Size100_CC01inh/movie_iNet1_Size100_CC01inh_anaphylaxis_765_top100903.jpg]

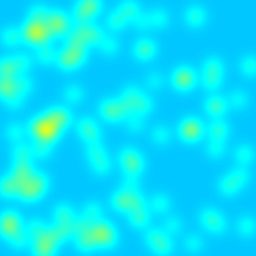

Supplement: S7 File — (ZIP) [file pone.0158590.s007.zip › iNet1_Size100_CC01inh/movie_iNet1_Size100_CC01inh_anaphylaxis_765_top100904.jpg]

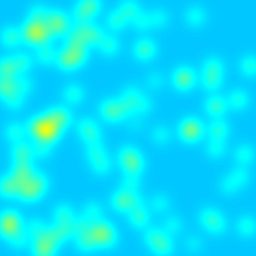

Supplement: S7 File — (ZIP) [file pone.0158590.s007.zip › iNet1_Size100_CC01inh/movie_iNet1_Size100_CC01inh_anaphylaxis_765_top100905.jpg]

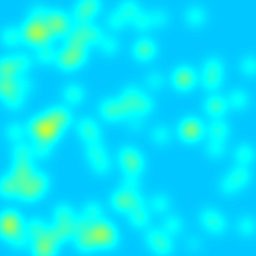

Supplement: S7 File — (ZIP) [file pone.0158590.s007.zip › iNet1_Size100_CC01inh/movie_iNet1_Size100_CC01inh_anaphylaxis_765_top100906.jpg]

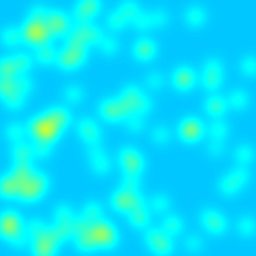

Supplement: S7 File — (ZIP) [file pone.0158590.s007.zip › iNet1_Size100_CC01inh/movie_iNet1_Size100_CC01inh_anaphylaxis_765_top100907.jpg]

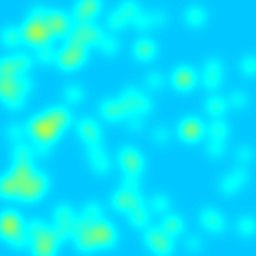

Supplement: S7 File — (ZIP) [file pone.0158590.s007.zip › iNet1_Size100_CC01inh/movie_iNet1_Size100_CC01inh_anaphylaxis_765_top100908.jpg]

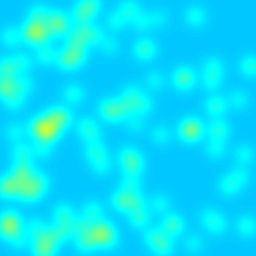

Supplement: S7 File — (ZIP) [file pone.0158590.s007.zip › iNet1_Size100_CC01inh/movie_iNet1_Size100_CC01inh_anaphylaxis_765_top100909.jpg]

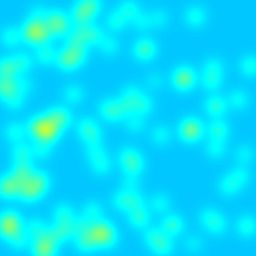

Supplement: S7 File — (ZIP) [file pone.0158590.s007.zip › iNet1_Size100_CC01inh/movie_iNet1_Size100_CC01inh_anaphylaxis_765_top100910.jpg]

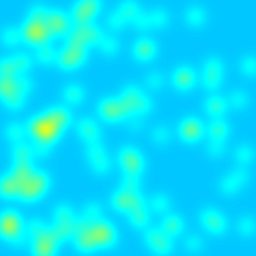

Supplement: S7 File — (ZIP) [file pone.0158590.s007.zip › iNet1_Size100_CC01inh/movie_iNet1_Size100_CC01inh_anaphylaxis_765_top100911.jpg]

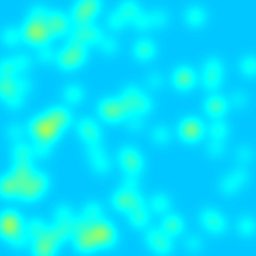

Supplement: S7 File — (ZIP) [file pone.0158590.s007.zip › iNet1_Size100_CC01inh/movie_iNet1_Size100_CC01inh_anaphylaxis_765_top100912.jpg]

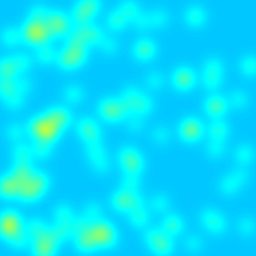

Supplement: S7 File — (ZIP) [file pone.0158590.s007.zip › iNet1_Size100_CC01inh/movie_iNet1_Size100_CC01inh_anaphylaxis_765_top100913.jpg]

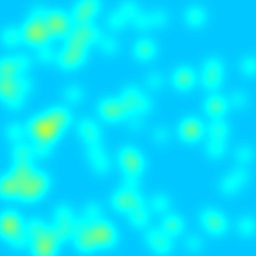

Supplement: S7 File — (ZIP) [file pone.0158590.s007.zip › iNet1_Size100_CC01inh/movie_iNet1_Size100_CC01inh_anaphylaxis_765_top100914.jpg]

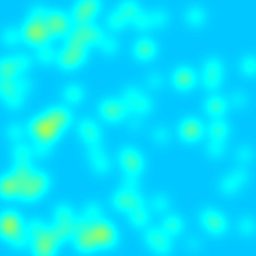

Supplement: S7 File — (ZIP) [file pone.0158590.s007.zip › iNet1_Size100_CC01inh/movie_iNet1_Size100_CC01inh_anaphylaxis_765_top100915.jpg]

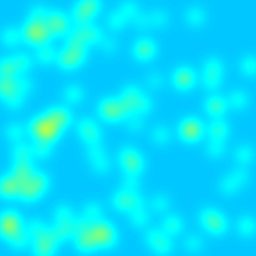

Supplement: S7 File — (ZIP) [file pone.0158590.s007.zip › iNet1_Size100_CC01inh/movie_iNet1_Size100_CC01inh_anaphylaxis_765_top100916.jpg]

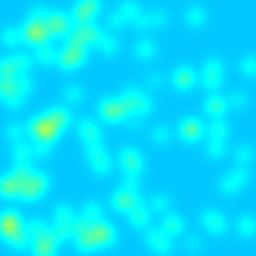

Supplement: S7 File — (ZIP) [file pone.0158590.s007.zip › iNet1_Size100_CC01inh/movie_iNet1_Size100_CC01inh_anaphylaxis_765_top100917.jpg]

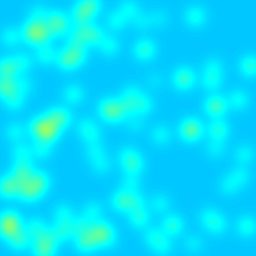

Supplement: S7 File — (ZIP) [file pone.0158590.s007.zip › iNet1_Size100_CC01inh/movie_iNet1_Size100_CC01inh_anaphylaxis_765_top100918.jpg]

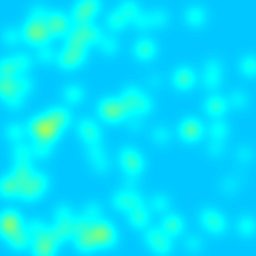

Supplement: S7 File — (ZIP) [file pone.0158590.s007.zip › iNet1_Size100_CC01inh/movie_iNet1_Size100_CC01inh_anaphylaxis_765_top100919.jpg]

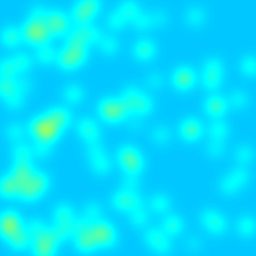

Supplement: S7 File — (ZIP) [file pone.0158590.s007.zip › iNet1_Size100_CC01inh/movie_iNet1_Size100_CC01inh_anaphylaxis_765_top100920.jpg]

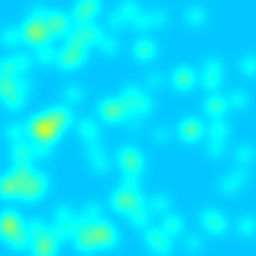

Supplement: S7 File — (ZIP) [file pone.0158590.s007.zip › iNet1_Size100_CC01inh/movie_iNet1_Size100_CC01inh_anaphylaxis_765_top100921.jpg]

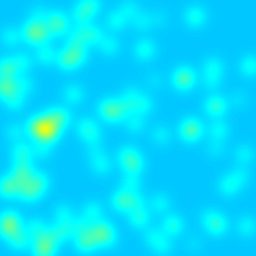

Supplement: S7 File — (ZIP) [file pone.0158590.s007.zip › iNet1_Size100_CC01inh/movie_iNet1_Size100_CC01inh_anaphylaxis_765_top100922.jpg]

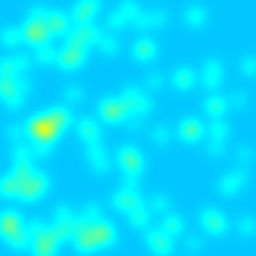

Supplement: S7 File — (ZIP) [file pone.0158590.s007.zip › iNet1_Size100_CC01inh/movie_iNet1_Size100_CC01inh_anaphylaxis_765_top100923.jpg]

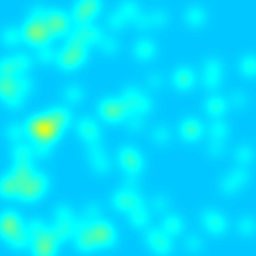

Supplement: S7 File — (ZIP) [file pone.0158590.s007.zip › iNet1_Size100_CC01inh/movie_iNet1_Size100_CC01inh_anaphylaxis_765_top100924.jpg]

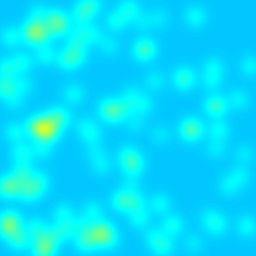

Supplement: S7 File — (ZIP) [file pone.0158590.s007.zip › iNet1_Size100_CC01inh/movie_iNet1_Size100_CC01inh_anaphylaxis_765_top100925.jpg]

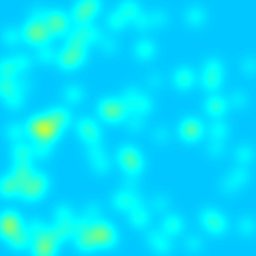

Supplement: S7 File — (ZIP) [file pone.0158590.s007.zip › iNet1_Size100_CC01inh/movie_iNet1_Size100_CC01inh_anaphylaxis_765_top100926.jpg]

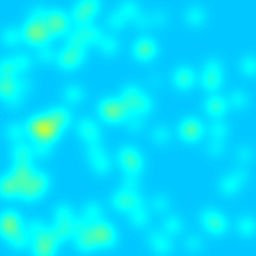

Supplement: S7 File — (ZIP) [file pone.0158590.s007.zip › iNet1_Size100_CC01inh/movie_iNet1_Size100_CC01inh_anaphylaxis_765_top100927.jpg]

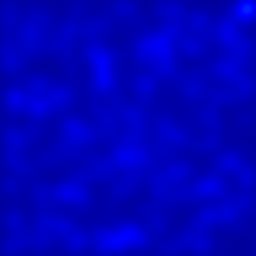

Supplement: S8 File — (ZIP) [file pone.0158590.s008.zip › movie_normal-2-original/movie_normal-2-original1.jpg]

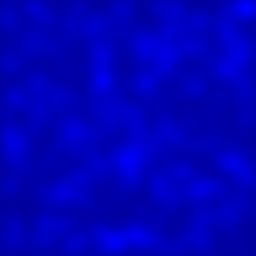

Supplement: S8 File — (ZIP) [file pone.0158590.s008.zip › movie_normal-2-original/movie_normal-2-original10.jpg]

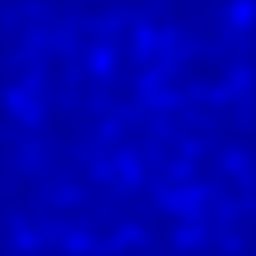

Supplement: S8 File — (ZIP) [file pone.0158590.s008.zip › movie_normal-2-original/movie_normal-2-original100.jpg]

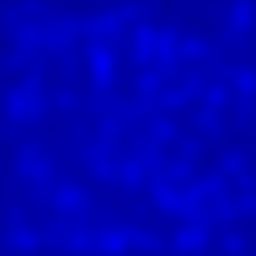

Supplement: S8 File — (ZIP) [file pone.0158590.s008.zip › movie_normal-2-original/movie_normal-2-original101.jpg]

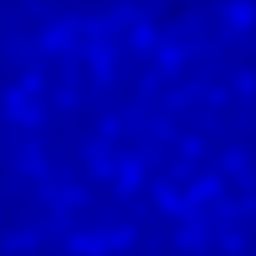

Supplement: S8 File — (ZIP) [file pone.0158590.s008.zip › movie_normal-2-original/movie_normal-2-original102.jpg]

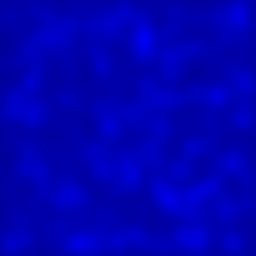

Supplement: S8 File — (ZIP) [file pone.0158590.s008.zip › movie_normal-2-original/movie_normal-2-original103.jpg]

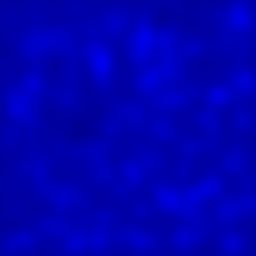

Supplement: S8 File — (ZIP) [file pone.0158590.s008.zip › movie_normal-2-original/movie_normal-2-original104.jpg]

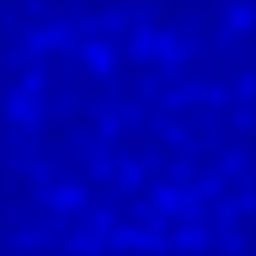

Supplement: S8 File — (ZIP) [file pone.0158590.s008.zip › movie_normal-2-original/movie_normal-2-original105.jpg]

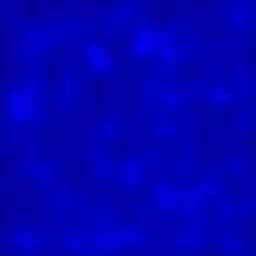

Supplement: S8 File — (ZIP) [file pone.0158590.s008.zip › movie_normal-2-original/movie_normal-2-original106.jpg]

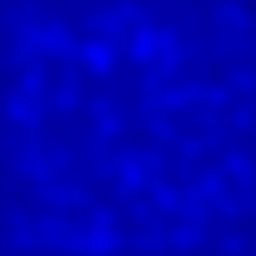

Supplement: S8 File — (ZIP) [file pone.0158590.s008.zip › movie_normal-2-original/movie_normal-2-original107.jpg]
