# Supplementary figures and images for: Invisible Brain: Knowledge in Research Works and Neuron Activity (part 3 of 6)
Source: PLoS One. 2016 Jul 20;11(7):e0158590. doi: 10.1371/journal.pone.0158590 (PMC4954711; doi:10.1371/journal.pone.0158590)

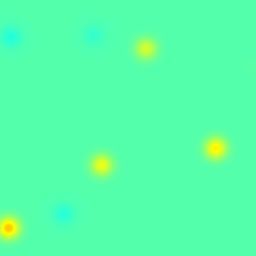

Supplement: S7 File — (ZIP) [file pone.0158590.s007.zip › Gastritis/movie_WoSPubmed_anaphylaxis_Diphtheria_YellowFever_InVitroFertilization_Gastritis908.jpg]

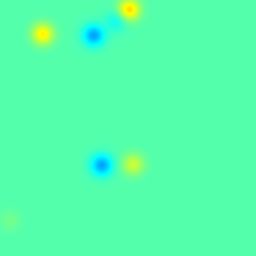

Supplement: S7 File — (ZIP) [file pone.0158590.s007.zip › Gastritis/movie_WoSPubmed_anaphylaxis_Diphtheria_YellowFever_InVitroFertilization_Gastritis910.jpg]

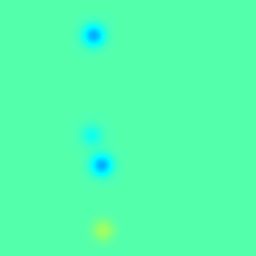

Supplement: S7 File — (ZIP) [file pone.0158590.s007.zip › Gastritis/movie_WoSPubmed_anaphylaxis_Diphtheria_YellowFever_InVitroFertilization_Gastritis911.jpg]

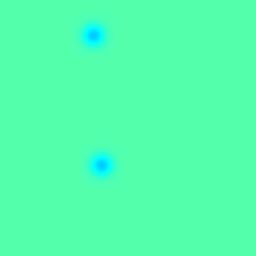

Supplement: S7 File — (ZIP) [file pone.0158590.s007.zip › Gastritis/movie_WoSPubmed_anaphylaxis_Diphtheria_YellowFever_InVitroFertilization_Gastritis913.jpg]

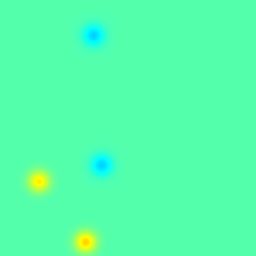

Supplement: S7 File — (ZIP) [file pone.0158590.s007.zip › Gastritis/movie_WoSPubmed_anaphylaxis_Diphtheria_YellowFever_InVitroFertilization_Gastritis914.jpg]

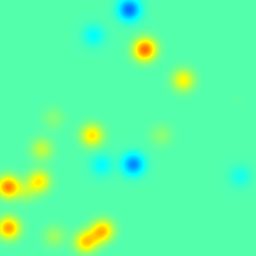

Supplement: S7 File — (ZIP) [file pone.0158590.s007.zip › Gastritis/movie_WoSPubmed_anaphylaxis_Diphtheria_YellowFever_InVitroFertilization_Gastritis915.jpg]

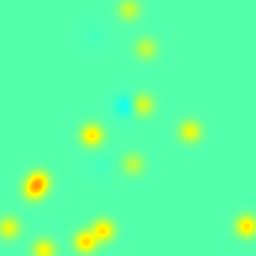

Supplement: S7 File — (ZIP) [file pone.0158590.s007.zip › Gastritis/movie_WoSPubmed_anaphylaxis_Diphtheria_YellowFever_InVitroFertilization_Gastritis916.jpg]

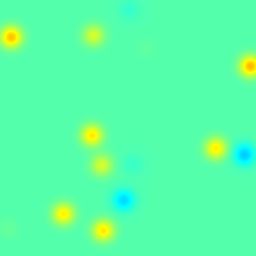

Supplement: S7 File — (ZIP) [file pone.0158590.s007.zip › Gastritis/movie_WoSPubmed_anaphylaxis_Diphtheria_YellowFever_InVitroFertilization_Gastritis917.jpg]

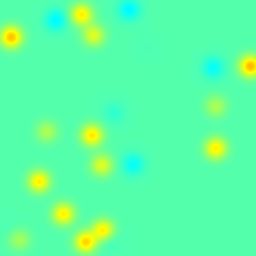

Supplement: S7 File — (ZIP) [file pone.0158590.s007.zip › Gastritis/movie_WoSPubmed_anaphylaxis_Diphtheria_YellowFever_InVitroFertilization_Gastritis918.jpg]

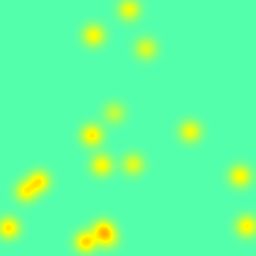

Supplement: S7 File — (ZIP) [file pone.0158590.s007.zip › Gastritis/movie_WoSPubmed_anaphylaxis_Diphtheria_YellowFever_InVitroFertilization_Gastritis919.jpg]

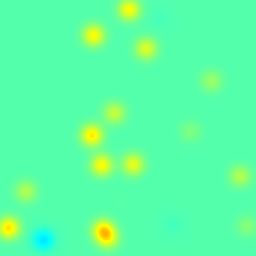

Supplement: S7 File — (ZIP) [file pone.0158590.s007.zip › Gastritis/movie_WoSPubmed_anaphylaxis_Diphtheria_YellowFever_InVitroFertilization_Gastritis920.jpg]

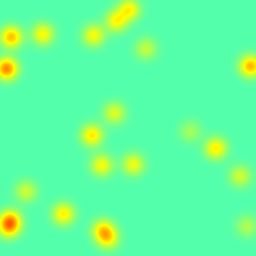

Supplement: S7 File — (ZIP) [file pone.0158590.s007.zip › Gastritis/movie_WoSPubmed_anaphylaxis_Diphtheria_YellowFever_InVitroFertilization_Gastritis921.jpg]

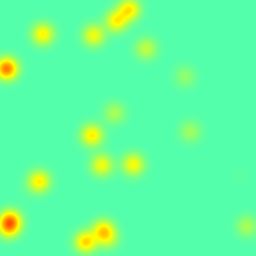

Supplement: S7 File — (ZIP) [file pone.0158590.s007.zip › Gastritis/movie_WoSPubmed_anaphylaxis_Diphtheria_YellowFever_InVitroFertilization_Gastritis922.jpg]

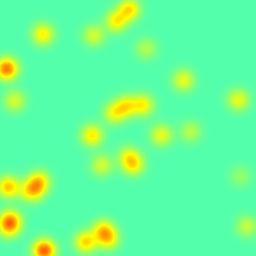

Supplement: S7 File — (ZIP) [file pone.0158590.s007.zip › Gastritis/movie_WoSPubmed_anaphylaxis_Diphtheria_YellowFever_InVitroFertilization_Gastritis923.jpg]

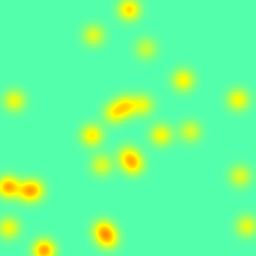

Supplement: S7 File — (ZIP) [file pone.0158590.s007.zip › Gastritis/movie_WoSPubmed_anaphylaxis_Diphtheria_YellowFever_InVitroFertilization_Gastritis924.jpg]

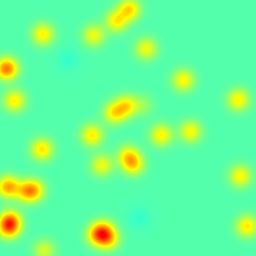

Supplement: S7 File — (ZIP) [file pone.0158590.s007.zip › Gastritis/movie_WoSPubmed_anaphylaxis_Diphtheria_YellowFever_InVitroFertilization_Gastritis925.jpg]

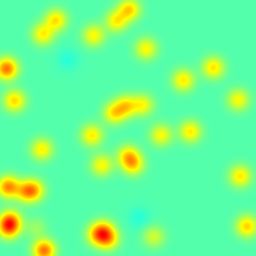

Supplement: S7 File — (ZIP) [file pone.0158590.s007.zip › Gastritis/movie_WoSPubmed_anaphylaxis_Diphtheria_YellowFever_InVitroFertilization_Gastritis926.jpg]

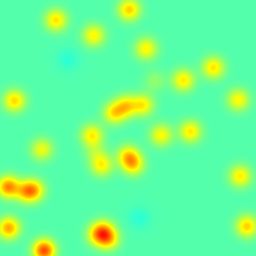

Supplement: S7 File — (ZIP) [file pone.0158590.s007.zip › Gastritis/movie_WoSPubmed_anaphylaxis_Diphtheria_YellowFever_InVitroFertilization_Gastritis927.jpg]

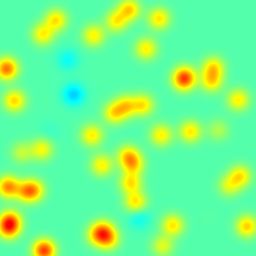

Supplement: S7 File — (ZIP) [file pone.0158590.s007.zip › Gastritis/movie_WoSPubmed_anaphylaxis_Diphtheria_YellowFever_InVitroFertilization_Gastritis928.jpg]

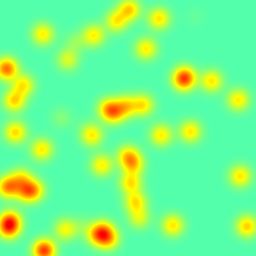

Supplement: S7 File — (ZIP) [file pone.0158590.s007.zip › Gastritis/movie_WoSPubmed_anaphylaxis_Diphtheria_YellowFever_InVitroFertilization_Gastritis929.jpg]

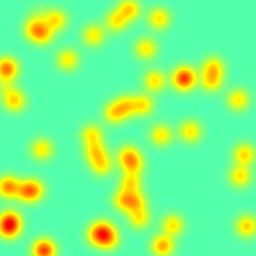

Supplement: S7 File — (ZIP) [file pone.0158590.s007.zip › Gastritis/movie_WoSPubmed_anaphylaxis_Diphtheria_YellowFever_InVitroFertilization_Gastritis930.jpg]

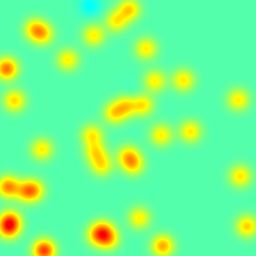

Supplement: S7 File — (ZIP) [file pone.0158590.s007.zip › Gastritis/movie_WoSPubmed_anaphylaxis_Diphtheria_YellowFever_InVitroFertilization_Gastritis931.jpg]

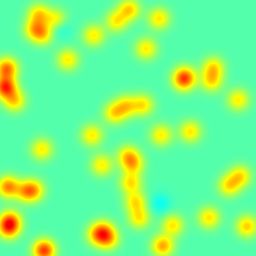

Supplement: S7 File — (ZIP) [file pone.0158590.s007.zip › Gastritis/movie_WoSPubmed_anaphylaxis_Diphtheria_YellowFever_InVitroFertilization_Gastritis932.jpg]

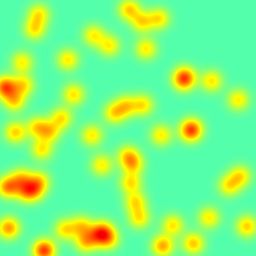

Supplement: S7 File — (ZIP) [file pone.0158590.s007.zip › Gastritis/movie_WoSPubmed_anaphylaxis_Diphtheria_YellowFever_InVitroFertilization_Gastritis933.jpg]

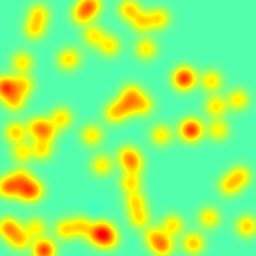

Supplement: S7 File — (ZIP) [file pone.0158590.s007.zip › Gastritis/movie_WoSPubmed_anaphylaxis_Diphtheria_YellowFever_InVitroFertilization_Gastritis934.jpg]

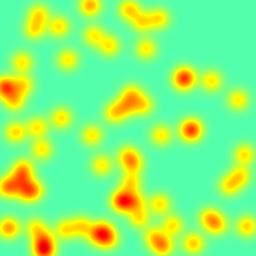

Supplement: S7 File — (ZIP) [file pone.0158590.s007.zip › Gastritis/movie_WoSPubmed_anaphylaxis_Diphtheria_YellowFever_InVitroFertilization_Gastritis935.jpg]

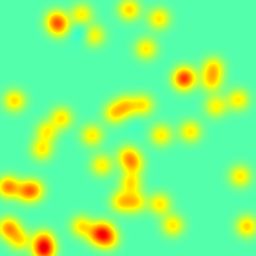

Supplement: S7 File — (ZIP) [file pone.0158590.s007.zip › Gastritis/movie_WoSPubmed_anaphylaxis_Diphtheria_YellowFever_InVitroFertilization_Gastritis936.jpg]

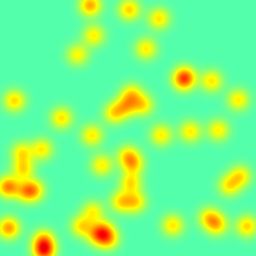

Supplement: S7 File — (ZIP) [file pone.0158590.s007.zip › Gastritis/movie_WoSPubmed_anaphylaxis_Diphtheria_YellowFever_InVitroFertilization_Gastritis937.jpg]

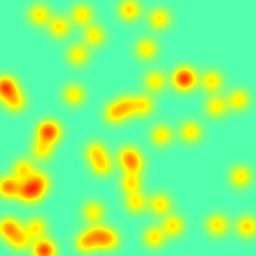

Supplement: S7 File — (ZIP) [file pone.0158590.s007.zip › Gastritis/movie_WoSPubmed_anaphylaxis_Diphtheria_YellowFever_InVitroFertilization_Gastritis938.jpg]

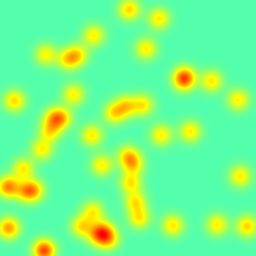

Supplement: S7 File — (ZIP) [file pone.0158590.s007.zip › Gastritis/movie_WoSPubmed_anaphylaxis_Diphtheria_YellowFever_InVitroFertilization_Gastritis939.jpg]

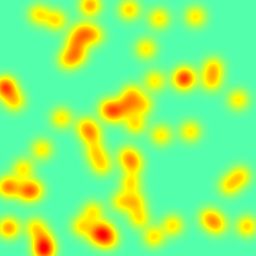

Supplement: S7 File — (ZIP) [file pone.0158590.s007.zip › Gastritis/movie_WoSPubmed_anaphylaxis_Diphtheria_YellowFever_InVitroFertilization_Gastritis940.jpg]

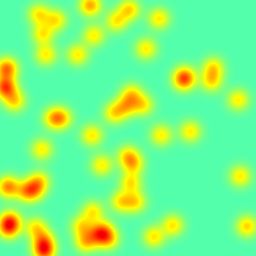

Supplement: S7 File — (ZIP) [file pone.0158590.s007.zip › Gastritis/movie_WoSPubmed_anaphylaxis_Diphtheria_YellowFever_InVitroFertilization_Gastritis941.jpg]

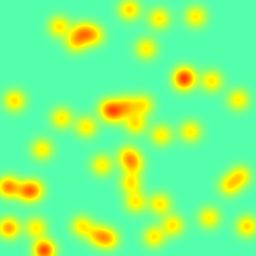

Supplement: S7 File — (ZIP) [file pone.0158590.s007.zip › Gastritis/movie_WoSPubmed_anaphylaxis_Diphtheria_YellowFever_InVitroFertilization_Gastritis942.jpg]

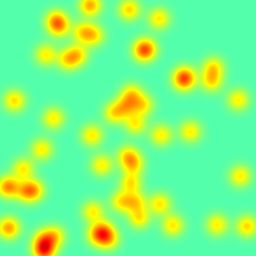

Supplement: S7 File — (ZIP) [file pone.0158590.s007.zip › Gastritis/movie_WoSPubmed_anaphylaxis_Diphtheria_YellowFever_InVitroFertilization_Gastritis943.jpg]

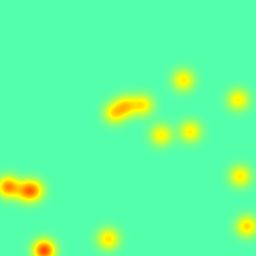

Supplement: S7 File — (ZIP) [file pone.0158590.s007.zip › Gastritis/movie_WoSPubmed_anaphylaxis_Diphtheria_YellowFever_InVitroFertilization_Gastritis944.jpg]

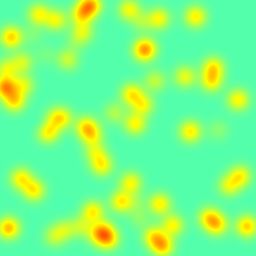

Supplement: S7 File — (ZIP) [file pone.0158590.s007.zip › Gastritis_Wos/movie_WoSPubmed_anaphylaxis_Diphtheria_YellowFever_InVitroFertilization_Gastritis800.jpg]

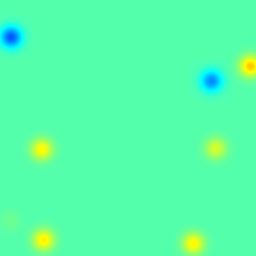

Supplement: S7 File — (ZIP) [file pone.0158590.s007.zip › Gastritis_Wos/movie_WoSPubmed_anaphylaxis_Diphtheria_YellowFever_InVitroFertilization_Gastritis801.jpg]

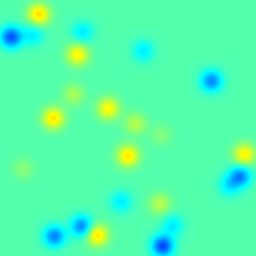

Supplement: S7 File — (ZIP) [file pone.0158590.s007.zip › Gastritis_Wos/movie_WoSPubmed_anaphylaxis_Diphtheria_YellowFever_InVitroFertilization_Gastritis802.jpg]

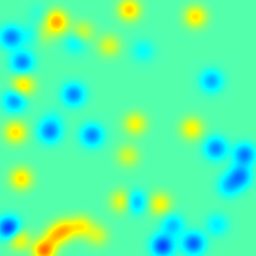

Supplement: S7 File — (ZIP) [file pone.0158590.s007.zip › Gastritis_Wos/movie_WoSPubmed_anaphylaxis_Diphtheria_YellowFever_InVitroFertilization_Gastritis803.jpg]

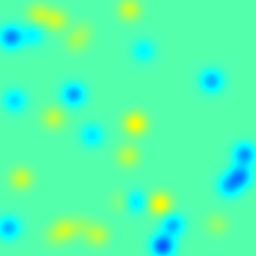

Supplement: S7 File — (ZIP) [file pone.0158590.s007.zip › Gastritis_Wos/movie_WoSPubmed_anaphylaxis_Diphtheria_YellowFever_InVitroFertilization_Gastritis804.jpg]

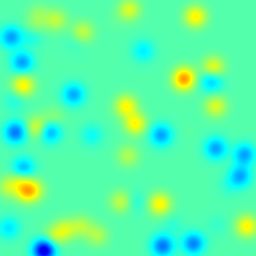

Supplement: S7 File — (ZIP) [file pone.0158590.s007.zip › Gastritis_Wos/movie_WoSPubmed_anaphylaxis_Diphtheria_YellowFever_InVitroFertilization_Gastritis805.jpg]

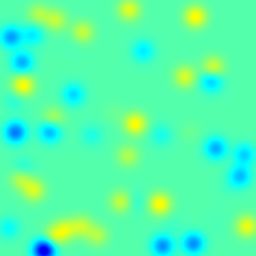

Supplement: S7 File — (ZIP) [file pone.0158590.s007.zip › Gastritis_Wos/movie_WoSPubmed_anaphylaxis_Diphtheria_YellowFever_InVitroFertilization_Gastritis806.jpg]

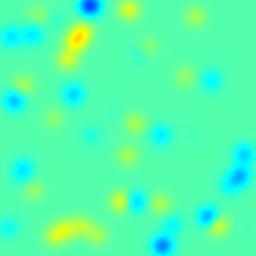

Supplement: S7 File — (ZIP) [file pone.0158590.s007.zip › Gastritis_Wos/movie_WoSPubmed_anaphylaxis_Diphtheria_YellowFever_InVitroFertilization_Gastritis807.jpg]

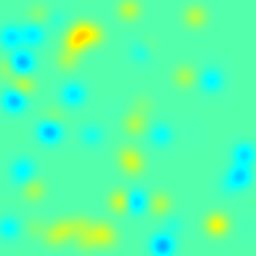

Supplement: S7 File — (ZIP) [file pone.0158590.s007.zip › Gastritis_Wos/movie_WoSPubmed_anaphylaxis_Diphtheria_YellowFever_InVitroFertilization_Gastritis808.jpg]

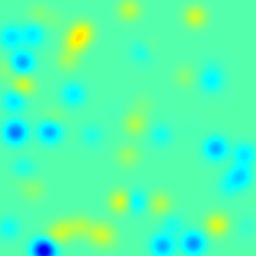

Supplement: S7 File — (ZIP) [file pone.0158590.s007.zip › Gastritis_Wos/movie_WoSPubmed_anaphylaxis_Diphtheria_YellowFever_InVitroFertilization_Gastritis809.jpg]

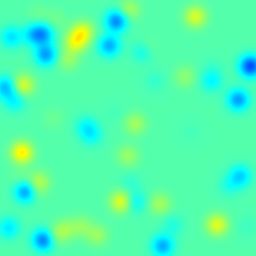

Supplement: S7 File — (ZIP) [file pone.0158590.s007.zip › Gastritis_Wos/movie_WoSPubmed_anaphylaxis_Diphtheria_YellowFever_InVitroFertilization_Gastritis810.jpg]

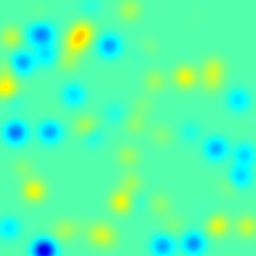

Supplement: S7 File — (ZIP) [file pone.0158590.s007.zip › Gastritis_Wos/movie_WoSPubmed_anaphylaxis_Diphtheria_YellowFever_InVitroFertilization_Gastritis811.jpg]

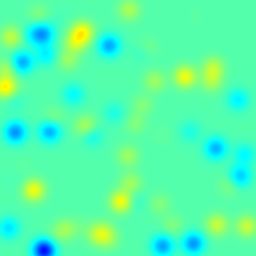

Supplement: S7 File — (ZIP) [file pone.0158590.s007.zip › Gastritis_Wos/movie_WoSPubmed_anaphylaxis_Diphtheria_YellowFever_InVitroFertilization_Gastritis812.jpg]

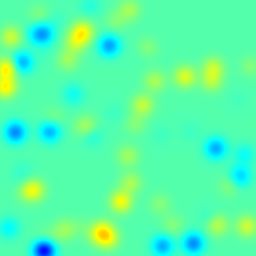

Supplement: S7 File — (ZIP) [file pone.0158590.s007.zip › Gastritis_Wos/movie_WoSPubmed_anaphylaxis_Diphtheria_YellowFever_InVitroFertilization_Gastritis813.jpg]

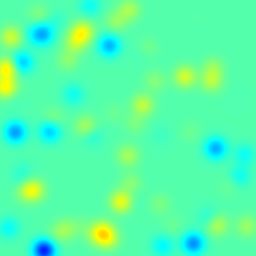

Supplement: S7 File — (ZIP) [file pone.0158590.s007.zip › Gastritis_Wos/movie_WoSPubmed_anaphylaxis_Diphtheria_YellowFever_InVitroFertilization_Gastritis814.jpg]

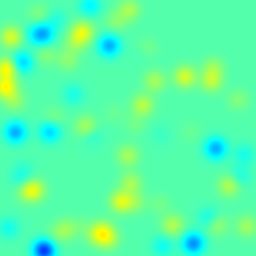

Supplement: S7 File — (ZIP) [file pone.0158590.s007.zip › Gastritis_Wos/movie_WoSPubmed_anaphylaxis_Diphtheria_YellowFever_InVitroFertilization_Gastritis815.jpg]

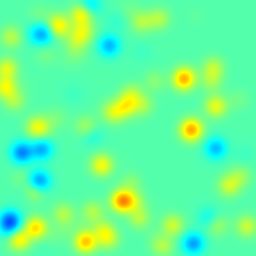

Supplement: S7 File — (ZIP) [file pone.0158590.s007.zip › Gastritis_Wos/movie_WoSPubmed_anaphylaxis_Diphtheria_YellowFever_InVitroFertilization_Gastritis816.jpg]

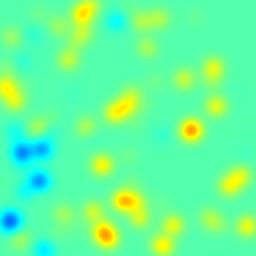

Supplement: S7 File — (ZIP) [file pone.0158590.s007.zip › Gastritis_Wos/movie_WoSPubmed_anaphylaxis_Diphtheria_YellowFever_InVitroFertilization_Gastritis817.jpg]

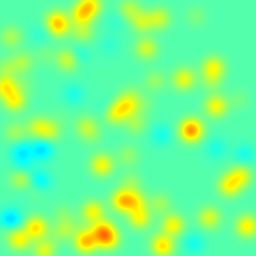

Supplement: S7 File — (ZIP) [file pone.0158590.s007.zip › Gastritis_Wos/movie_WoSPubmed_anaphylaxis_Diphtheria_YellowFever_InVitroFertilization_Gastritis818.jpg]

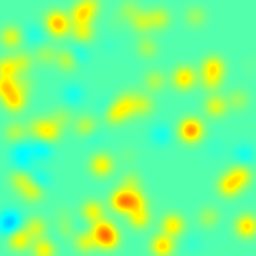

Supplement: S7 File — (ZIP) [file pone.0158590.s007.zip › Gastritis_Wos/movie_WoSPubmed_anaphylaxis_Diphtheria_YellowFever_InVitroFertilization_Gastritis819.jpg]

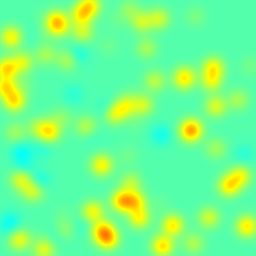

Supplement: S7 File — (ZIP) [file pone.0158590.s007.zip › Gastritis_Wos/movie_WoSPubmed_anaphylaxis_Diphtheria_YellowFever_InVitroFertilization_Gastritis820.jpg]

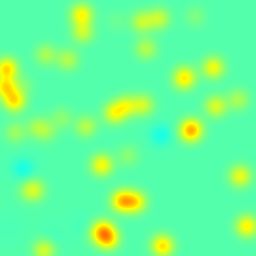

Supplement: S7 File — (ZIP) [file pone.0158590.s007.zip › Gastritis_Wos/movie_WoSPubmed_anaphylaxis_Diphtheria_YellowFever_InVitroFertilization_Gastritis821.jpg]

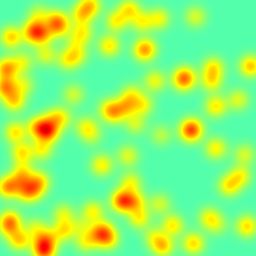

Supplement: S7 File — (ZIP) [file pone.0158590.s007.zip › Gastritis_Wos/movie_WoSPubmed_anaphylaxis_Diphtheria_YellowFever_InVitroFertilization_Gastritis822.jpg]

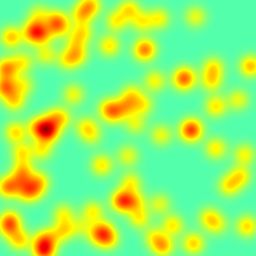

Supplement: S7 File — (ZIP) [file pone.0158590.s007.zip › Gastritis_Wos/movie_WoSPubmed_anaphylaxis_Diphtheria_YellowFever_InVitroFertilization_Gastritis823.jpg]

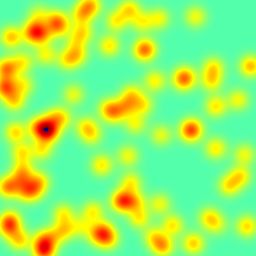

Supplement: S7 File — (ZIP) [file pone.0158590.s007.zip › Gastritis_Wos/movie_WoSPubmed_anaphylaxis_Diphtheria_YellowFever_InVitroFertilization_Gastritis824.jpg]

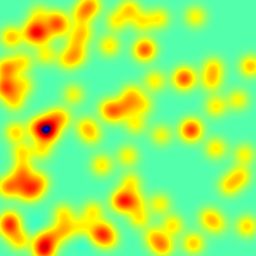

Supplement: S7 File — (ZIP) [file pone.0158590.s007.zip › Gastritis_Wos/movie_WoSPubmed_anaphylaxis_Diphtheria_YellowFever_InVitroFertilization_Gastritis825.jpg]

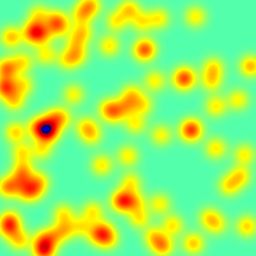

Supplement: S7 File — (ZIP) [file pone.0158590.s007.zip › Gastritis_Wos/movie_WoSPubmed_anaphylaxis_Diphtheria_YellowFever_InVitroFertilization_Gastritis826.jpg]

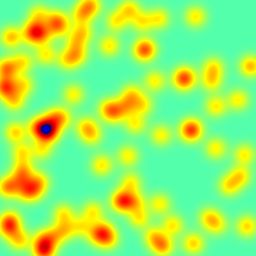

Supplement: S7 File — (ZIP) [file pone.0158590.s007.zip › Gastritis_Wos/movie_WoSPubmed_anaphylaxis_Diphtheria_YellowFever_InVitroFertilization_Gastritis827.jpg]

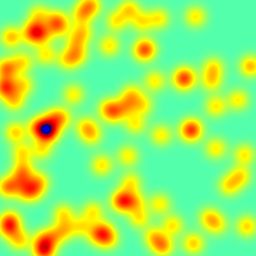

Supplement: S7 File — (ZIP) [file pone.0158590.s007.zip › Gastritis_Wos/movie_WoSPubmed_anaphylaxis_Diphtheria_YellowFever_InVitroFertilization_Gastritis828.jpg]

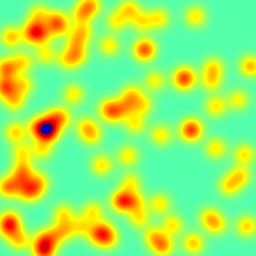

Supplement: S7 File — (ZIP) [file pone.0158590.s007.zip › Gastritis_Wos/movie_WoSPubmed_anaphylaxis_Diphtheria_YellowFever_InVitroFertilization_Gastritis829.jpg]

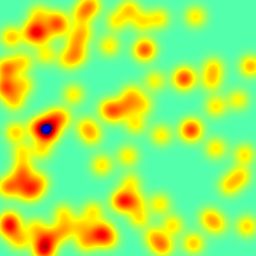

Supplement: S7 File — (ZIP) [file pone.0158590.s007.zip › Gastritis_Wos/movie_WoSPubmed_anaphylaxis_Diphtheria_YellowFever_InVitroFertilization_Gastritis830.jpg]

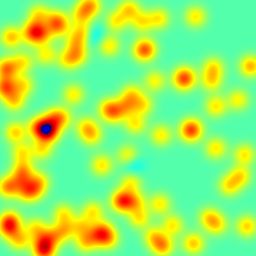

Supplement: S7 File — (ZIP) [file pone.0158590.s007.zip › Gastritis_Wos/movie_WoSPubmed_anaphylaxis_Diphtheria_YellowFever_InVitroFertilization_Gastritis831.jpg]

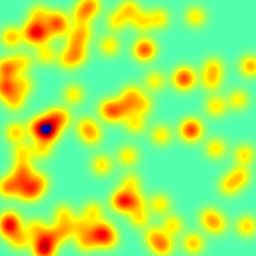

Supplement: S7 File — (ZIP) [file pone.0158590.s007.zip › Gastritis_Wos/movie_WoSPubmed_anaphylaxis_Diphtheria_YellowFever_InVitroFertilization_Gastritis832.jpg]

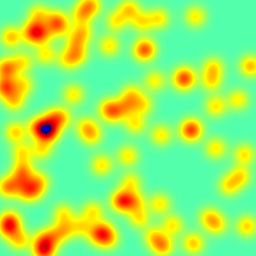

Supplement: S7 File — (ZIP) [file pone.0158590.s007.zip › Gastritis_Wos/movie_WoSPubmed_anaphylaxis_Diphtheria_YellowFever_InVitroFertilization_Gastritis833.jpg]

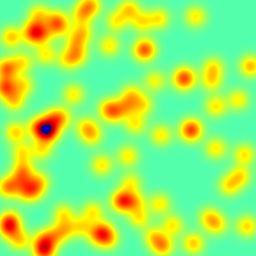

Supplement: S7 File — (ZIP) [file pone.0158590.s007.zip › Gastritis_Wos/movie_WoSPubmed_anaphylaxis_Diphtheria_YellowFever_InVitroFertilization_Gastritis834.jpg]

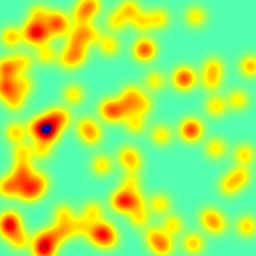

Supplement: S7 File — (ZIP) [file pone.0158590.s007.zip › Gastritis_Wos/movie_WoSPubmed_anaphylaxis_Diphtheria_YellowFever_InVitroFertilization_Gastritis835.jpg]

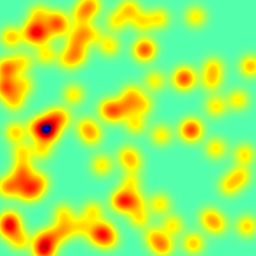

Supplement: S7 File — (ZIP) [file pone.0158590.s007.zip › Gastritis_Wos/movie_WoSPubmed_anaphylaxis_Diphtheria_YellowFever_InVitroFertilization_Gastritis836.jpg]

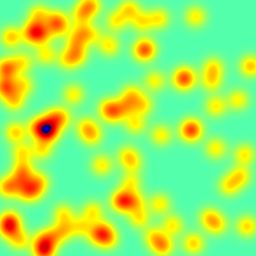

Supplement: S7 File — (ZIP) [file pone.0158590.s007.zip › Gastritis_Wos/movie_WoSPubmed_anaphylaxis_Diphtheria_YellowFever_InVitroFertilization_Gastritis837.jpg]

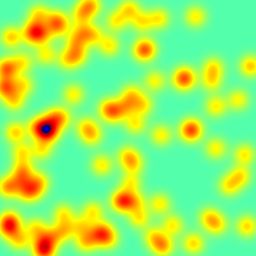

Supplement: S7 File — (ZIP) [file pone.0158590.s007.zip › Gastritis_Wos/movie_WoSPubmed_anaphylaxis_Diphtheria_YellowFever_InVitroFertilization_Gastritis838.jpg]

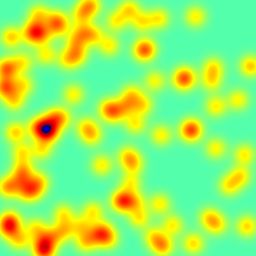

Supplement: S7 File — (ZIP) [file pone.0158590.s007.zip › Gastritis_Wos/movie_WoSPubmed_anaphylaxis_Diphtheria_YellowFever_InVitroFertilization_Gastritis839.jpg]

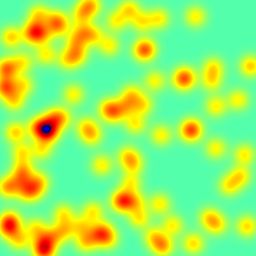

Supplement: S7 File — (ZIP) [file pone.0158590.s007.zip › Gastritis_Wos/movie_WoSPubmed_anaphylaxis_Diphtheria_YellowFever_InVitroFertilization_Gastritis840.jpg]

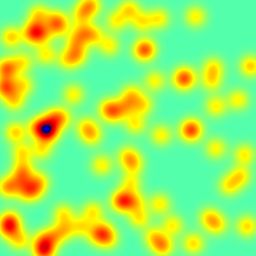

Supplement: S7 File — (ZIP) [file pone.0158590.s007.zip › Gastritis_Wos/movie_WoSPubmed_anaphylaxis_Diphtheria_YellowFever_InVitroFertilization_Gastritis841.jpg]

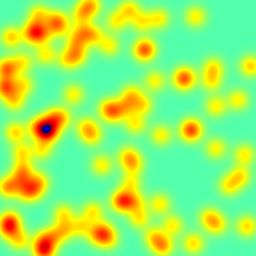

Supplement: S7 File — (ZIP) [file pone.0158590.s007.zip › Gastritis_Wos/movie_WoSPubmed_anaphylaxis_Diphtheria_YellowFever_InVitroFertilization_Gastritis842.jpg]

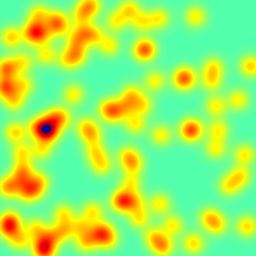

Supplement: S7 File — (ZIP) [file pone.0158590.s007.zip › Gastritis_Wos/movie_WoSPubmed_anaphylaxis_Diphtheria_YellowFever_InVitroFertilization_Gastritis843.jpg]

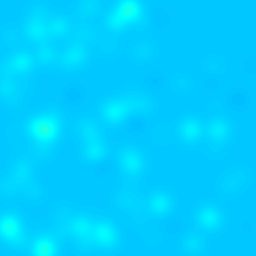

Supplement: S7 File — (ZIP) [file pone.0158590.s007.zip › iNet1_Size100_CC01inh/movie_iNet1_Size100_CC01inh_anaphylaxis_765_top100817.jpg]

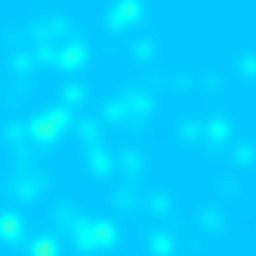

Supplement: S7 File — (ZIP) [file pone.0158590.s007.zip › iNet1_Size100_CC01inh/movie_iNet1_Size100_CC01inh_anaphylaxis_765_top100818.jpg]

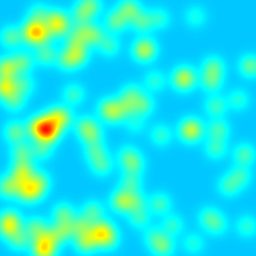

Supplement: S7 File — (ZIP) [file pone.0158590.s007.zip › iNet1_Size100_CC01inh/movie_iNet1_Size100_CC01inh_anaphylaxis_765_top100819.jpg]

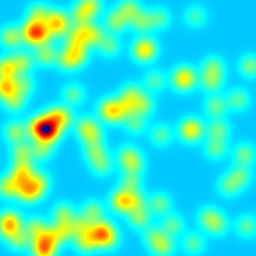

Supplement: S7 File — (ZIP) [file pone.0158590.s007.zip › iNet1_Size100_CC01inh/movie_iNet1_Size100_CC01inh_anaphylaxis_765_top100820.jpg]

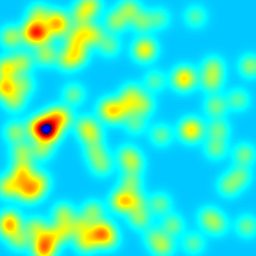

Supplement: S7 File — (ZIP) [file pone.0158590.s007.zip › iNet1_Size100_CC01inh/movie_iNet1_Size100_CC01inh_anaphylaxis_765_top100821.jpg]

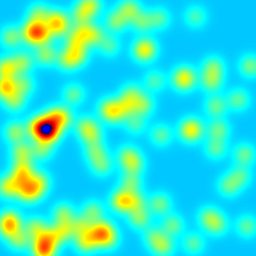

Supplement: S7 File — (ZIP) [file pone.0158590.s007.zip › iNet1_Size100_CC01inh/movie_iNet1_Size100_CC01inh_anaphylaxis_765_top100822.jpg]

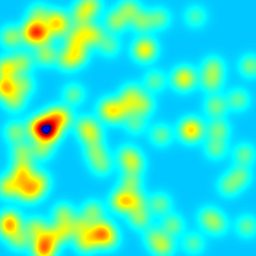

Supplement: S7 File — (ZIP) [file pone.0158590.s007.zip › iNet1_Size100_CC01inh/movie_iNet1_Size100_CC01inh_anaphylaxis_765_top100823.jpg]

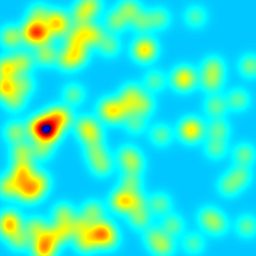

Supplement: S7 File — (ZIP) [file pone.0158590.s007.zip › iNet1_Size100_CC01inh/movie_iNet1_Size100_CC01inh_anaphylaxis_765_top100824.jpg]

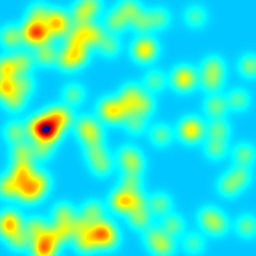

Supplement: S7 File — (ZIP) [file pone.0158590.s007.zip › iNet1_Size100_CC01inh/movie_iNet1_Size100_CC01inh_anaphylaxis_765_top100825.jpg]

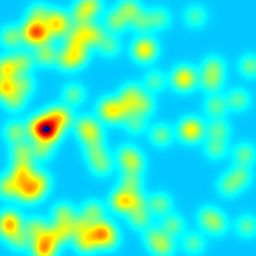

Supplement: S7 File — (ZIP) [file pone.0158590.s007.zip › iNet1_Size100_CC01inh/movie_iNet1_Size100_CC01inh_anaphylaxis_765_top100826.jpg]

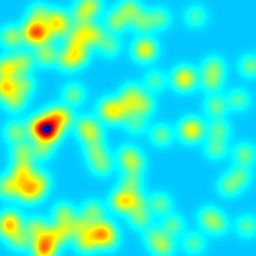

Supplement: S7 File — (ZIP) [file pone.0158590.s007.zip › iNet1_Size100_CC01inh/movie_iNet1_Size100_CC01inh_anaphylaxis_765_top100827.jpg]

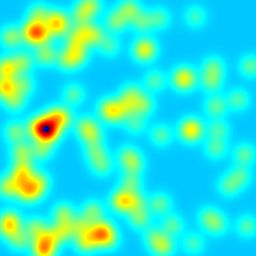

Supplement: S7 File — (ZIP) [file pone.0158590.s007.zip › iNet1_Size100_CC01inh/movie_iNet1_Size100_CC01inh_anaphylaxis_765_top100828.jpg]

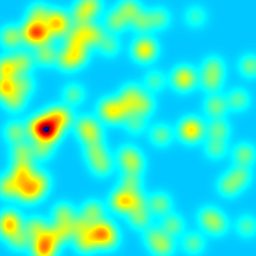

Supplement: S7 File — (ZIP) [file pone.0158590.s007.zip › iNet1_Size100_CC01inh/movie_iNet1_Size100_CC01inh_anaphylaxis_765_top100829.jpg]

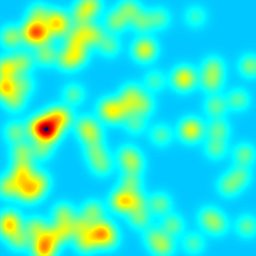

Supplement: S7 File — (ZIP) [file pone.0158590.s007.zip › iNet1_Size100_CC01inh/movie_iNet1_Size100_CC01inh_anaphylaxis_765_top100830.jpg]

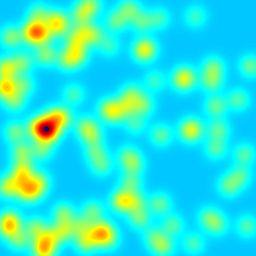

Supplement: S7 File — (ZIP) [file pone.0158590.s007.zip › iNet1_Size100_CC01inh/movie_iNet1_Size100_CC01inh_anaphylaxis_765_top100831.jpg]

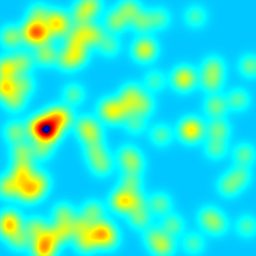

Supplement: S7 File — (ZIP) [file pone.0158590.s007.zip › iNet1_Size100_CC01inh/movie_iNet1_Size100_CC01inh_anaphylaxis_765_top100832.jpg]

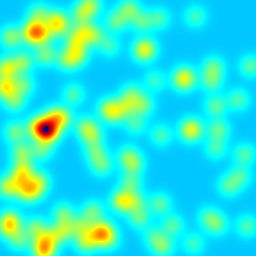

Supplement: S7 File — (ZIP) [file pone.0158590.s007.zip › iNet1_Size100_CC01inh/movie_iNet1_Size100_CC01inh_anaphylaxis_765_top100833.jpg]

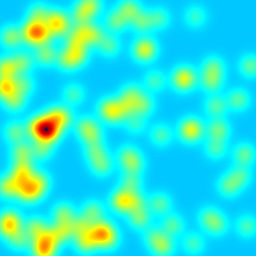

Supplement: S7 File — (ZIP) [file pone.0158590.s007.zip › iNet1_Size100_CC01inh/movie_iNet1_Size100_CC01inh_anaphylaxis_765_top100834.jpg]

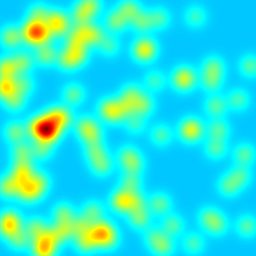

Supplement: S7 File — (ZIP) [file pone.0158590.s007.zip › iNet1_Size100_CC01inh/movie_iNet1_Size100_CC01inh_anaphylaxis_765_top100835.jpg]

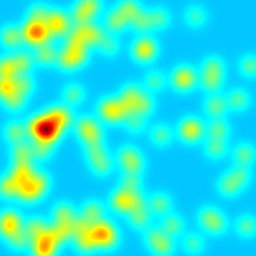

Supplement: S7 File — (ZIP) [file pone.0158590.s007.zip › iNet1_Size100_CC01inh/movie_iNet1_Size100_CC01inh_anaphylaxis_765_top100836.jpg]

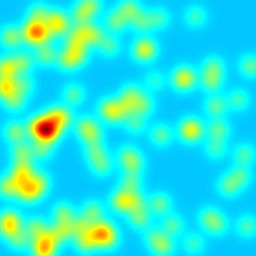

Supplement: S7 File — (ZIP) [file pone.0158590.s007.zip › iNet1_Size100_CC01inh/movie_iNet1_Size100_CC01inh_anaphylaxis_765_top100837.jpg]
