# Supplementary figures and images for: Invisible Brain: Knowledge in Research Works and Neuron Activity (part 6 of 6)
Source: PLoS One. 2016 Jul 20;11(7):e0158590. doi: 10.1371/journal.pone.0158590 (PMC4954711; doi:10.1371/journal.pone.0158590)

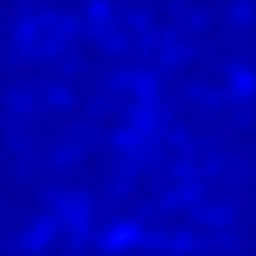

Supplement: S8 File — (ZIP) [file pone.0158590.s008.zip › movie_normal-2-original/movie_normal-2-original199.jpg]

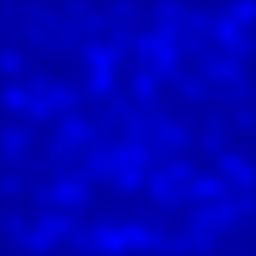

Supplement: S8 File — (ZIP) [file pone.0158590.s008.zip › movie_normal-2-original/movie_normal-2-original2.jpg]

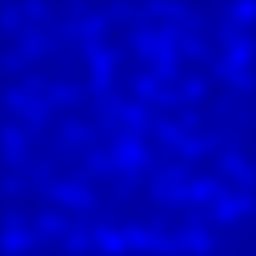

Supplement: S8 File — (ZIP) [file pone.0158590.s008.zip › movie_normal-2-original/movie_normal-2-original20.jpg]

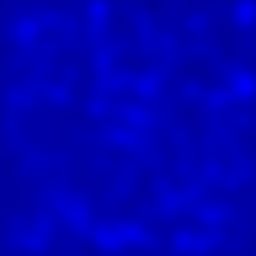

Supplement: S8 File — (ZIP) [file pone.0158590.s008.zip › movie_normal-2-original/movie_normal-2-original200.jpg]

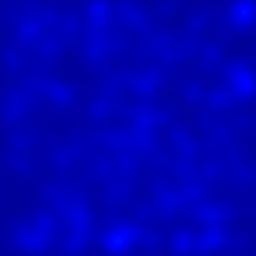

Supplement: S8 File — (ZIP) [file pone.0158590.s008.zip › movie_normal-2-original/movie_normal-2-original201.jpg]

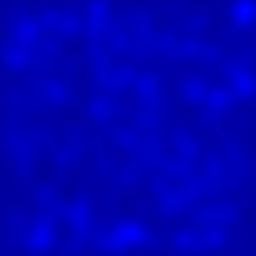

Supplement: S8 File — (ZIP) [file pone.0158590.s008.zip › movie_normal-2-original/movie_normal-2-original202.jpg]

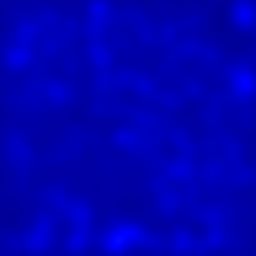

Supplement: S8 File — (ZIP) [file pone.0158590.s008.zip › movie_normal-2-original/movie_normal-2-original203.jpg]

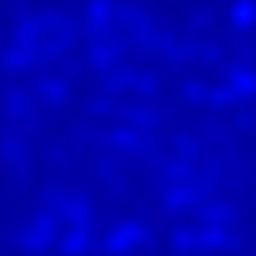

Supplement: S8 File — (ZIP) [file pone.0158590.s008.zip › movie_normal-2-original/movie_normal-2-original204.jpg]

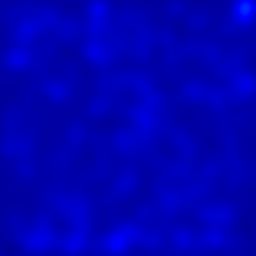

Supplement: S8 File — (ZIP) [file pone.0158590.s008.zip › movie_normal-2-original/movie_normal-2-original205.jpg]

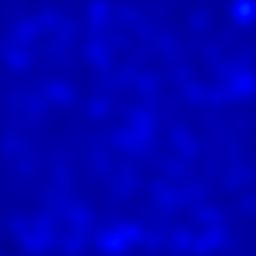

Supplement: S8 File — (ZIP) [file pone.0158590.s008.zip › movie_normal-2-original/movie_normal-2-original206.jpg]

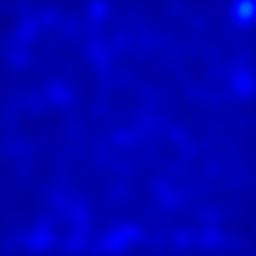

Supplement: S8 File — (ZIP) [file pone.0158590.s008.zip › movie_normal-2-original/movie_normal-2-original207.jpg]

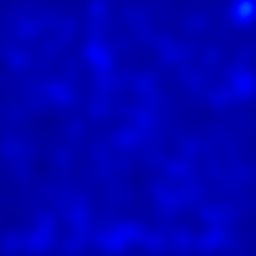

Supplement: S8 File — (ZIP) [file pone.0158590.s008.zip › movie_normal-2-original/movie_normal-2-original208.jpg]

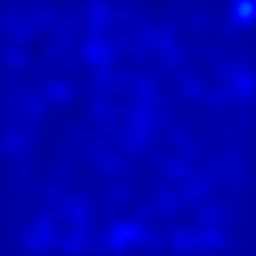

Supplement: S8 File — (ZIP) [file pone.0158590.s008.zip › movie_normal-2-original/movie_normal-2-original209.jpg]

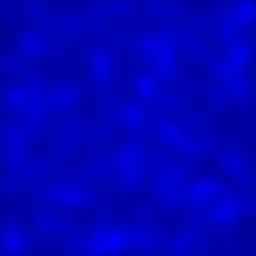

Supplement: S8 File — (ZIP) [file pone.0158590.s008.zip › movie_normal-2-original/movie_normal-2-original21.jpg]

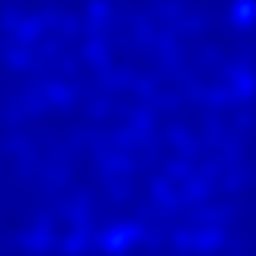

Supplement: S8 File — (ZIP) [file pone.0158590.s008.zip › movie_normal-2-original/movie_normal-2-original210.jpg]

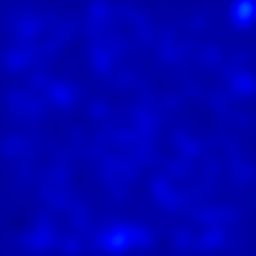

Supplement: S8 File — (ZIP) [file pone.0158590.s008.zip › movie_normal-2-original/movie_normal-2-original211.jpg]

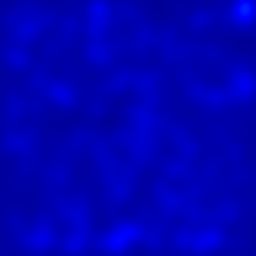

Supplement: S8 File — (ZIP) [file pone.0158590.s008.zip › movie_normal-2-original/movie_normal-2-original212.jpg]

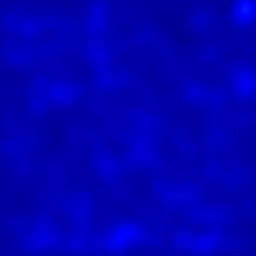

Supplement: S8 File — (ZIP) [file pone.0158590.s008.zip › movie_normal-2-original/movie_normal-2-original213.jpg]

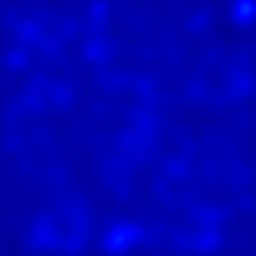

Supplement: S8 File — (ZIP) [file pone.0158590.s008.zip › movie_normal-2-original/movie_normal-2-original214.jpg]

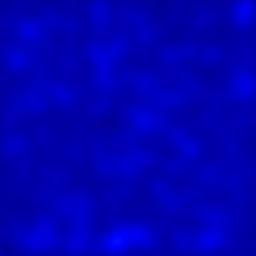

Supplement: S8 File — (ZIP) [file pone.0158590.s008.zip › movie_normal-2-original/movie_normal-2-original215.jpg]

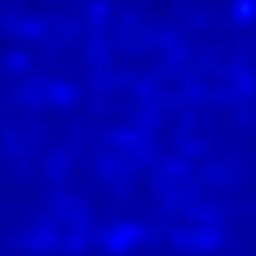

Supplement: S8 File — (ZIP) [file pone.0158590.s008.zip › movie_normal-2-original/movie_normal-2-original216.jpg]

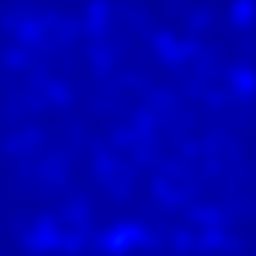

Supplement: S8 File — (ZIP) [file pone.0158590.s008.zip › movie_normal-2-original/movie_normal-2-original217.jpg]

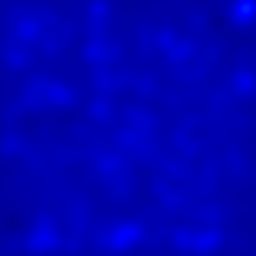

Supplement: S8 File — (ZIP) [file pone.0158590.s008.zip › movie_normal-2-original/movie_normal-2-original218.jpg]

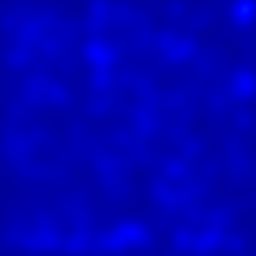

Supplement: S8 File — (ZIP) [file pone.0158590.s008.zip › movie_normal-2-original/movie_normal-2-original219.jpg]

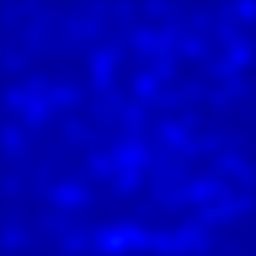

Supplement: S8 File — (ZIP) [file pone.0158590.s008.zip › movie_normal-2-original/movie_normal-2-original22.jpg]

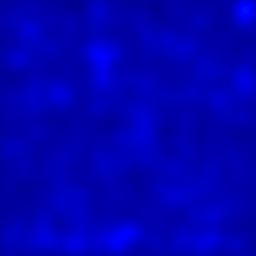

Supplement: S8 File — (ZIP) [file pone.0158590.s008.zip › movie_normal-2-original/movie_normal-2-original220.jpg]

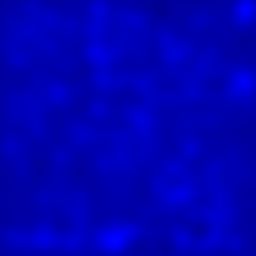

Supplement: S8 File — (ZIP) [file pone.0158590.s008.zip › movie_normal-2-original/movie_normal-2-original221.jpg]

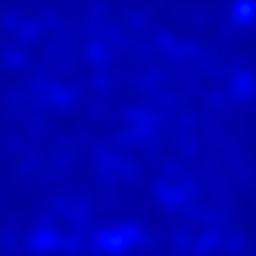

Supplement: S8 File — (ZIP) [file pone.0158590.s008.zip › movie_normal-2-original/movie_normal-2-original222.jpg]

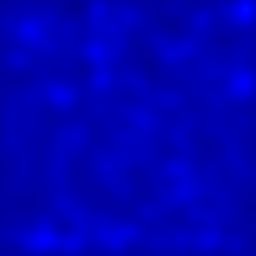

Supplement: S8 File — (ZIP) [file pone.0158590.s008.zip › movie_normal-2-original/movie_normal-2-original223.jpg]

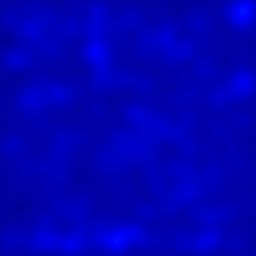

Supplement: S8 File — (ZIP) [file pone.0158590.s008.zip › movie_normal-2-original/movie_normal-2-original224.jpg]

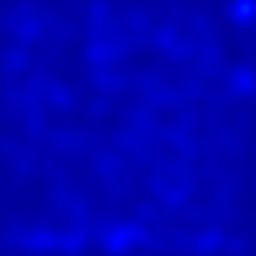

Supplement: S8 File — (ZIP) [file pone.0158590.s008.zip › movie_normal-2-original/movie_normal-2-original225.jpg]

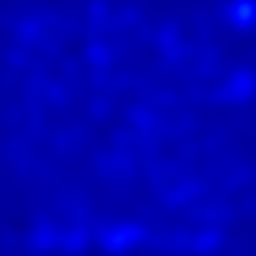

Supplement: S8 File — (ZIP) [file pone.0158590.s008.zip › movie_normal-2-original/movie_normal-2-original226.jpg]

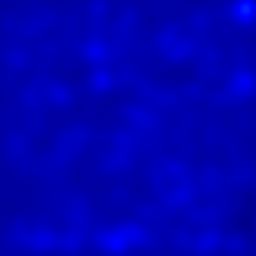

Supplement: S8 File — (ZIP) [file pone.0158590.s008.zip › movie_normal-2-original/movie_normal-2-original227.jpg]

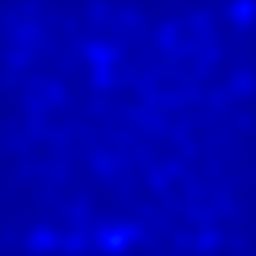

Supplement: S8 File — (ZIP) [file pone.0158590.s008.zip › movie_normal-2-original/movie_normal-2-original228.jpg]

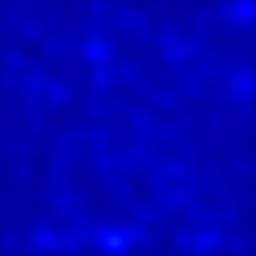

Supplement: S8 File — (ZIP) [file pone.0158590.s008.zip › movie_normal-2-original/movie_normal-2-original229.jpg]

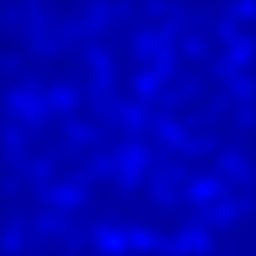

Supplement: S8 File — (ZIP) [file pone.0158590.s008.zip › movie_normal-2-original/movie_normal-2-original23.jpg]

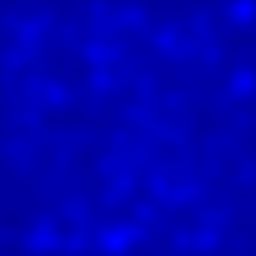

Supplement: S8 File — (ZIP) [file pone.0158590.s008.zip › movie_normal-2-original/movie_normal-2-original230.jpg]

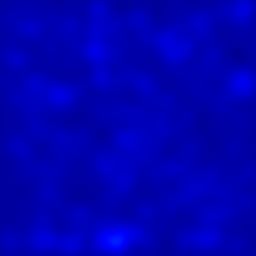

Supplement: S8 File — (ZIP) [file pone.0158590.s008.zip › movie_normal-2-original/movie_normal-2-original231.jpg]

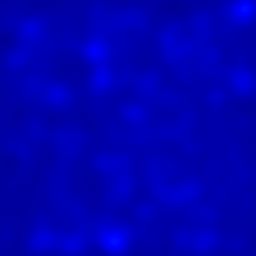

Supplement: S8 File — (ZIP) [file pone.0158590.s008.zip › movie_normal-2-original/movie_normal-2-original232.jpg]

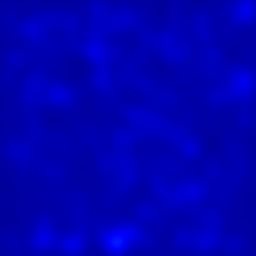

Supplement: S8 File — (ZIP) [file pone.0158590.s008.zip › movie_normal-2-original/movie_normal-2-original233.jpg]

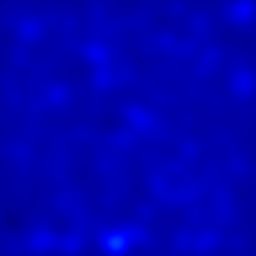

Supplement: S8 File — (ZIP) [file pone.0158590.s008.zip › movie_normal-2-original/movie_normal-2-original234.jpg]

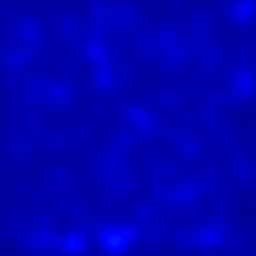

Supplement: S8 File — (ZIP) [file pone.0158590.s008.zip › movie_normal-2-original/movie_normal-2-original235.jpg]

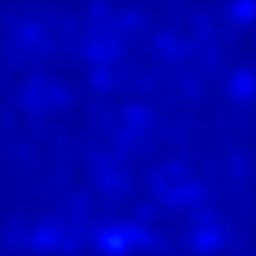

Supplement: S8 File — (ZIP) [file pone.0158590.s008.zip › movie_normal-2-original/movie_normal-2-original236.jpg]

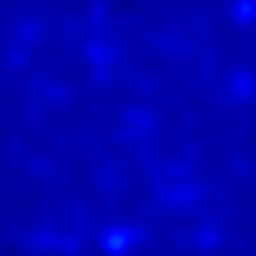

Supplement: S8 File — (ZIP) [file pone.0158590.s008.zip › movie_normal-2-original/movie_normal-2-original237.jpg]

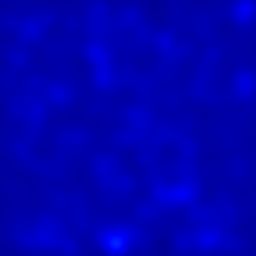

Supplement: S8 File — (ZIP) [file pone.0158590.s008.zip › movie_normal-2-original/movie_normal-2-original238.jpg]

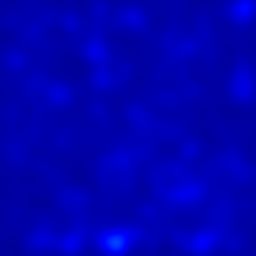

Supplement: S8 File — (ZIP) [file pone.0158590.s008.zip › movie_normal-2-original/movie_normal-2-original239.jpg]

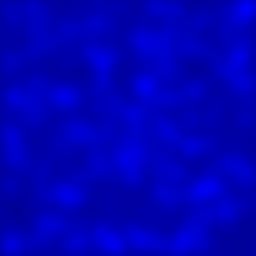

Supplement: S8 File — (ZIP) [file pone.0158590.s008.zip › movie_normal-2-original/movie_normal-2-original24.jpg]

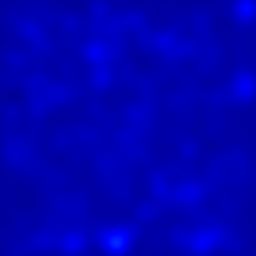

Supplement: S8 File — (ZIP) [file pone.0158590.s008.zip › movie_normal-2-original/movie_normal-2-original240.jpg]

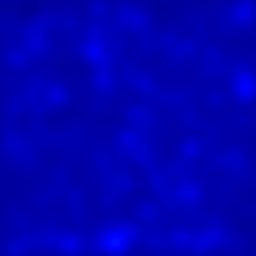

Supplement: S8 File — (ZIP) [file pone.0158590.s008.zip › movie_normal-2-original/movie_normal-2-original241.jpg]

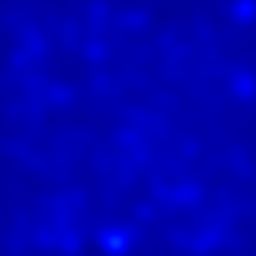

Supplement: S8 File — (ZIP) [file pone.0158590.s008.zip › movie_normal-2-original/movie_normal-2-original242.jpg]

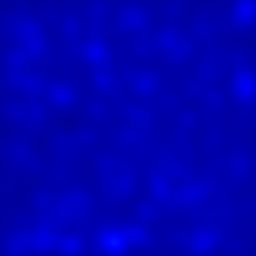

Supplement: S8 File — (ZIP) [file pone.0158590.s008.zip › movie_normal-2-original/movie_normal-2-original243.jpg]

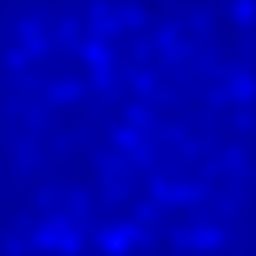

Supplement: S8 File — (ZIP) [file pone.0158590.s008.zip › movie_normal-2-original/movie_normal-2-original244.jpg]

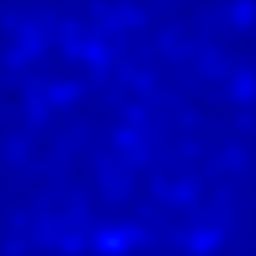

Supplement: S8 File — (ZIP) [file pone.0158590.s008.zip › movie_normal-2-original/movie_normal-2-original245.jpg]

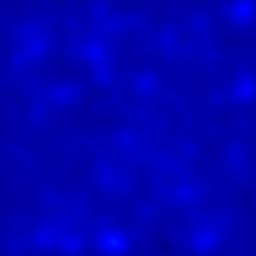

Supplement: S8 File — (ZIP) [file pone.0158590.s008.zip › movie_normal-2-original/movie_normal-2-original246.jpg]

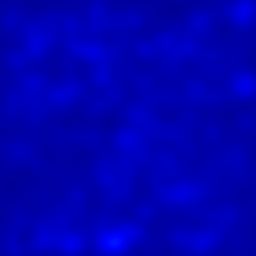

Supplement: S8 File — (ZIP) [file pone.0158590.s008.zip › movie_normal-2-original/movie_normal-2-original247.jpg]

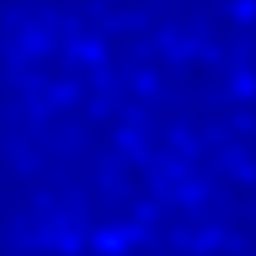

Supplement: S8 File — (ZIP) [file pone.0158590.s008.zip › movie_normal-2-original/movie_normal-2-original248.jpg]

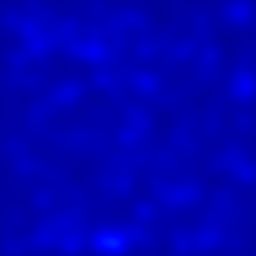

Supplement: S8 File — (ZIP) [file pone.0158590.s008.zip › movie_normal-2-original/movie_normal-2-original249.jpg]

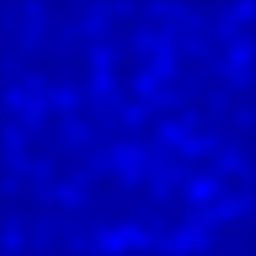

Supplement: S8 File — (ZIP) [file pone.0158590.s008.zip › movie_normal-2-original/movie_normal-2-original25.jpg]

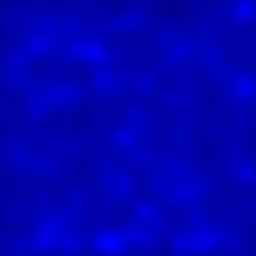

Supplement: S8 File — (ZIP) [file pone.0158590.s008.zip › movie_normal-2-original/movie_normal-2-original250.jpg]

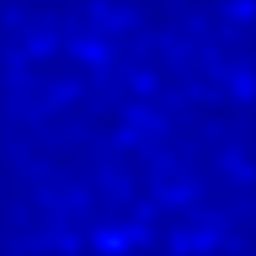

Supplement: S8 File — (ZIP) [file pone.0158590.s008.zip › movie_normal-2-original/movie_normal-2-original251.jpg]

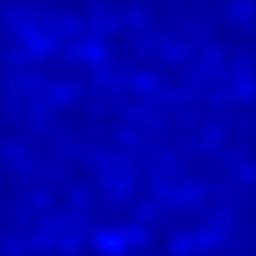

Supplement: S8 File — (ZIP) [file pone.0158590.s008.zip › movie_normal-2-original/movie_normal-2-original252.jpg]

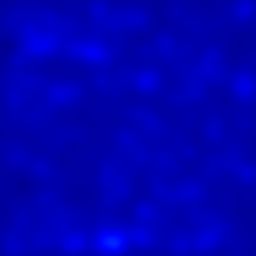

Supplement: S8 File — (ZIP) [file pone.0158590.s008.zip › movie_normal-2-original/movie_normal-2-original253.jpg]

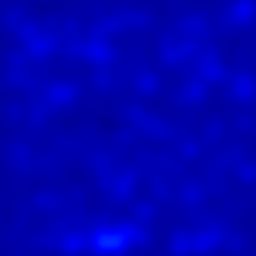

Supplement: S8 File — (ZIP) [file pone.0158590.s008.zip › movie_normal-2-original/movie_normal-2-original254.jpg]

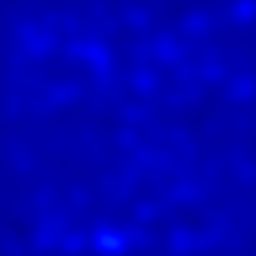

Supplement: S8 File — (ZIP) [file pone.0158590.s008.zip › movie_normal-2-original/movie_normal-2-original255.jpg]

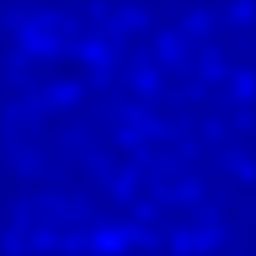

Supplement: S8 File — (ZIP) [file pone.0158590.s008.zip › movie_normal-2-original/movie_normal-2-original256.jpg]

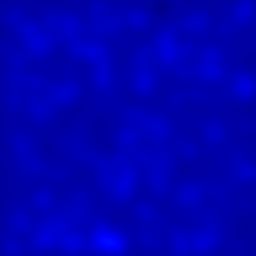

Supplement: S8 File — (ZIP) [file pone.0158590.s008.zip › movie_normal-2-original/movie_normal-2-original257.jpg]

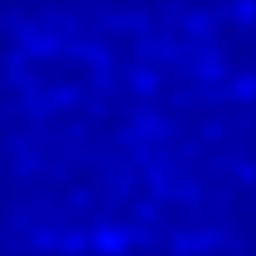

Supplement: S8 File — (ZIP) [file pone.0158590.s008.zip › movie_normal-2-original/movie_normal-2-original258.jpg]

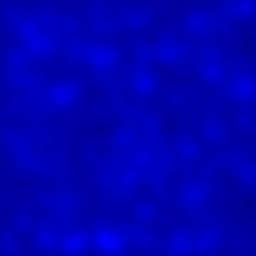

Supplement: S8 File — (ZIP) [file pone.0158590.s008.zip › movie_normal-2-original/movie_normal-2-original259.jpg]

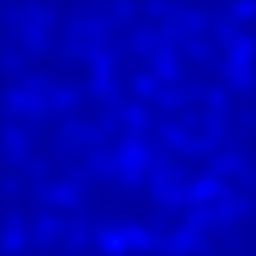

Supplement: S8 File — (ZIP) [file pone.0158590.s008.zip › movie_normal-2-original/movie_normal-2-original26.jpg]

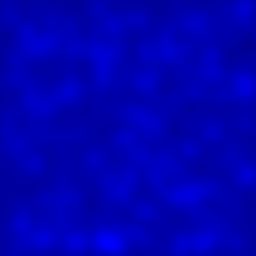

Supplement: S8 File — (ZIP) [file pone.0158590.s008.zip › movie_normal-2-original/movie_normal-2-original260.jpg]

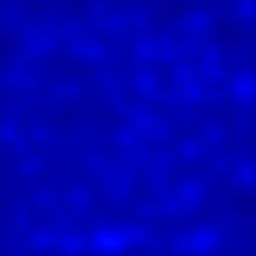

Supplement: S8 File — (ZIP) [file pone.0158590.s008.zip › movie_normal-2-original/movie_normal-2-original261.jpg]

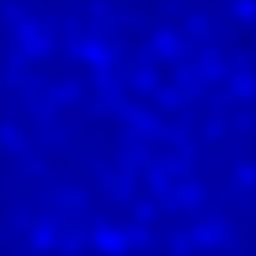

Supplement: S8 File — (ZIP) [file pone.0158590.s008.zip › movie_normal-2-original/movie_normal-2-original262.jpg]

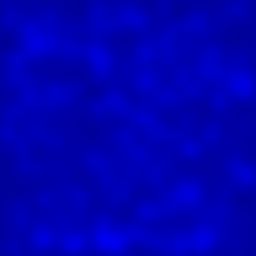

Supplement: S8 File — (ZIP) [file pone.0158590.s008.zip › movie_normal-2-original/movie_normal-2-original263.jpg]

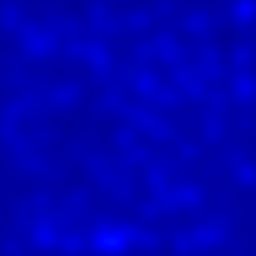

Supplement: S8 File — (ZIP) [file pone.0158590.s008.zip › movie_normal-2-original/movie_normal-2-original264.jpg]

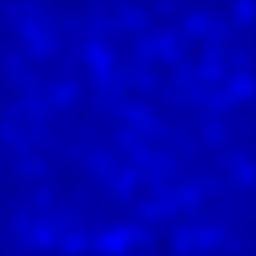

Supplement: S8 File — (ZIP) [file pone.0158590.s008.zip › movie_normal-2-original/movie_normal-2-original265.jpg]

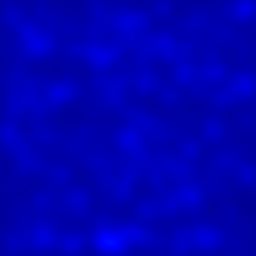

Supplement: S8 File — (ZIP) [file pone.0158590.s008.zip › movie_normal-2-original/movie_normal-2-original266.jpg]

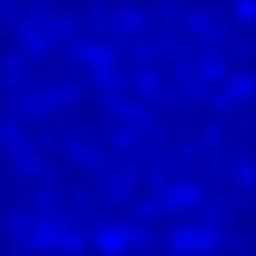

Supplement: S8 File — (ZIP) [file pone.0158590.s008.zip › movie_normal-2-original/movie_normal-2-original267.jpg]

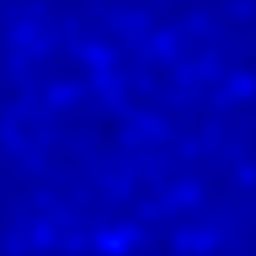

Supplement: S8 File — (ZIP) [file pone.0158590.s008.zip › movie_normal-2-original/movie_normal-2-original268.jpg]

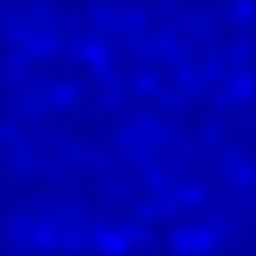

Supplement: S8 File — (ZIP) [file pone.0158590.s008.zip › movie_normal-2-original/movie_normal-2-original269.jpg]

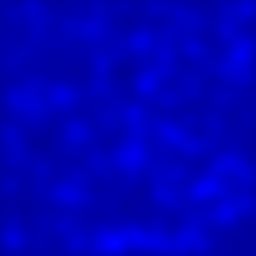

Supplement: S8 File — (ZIP) [file pone.0158590.s008.zip › movie_normal-2-original/movie_normal-2-original27.jpg]

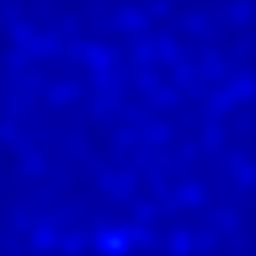

Supplement: S8 File — (ZIP) [file pone.0158590.s008.zip › movie_normal-2-original/movie_normal-2-original270.jpg]

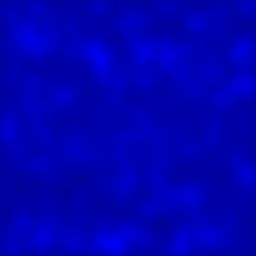

Supplement: S8 File — (ZIP) [file pone.0158590.s008.zip › movie_normal-2-original/movie_normal-2-original271.jpg]

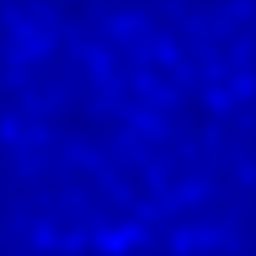

Supplement: S8 File — (ZIP) [file pone.0158590.s008.zip › movie_normal-2-original/movie_normal-2-original272.jpg]

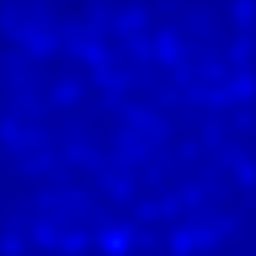

Supplement: S8 File — (ZIP) [file pone.0158590.s008.zip › movie_normal-2-original/movie_normal-2-original273.jpg]

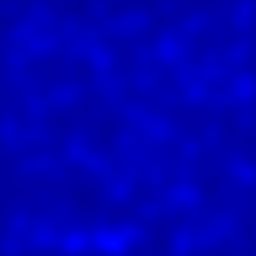

Supplement: S8 File — (ZIP) [file pone.0158590.s008.zip › movie_normal-2-original/movie_normal-2-original274.jpg]

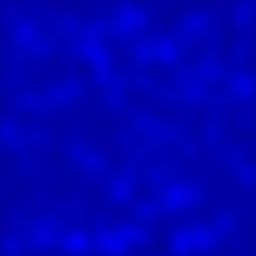

Supplement: S8 File — (ZIP) [file pone.0158590.s008.zip › movie_normal-2-original/movie_normal-2-original275.jpg]

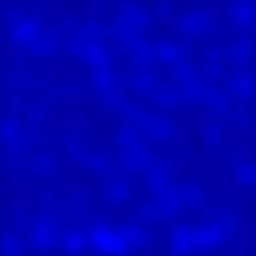

Supplement: S8 File — (ZIP) [file pone.0158590.s008.zip › movie_normal-2-original/movie_normal-2-original276.jpg]

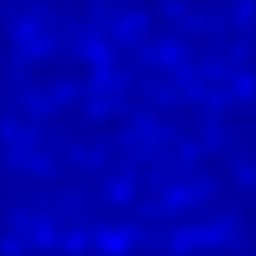

Supplement: S8 File — (ZIP) [file pone.0158590.s008.zip › movie_normal-2-original/movie_normal-2-original277.jpg]

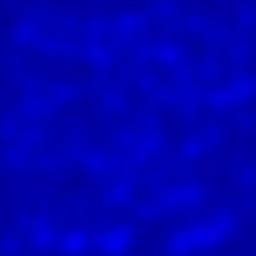

Supplement: S8 File — (ZIP) [file pone.0158590.s008.zip › movie_normal-2-original/movie_normal-2-original278.jpg]

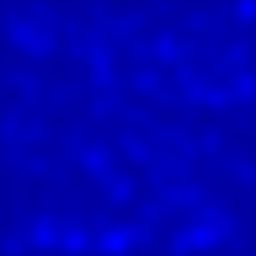

Supplement: S8 File — (ZIP) [file pone.0158590.s008.zip › movie_normal-2-original/movie_normal-2-original279.jpg]
