# Supplementary figures and images for: Invisible Brain: Knowledge in Research Works and Neuron Activity (part 5 of 6)
Source: PLoS One. 2016 Jul 20;11(7):e0158590. doi: 10.1371/journal.pone.0158590 (PMC4954711; doi:10.1371/journal.pone.0158590)

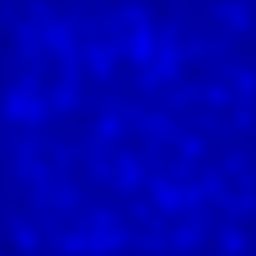

Supplement: S8 File — (ZIP) [file pone.0158590.s008.zip › movie_normal-2-original/movie_normal-2-original108.jpg]

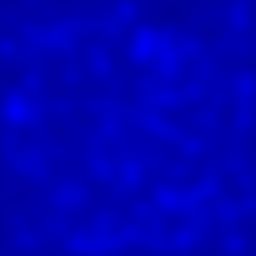

Supplement: S8 File — (ZIP) [file pone.0158590.s008.zip › movie_normal-2-original/movie_normal-2-original109.jpg]

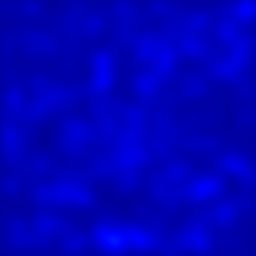

Supplement: S8 File — (ZIP) [file pone.0158590.s008.zip › movie_normal-2-original/movie_normal-2-original11.jpg]

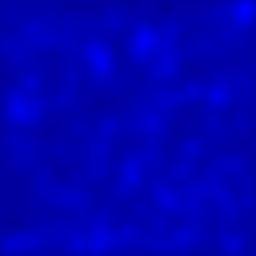

Supplement: S8 File — (ZIP) [file pone.0158590.s008.zip › movie_normal-2-original/movie_normal-2-original110.jpg]

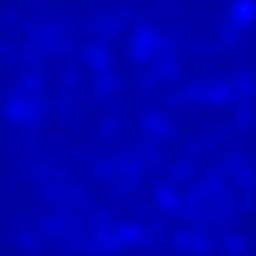

Supplement: S8 File — (ZIP) [file pone.0158590.s008.zip › movie_normal-2-original/movie_normal-2-original111.jpg]

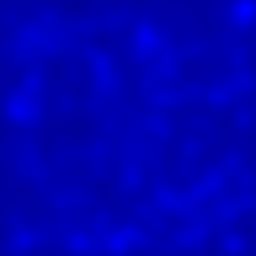

Supplement: S8 File — (ZIP) [file pone.0158590.s008.zip › movie_normal-2-original/movie_normal-2-original112.jpg]

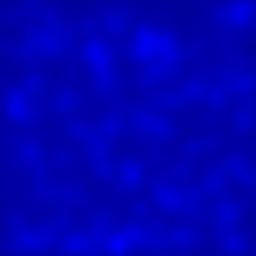

Supplement: S8 File — (ZIP) [file pone.0158590.s008.zip › movie_normal-2-original/movie_normal-2-original113.jpg]

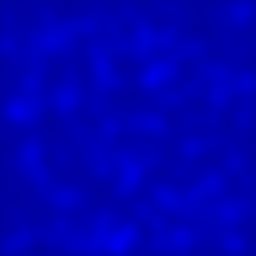

Supplement: S8 File — (ZIP) [file pone.0158590.s008.zip › movie_normal-2-original/movie_normal-2-original114.jpg]

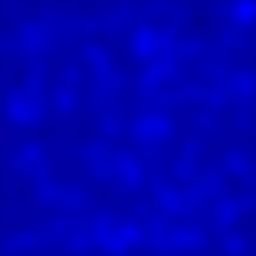

Supplement: S8 File — (ZIP) [file pone.0158590.s008.zip › movie_normal-2-original/movie_normal-2-original115.jpg]

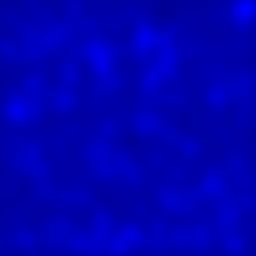

Supplement: S8 File — (ZIP) [file pone.0158590.s008.zip › movie_normal-2-original/movie_normal-2-original116.jpg]

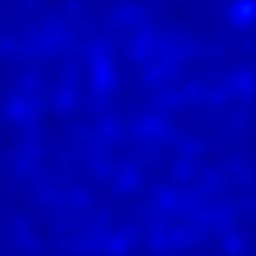

Supplement: S8 File — (ZIP) [file pone.0158590.s008.zip › movie_normal-2-original/movie_normal-2-original117.jpg]

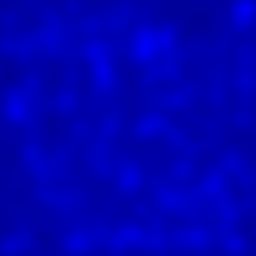

Supplement: S8 File — (ZIP) [file pone.0158590.s008.zip › movie_normal-2-original/movie_normal-2-original118.jpg]

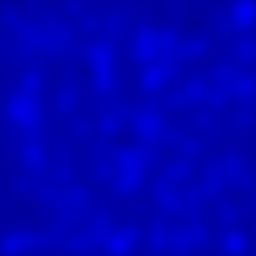

Supplement: S8 File — (ZIP) [file pone.0158590.s008.zip › movie_normal-2-original/movie_normal-2-original119.jpg]

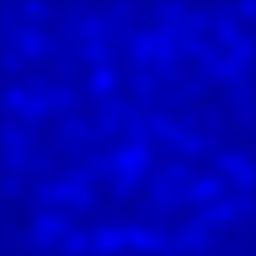

Supplement: S8 File — (ZIP) [file pone.0158590.s008.zip › movie_normal-2-original/movie_normal-2-original12.jpg]

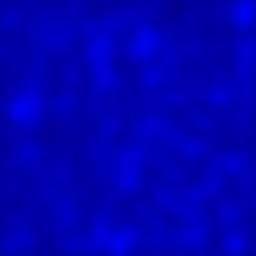

Supplement: S8 File — (ZIP) [file pone.0158590.s008.zip › movie_normal-2-original/movie_normal-2-original120.jpg]

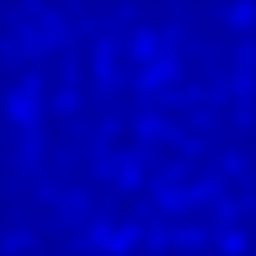

Supplement: S8 File — (ZIP) [file pone.0158590.s008.zip › movie_normal-2-original/movie_normal-2-original121.jpg]

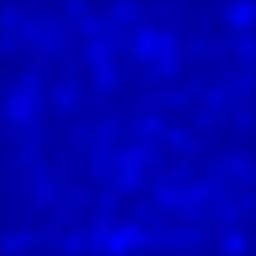

Supplement: S8 File — (ZIP) [file pone.0158590.s008.zip › movie_normal-2-original/movie_normal-2-original122.jpg]

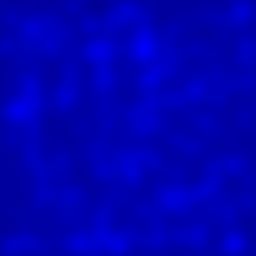

Supplement: S8 File — (ZIP) [file pone.0158590.s008.zip › movie_normal-2-original/movie_normal-2-original123.jpg]

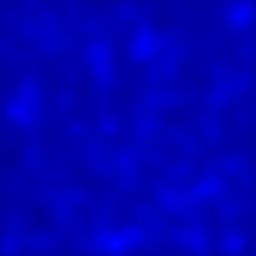

Supplement: S8 File — (ZIP) [file pone.0158590.s008.zip › movie_normal-2-original/movie_normal-2-original124.jpg]

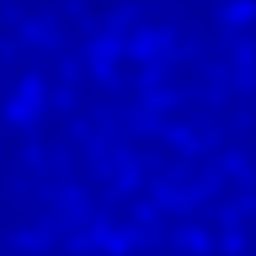

Supplement: S8 File — (ZIP) [file pone.0158590.s008.zip › movie_normal-2-original/movie_normal-2-original125.jpg]

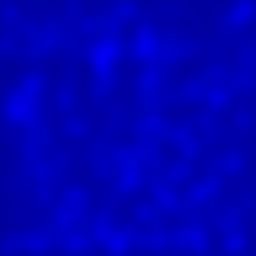

Supplement: S8 File — (ZIP) [file pone.0158590.s008.zip › movie_normal-2-original/movie_normal-2-original126.jpg]

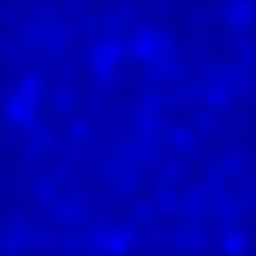

Supplement: S8 File — (ZIP) [file pone.0158590.s008.zip › movie_normal-2-original/movie_normal-2-original127.jpg]

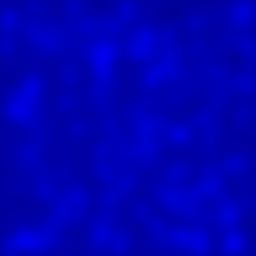

Supplement: S8 File — (ZIP) [file pone.0158590.s008.zip › movie_normal-2-original/movie_normal-2-original128.jpg]

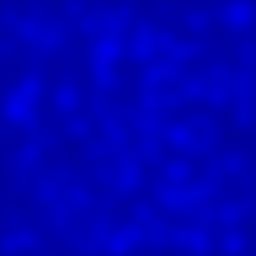

Supplement: S8 File — (ZIP) [file pone.0158590.s008.zip › movie_normal-2-original/movie_normal-2-original129.jpg]

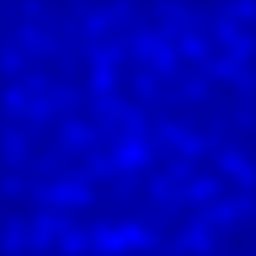

Supplement: S8 File — (ZIP) [file pone.0158590.s008.zip › movie_normal-2-original/movie_normal-2-original13.jpg]

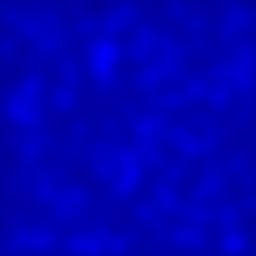

Supplement: S8 File — (ZIP) [file pone.0158590.s008.zip › movie_normal-2-original/movie_normal-2-original130.jpg]

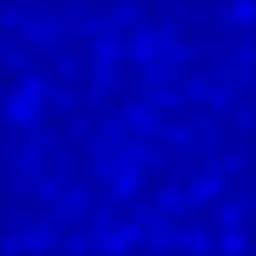

Supplement: S8 File — (ZIP) [file pone.0158590.s008.zip › movie_normal-2-original/movie_normal-2-original131.jpg]

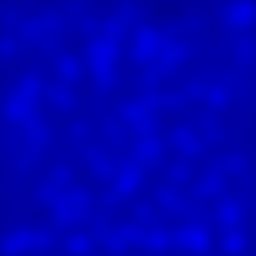

Supplement: S8 File — (ZIP) [file pone.0158590.s008.zip › movie_normal-2-original/movie_normal-2-original132.jpg]

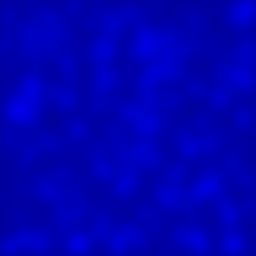

Supplement: S8 File — (ZIP) [file pone.0158590.s008.zip › movie_normal-2-original/movie_normal-2-original133.jpg]

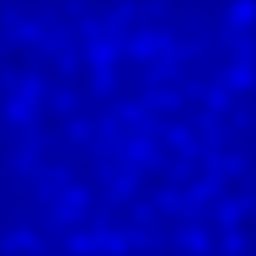

Supplement: S8 File — (ZIP) [file pone.0158590.s008.zip › movie_normal-2-original/movie_normal-2-original134.jpg]

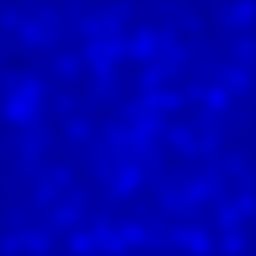

Supplement: S8 File — (ZIP) [file pone.0158590.s008.zip › movie_normal-2-original/movie_normal-2-original135.jpg]

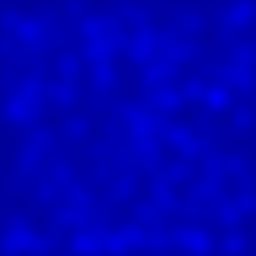

Supplement: S8 File — (ZIP) [file pone.0158590.s008.zip › movie_normal-2-original/movie_normal-2-original136.jpg]

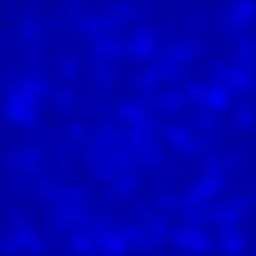

Supplement: S8 File — (ZIP) [file pone.0158590.s008.zip › movie_normal-2-original/movie_normal-2-original137.jpg]

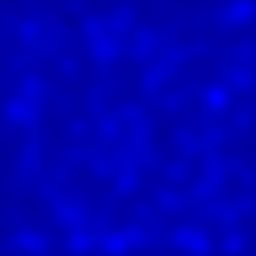

Supplement: S8 File — (ZIP) [file pone.0158590.s008.zip › movie_normal-2-original/movie_normal-2-original138.jpg]

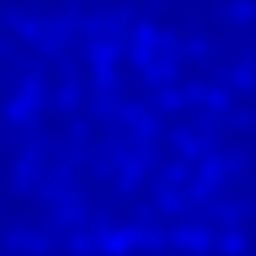

Supplement: S8 File — (ZIP) [file pone.0158590.s008.zip › movie_normal-2-original/movie_normal-2-original139.jpg]

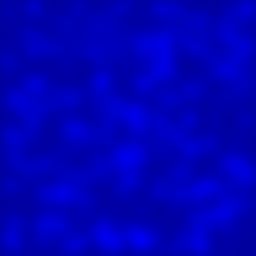

Supplement: S8 File — (ZIP) [file pone.0158590.s008.zip › movie_normal-2-original/movie_normal-2-original14.jpg]

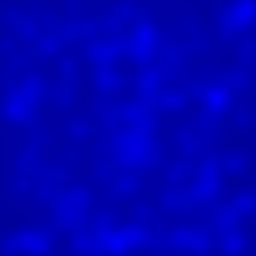

Supplement: S8 File — (ZIP) [file pone.0158590.s008.zip › movie_normal-2-original/movie_normal-2-original140.jpg]

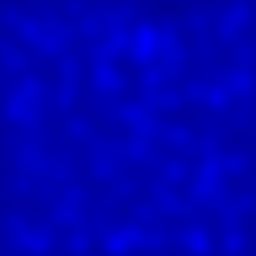

Supplement: S8 File — (ZIP) [file pone.0158590.s008.zip › movie_normal-2-original/movie_normal-2-original141.jpg]

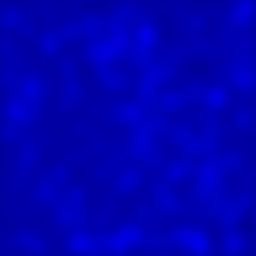

Supplement: S8 File — (ZIP) [file pone.0158590.s008.zip › movie_normal-2-original/movie_normal-2-original142.jpg]

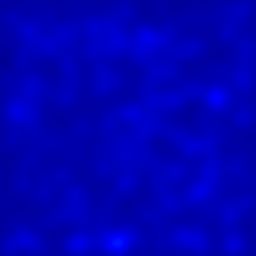

Supplement: S8 File — (ZIP) [file pone.0158590.s008.zip › movie_normal-2-original/movie_normal-2-original143.jpg]

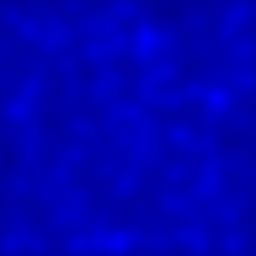

Supplement: S8 File — (ZIP) [file pone.0158590.s008.zip › movie_normal-2-original/movie_normal-2-original144.jpg]

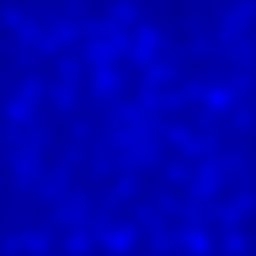

Supplement: S8 File — (ZIP) [file pone.0158590.s008.zip › movie_normal-2-original/movie_normal-2-original145.jpg]

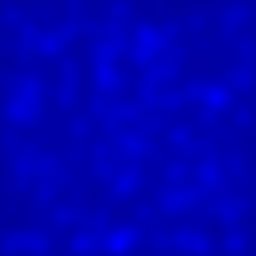

Supplement: S8 File — (ZIP) [file pone.0158590.s008.zip › movie_normal-2-original/movie_normal-2-original146.jpg]

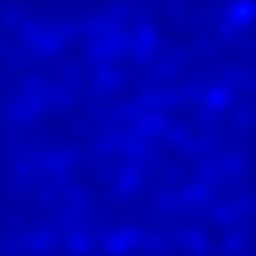

Supplement: S8 File — (ZIP) [file pone.0158590.s008.zip › movie_normal-2-original/movie_normal-2-original147.jpg]

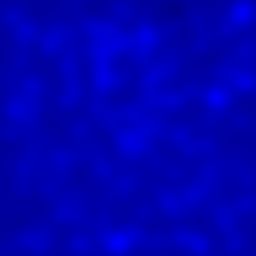

Supplement: S8 File — (ZIP) [file pone.0158590.s008.zip › movie_normal-2-original/movie_normal-2-original148.jpg]

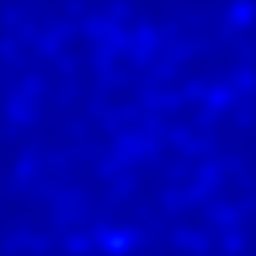

Supplement: S8 File — (ZIP) [file pone.0158590.s008.zip › movie_normal-2-original/movie_normal-2-original149.jpg]

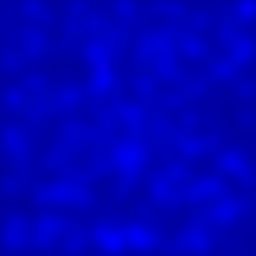

Supplement: S8 File — (ZIP) [file pone.0158590.s008.zip › movie_normal-2-original/movie_normal-2-original15.jpg]

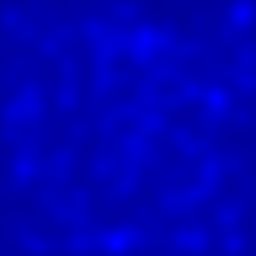

Supplement: S8 File — (ZIP) [file pone.0158590.s008.zip › movie_normal-2-original/movie_normal-2-original150.jpg]

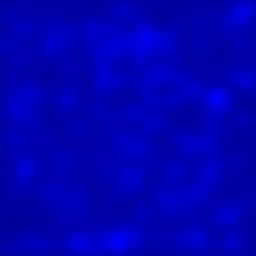

Supplement: S8 File — (ZIP) [file pone.0158590.s008.zip › movie_normal-2-original/movie_normal-2-original151.jpg]

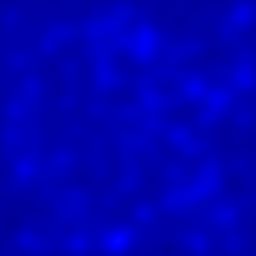

Supplement: S8 File — (ZIP) [file pone.0158590.s008.zip › movie_normal-2-original/movie_normal-2-original152.jpg]

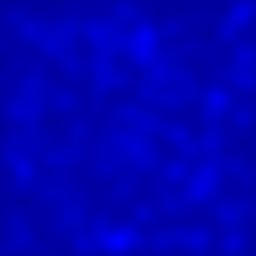

Supplement: S8 File — (ZIP) [file pone.0158590.s008.zip › movie_normal-2-original/movie_normal-2-original153.jpg]

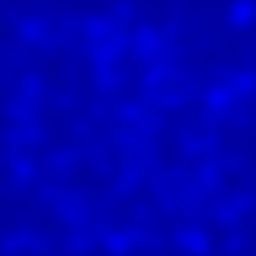

Supplement: S8 File — (ZIP) [file pone.0158590.s008.zip › movie_normal-2-original/movie_normal-2-original154.jpg]

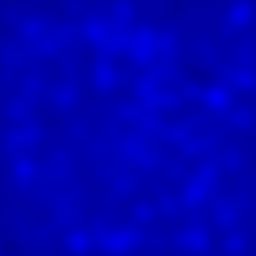

Supplement: S8 File — (ZIP) [file pone.0158590.s008.zip › movie_normal-2-original/movie_normal-2-original155.jpg]

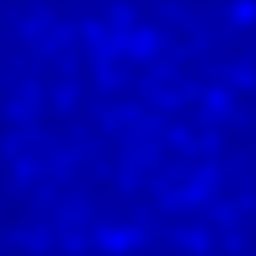

Supplement: S8 File — (ZIP) [file pone.0158590.s008.zip › movie_normal-2-original/movie_normal-2-original156.jpg]

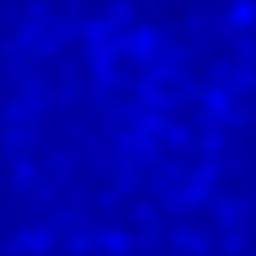

Supplement: S8 File — (ZIP) [file pone.0158590.s008.zip › movie_normal-2-original/movie_normal-2-original157.jpg]

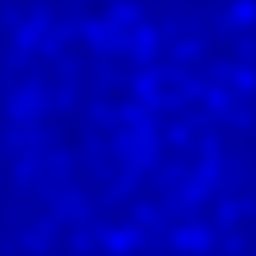

Supplement: S8 File — (ZIP) [file pone.0158590.s008.zip › movie_normal-2-original/movie_normal-2-original158.jpg]

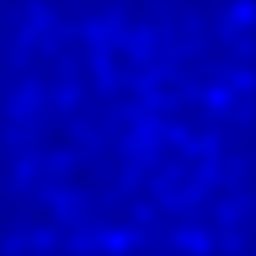

Supplement: S8 File — (ZIP) [file pone.0158590.s008.zip › movie_normal-2-original/movie_normal-2-original159.jpg]

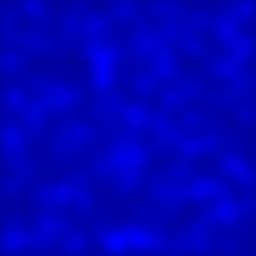

Supplement: S8 File — (ZIP) [file pone.0158590.s008.zip › movie_normal-2-original/movie_normal-2-original16.jpg]

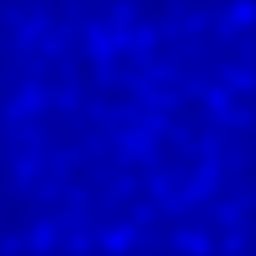

Supplement: S8 File — (ZIP) [file pone.0158590.s008.zip › movie_normal-2-original/movie_normal-2-original160.jpg]

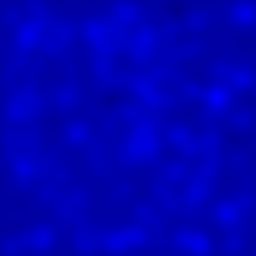

Supplement: S8 File — (ZIP) [file pone.0158590.s008.zip › movie_normal-2-original/movie_normal-2-original161.jpg]

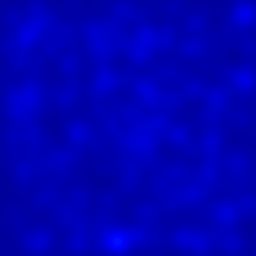

Supplement: S8 File — (ZIP) [file pone.0158590.s008.zip › movie_normal-2-original/movie_normal-2-original162.jpg]

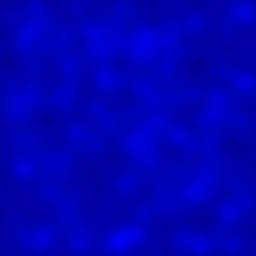

Supplement: S8 File — (ZIP) [file pone.0158590.s008.zip › movie_normal-2-original/movie_normal-2-original163.jpg]

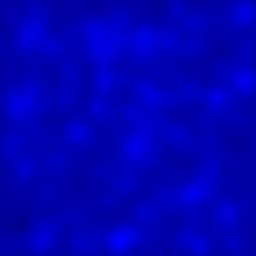

Supplement: S8 File — (ZIP) [file pone.0158590.s008.zip › movie_normal-2-original/movie_normal-2-original164.jpg]

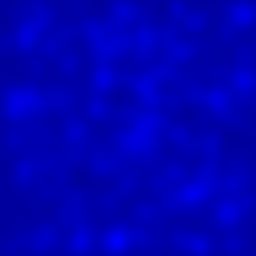

Supplement: S8 File — (ZIP) [file pone.0158590.s008.zip › movie_normal-2-original/movie_normal-2-original165.jpg]

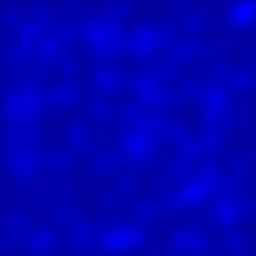

Supplement: S8 File — (ZIP) [file pone.0158590.s008.zip › movie_normal-2-original/movie_normal-2-original166.jpg]

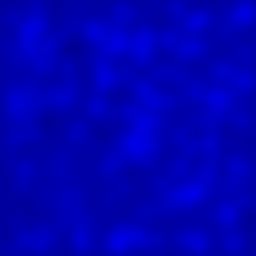

Supplement: S8 File — (ZIP) [file pone.0158590.s008.zip › movie_normal-2-original/movie_normal-2-original167.jpg]

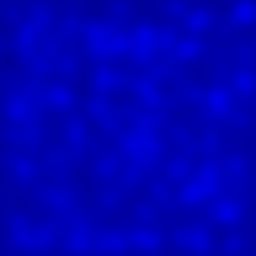

Supplement: S8 File — (ZIP) [file pone.0158590.s008.zip › movie_normal-2-original/movie_normal-2-original168.jpg]

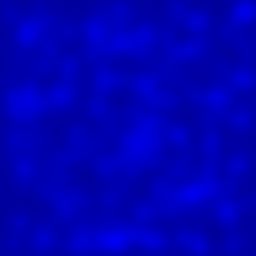

Supplement: S8 File — (ZIP) [file pone.0158590.s008.zip › movie_normal-2-original/movie_normal-2-original169.jpg]

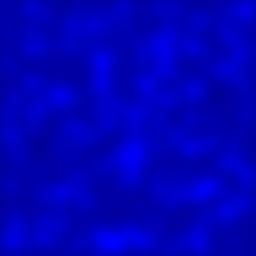

Supplement: S8 File — (ZIP) [file pone.0158590.s008.zip › movie_normal-2-original/movie_normal-2-original17.jpg]

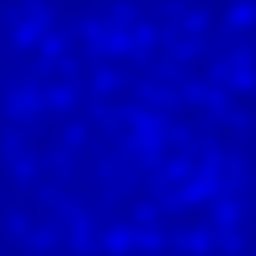

Supplement: S8 File — (ZIP) [file pone.0158590.s008.zip › movie_normal-2-original/movie_normal-2-original170.jpg]

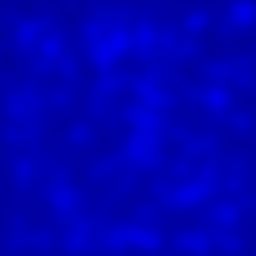

Supplement: S8 File — (ZIP) [file pone.0158590.s008.zip › movie_normal-2-original/movie_normal-2-original171.jpg]

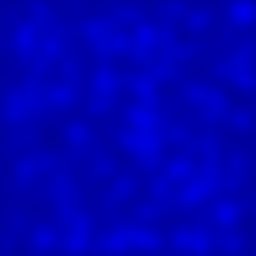

Supplement: S8 File — (ZIP) [file pone.0158590.s008.zip › movie_normal-2-original/movie_normal-2-original172.jpg]

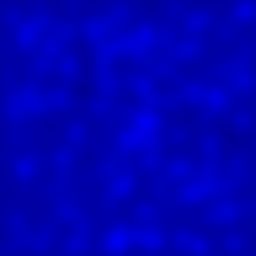

Supplement: S8 File — (ZIP) [file pone.0158590.s008.zip › movie_normal-2-original/movie_normal-2-original173.jpg]

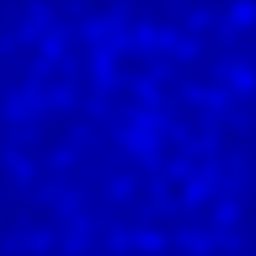

Supplement: S8 File — (ZIP) [file pone.0158590.s008.zip › movie_normal-2-original/movie_normal-2-original174.jpg]

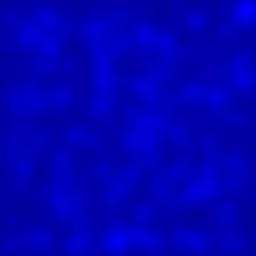

Supplement: S8 File — (ZIP) [file pone.0158590.s008.zip › movie_normal-2-original/movie_normal-2-original175.jpg]

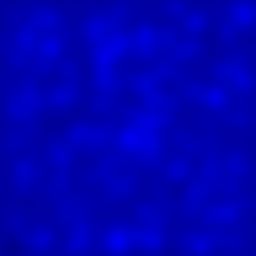

Supplement: S8 File — (ZIP) [file pone.0158590.s008.zip › movie_normal-2-original/movie_normal-2-original176.jpg]

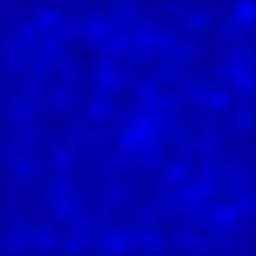

Supplement: S8 File — (ZIP) [file pone.0158590.s008.zip › movie_normal-2-original/movie_normal-2-original177.jpg]

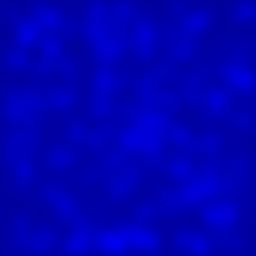

Supplement: S8 File — (ZIP) [file pone.0158590.s008.zip › movie_normal-2-original/movie_normal-2-original178.jpg]

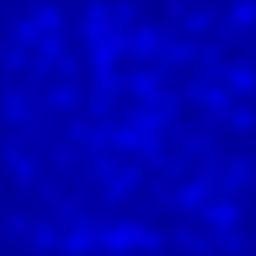

Supplement: S8 File — (ZIP) [file pone.0158590.s008.zip › movie_normal-2-original/movie_normal-2-original179.jpg]

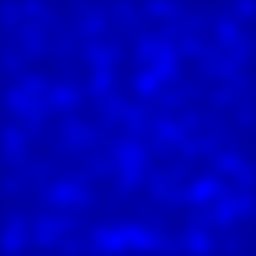

Supplement: S8 File — (ZIP) [file pone.0158590.s008.zip › movie_normal-2-original/movie_normal-2-original18.jpg]

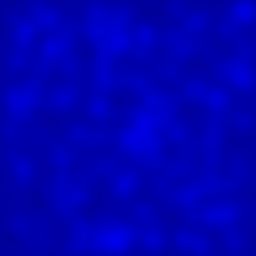

Supplement: S8 File — (ZIP) [file pone.0158590.s008.zip › movie_normal-2-original/movie_normal-2-original180.jpg]

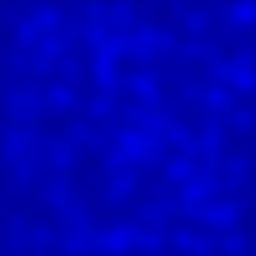

Supplement: S8 File — (ZIP) [file pone.0158590.s008.zip › movie_normal-2-original/movie_normal-2-original181.jpg]

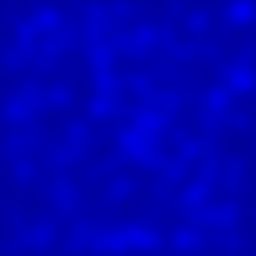

Supplement: S8 File — (ZIP) [file pone.0158590.s008.zip › movie_normal-2-original/movie_normal-2-original182.jpg]

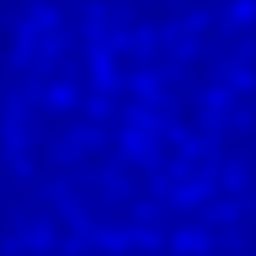

Supplement: S8 File — (ZIP) [file pone.0158590.s008.zip › movie_normal-2-original/movie_normal-2-original183.jpg]

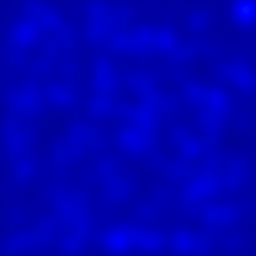

Supplement: S8 File — (ZIP) [file pone.0158590.s008.zip › movie_normal-2-original/movie_normal-2-original184.jpg]

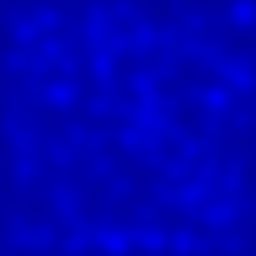

Supplement: S8 File — (ZIP) [file pone.0158590.s008.zip › movie_normal-2-original/movie_normal-2-original185.jpg]

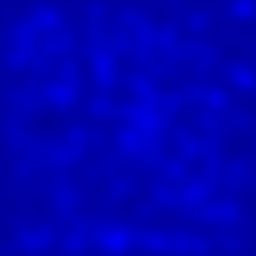

Supplement: S8 File — (ZIP) [file pone.0158590.s008.zip › movie_normal-2-original/movie_normal-2-original186.jpg]

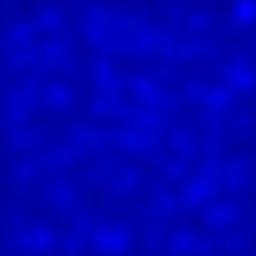

Supplement: S8 File — (ZIP) [file pone.0158590.s008.zip › movie_normal-2-original/movie_normal-2-original187.jpg]

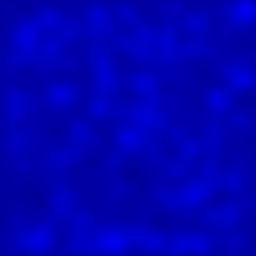

Supplement: S8 File — (ZIP) [file pone.0158590.s008.zip › movie_normal-2-original/movie_normal-2-original188.jpg]

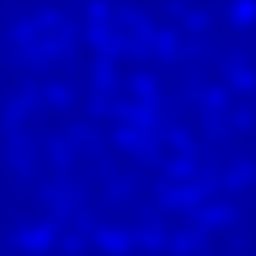

Supplement: S8 File — (ZIP) [file pone.0158590.s008.zip › movie_normal-2-original/movie_normal-2-original189.jpg]

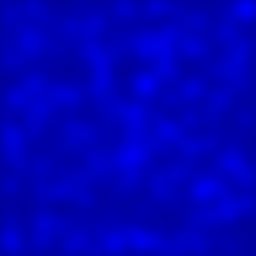

Supplement: S8 File — (ZIP) [file pone.0158590.s008.zip › movie_normal-2-original/movie_normal-2-original19.jpg]

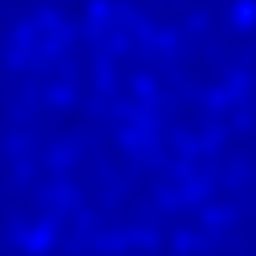

Supplement: S8 File — (ZIP) [file pone.0158590.s008.zip › movie_normal-2-original/movie_normal-2-original190.jpg]

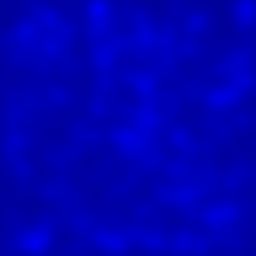

Supplement: S8 File — (ZIP) [file pone.0158590.s008.zip › movie_normal-2-original/movie_normal-2-original191.jpg]

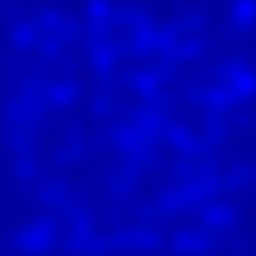

Supplement: S8 File — (ZIP) [file pone.0158590.s008.zip › movie_normal-2-original/movie_normal-2-original192.jpg]

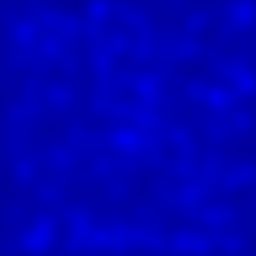

Supplement: S8 File — (ZIP) [file pone.0158590.s008.zip › movie_normal-2-original/movie_normal-2-original193.jpg]

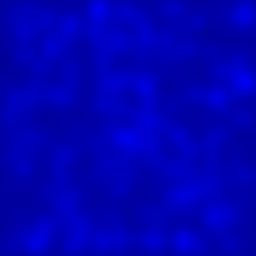

Supplement: S8 File — (ZIP) [file pone.0158590.s008.zip › movie_normal-2-original/movie_normal-2-original194.jpg]

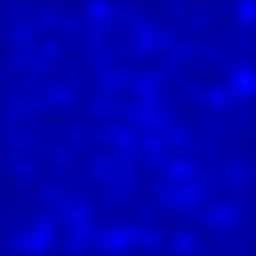

Supplement: S8 File — (ZIP) [file pone.0158590.s008.zip › movie_normal-2-original/movie_normal-2-original195.jpg]

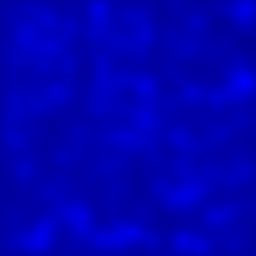

Supplement: S8 File — (ZIP) [file pone.0158590.s008.zip › movie_normal-2-original/movie_normal-2-original196.jpg]

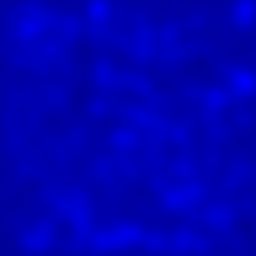

Supplement: S8 File — (ZIP) [file pone.0158590.s008.zip › movie_normal-2-original/movie_normal-2-original197.jpg]

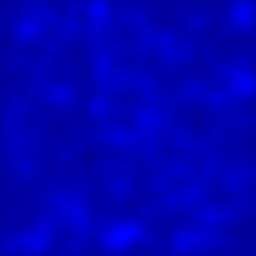

Supplement: S8 File — (ZIP) [file pone.0158590.s008.zip › movie_normal-2-original/movie_normal-2-original198.jpg]
